# Synthesis and properties of tetrathiafulvalenes bearing 6-aryl-1,4-dithiafulvenes

**DOI:** 10.3762/bjoc.16.86

**Published:** 2020-05-12

**Authors:** Aya Yoshimura, Hitoshi Kimura, Kohei Kagawa, Mayuka Yoshioka, Toshiki Itou, Dhananjayan Vasu, Takashi Shirahata, Hideki Yorimitsu, Yohji Misaki

**Affiliations:** 1 Department of Materials Science and Biotechnology, Graduate School of Science and Engineering, Ehime University, 3 Bunkyo-cho, Matsuyama, Ehime 790-8577, Japan; 2 Research Unit for Power Generation and Storage Materials, and Research Unit for Development of Organic Superconductors, Ehime University, 3 Bunkyo-cho, Matsuyama, Ehime 790-8577, Japan; 3 Department of Chemistry, Graduate School of Science, Kyoto University, Sakyo-ku, Kyoto 606-8502, Japan

**Keywords:** cross-conjugated systems, electrochemical properties, extended π-conjugation, digital simulation analysis, tetrathiafulvalene

## Abstract

Novel multistage redox tetrathiafulvalenes (TTFs) bearing 6-aryl-1,4-dithiafulvene moieties were synthesized by palladium-catalyzed direct C–H arylation. In the presence of a catalytic amount of Pd(OAc)_2_, P(*t*-Bu_3_)·HBF_4_, and an excess of Cs_2_CO_3_, the C–H arylation of TTF with several aryl bromides bearing 1,3-dithiol-2-ylidenes took place efficiently to produce the corresponding π-conjugated molecules. We also succeeded in the estimation of the oxidation potentials and number of electrons involved in each oxidation step of the obtained compounds by digital simulations.

## Introduction

**Figure 1:**
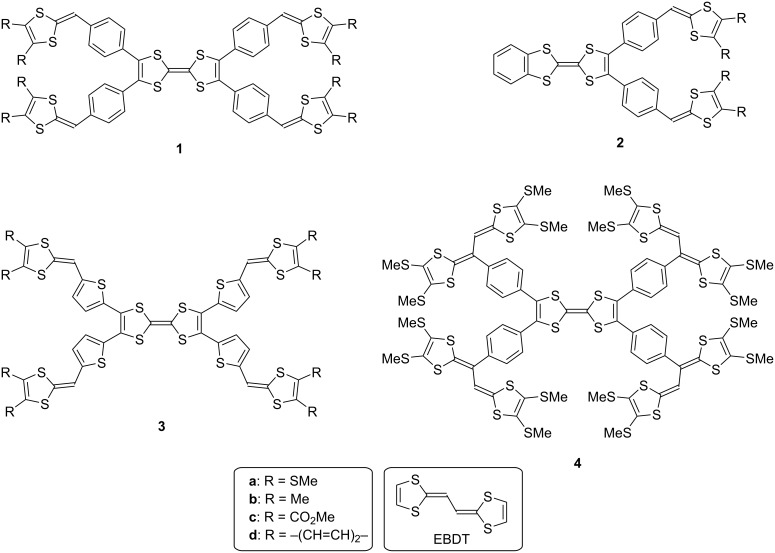
Target compounds.

Tetrathiafulvalenes (TTFs) with extended π-conjugation have attracted attention as possible components of functional materials, such as molecular conductors, field-effect transistors (FETs), and positive electrode materials for rechargeable batteries because the TTF moiety has strong electron-donating properties attributed to the formation of stable aromatic 1,3-dithiol-2-ylidenes (1,3-dithiole rings) by one- and two-electron oxidation [1–16]. Considerable efforts have been devoted to the development of peripherally benzene- or thiophene-substituted TTFs. As for peripherally benzene-functionalized TTFs, some synthetic approaches, crystal and electronic structures, electrochemical and optical properties, and the nature of radical ion complexes with DDQ or iodine were reported [17–24]. Peripherally thiophene-functionalized TTFs, as potential precursors to conducting polymers, and organic metals were also prepared and characterized [25–29]. To design more tempting molecules, the attachment of 1,3-dithiole rings to aromatic rings appears very appealing since these allow to produce novel multistage redox systems. However, such molecules could formerly not be synthesized by conventional approaches. In 2011, a breakthrough synthesis of arylated TTF derivatives by a palladium-catalyzed direct C–H arylation was reported, and the structural and electrochemical properties of the products were clarified [30]. This motivated us to synthesize novel multistage redox-TTFs bearing 1,3-dithiole rings on aromatic rings, **1**–**3** (Figure 1). In addition, we focused on cross-conjugated systems with 1,3-dithiole rings, which are of interest as novel multistage redox systems as well as donor components for organic conductors [1,31–41]. The palladium-catalyzed C–H arylation might offer access to new cross-conjugated molecules bearing vinyl-extended TTF moieties (EBDTs), such as **4** (Figure 1), and the electrochemical properties of these representatives should be brought to light. Herein, we report the synthesis and electrochemical properties of tetrathiafulvalene derivatives **1**–**4**.

## Results and Discussion

### Synthesis

**Scheme 1:**
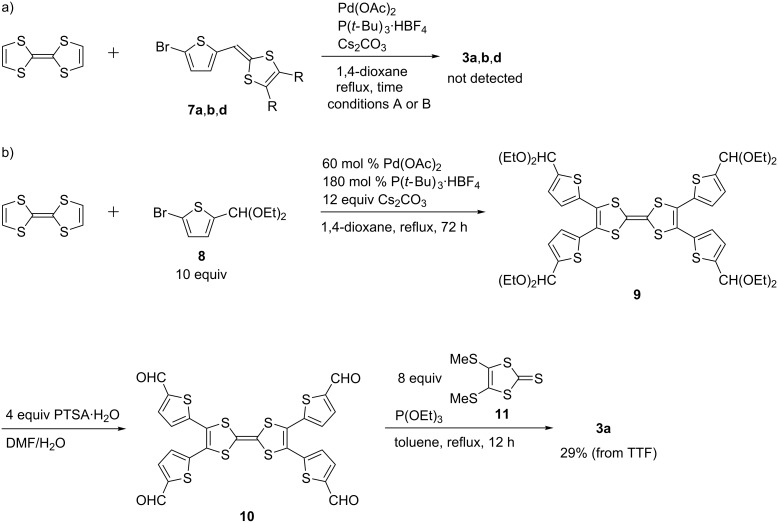
Synthesis of compounds **3**.

**Scheme 2:**
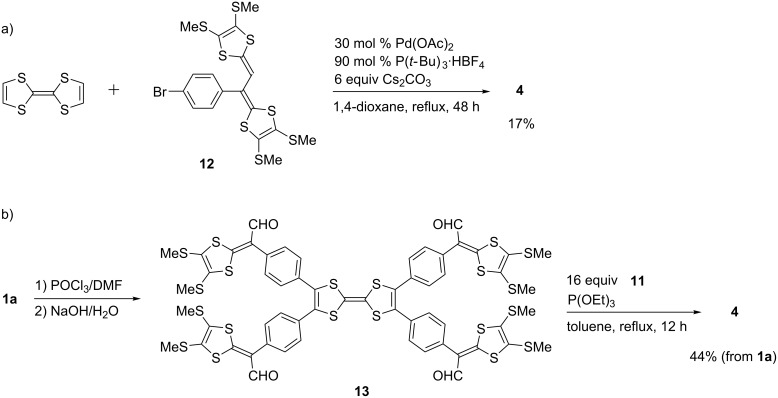
Synthesis of compound **4**.

First, we tried to synthesize compounds **1** and **2** in one step from pristine TTF and **5**, respectively, through palladium-catalyzed C–H arylation (Table 1). When the aryl bromides **6a**,**b** were allowed to react with TTF under the conditions A, the products **1a**,**b** were produced in 46 and 48% yields, respectively (Table 1, entries 1 and 2). Attempted isolations of products **1c** and **1d** failed, despite complete conversions of TTF, because the polarities of the mono-, di-, and triarylated TTFs were extremely close to that of the tetraarylated TTF **1c** and the solubility of these compounds were low and almost beyond isolation for **1d** (Table 1, entries 3 and 4). The palladium-catalyzed C–H arylation of **5** with **6a**,**b** proceeded to give products **2a**,**b** in 75 and 86% yields, respectively (Table 1, entries 5 and 6). On the other hand, it was difficult to produce **3** in the same manner because 2-bromothiophenes **7** bearing a 1,3-dithiole ring at the 5-position were unstable, for example, **7a** decomposed at around 41–42 °C (Scheme 1a). Therefore, we achieved the synthesis of **3a** by Pd-catalyzed thienylation of TTF using acetal-protected **8**, followed by deprotection using PTSA·H_2_O and P(OEt)_3_-mediated cross coupling with **11** (Scheme 1b). The cross-conjugated molecule **4** was prepared in two procedures; one was the palladium-catalyzed C–H arylation of TTF with bromide **12** (Scheme 2a) and the other was the Vilsmeier–Haack reaction of **1a**, followed by triethyl phosphite-mediated cross coupling with **11** (Scheme 2b).

**Table 1:** Synthesis of compounds **1** and **2**.

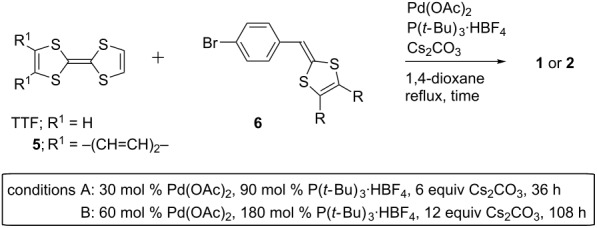				

entry	TTF or **5**	**6** (equiv)	conditions	yield of **1** or **2** (%)

1	TTF	**6a** (5)	A	**1a**: 46
2	TTF	**6b** (5)	A	**1b**: 48
3	TTF	**6c** (5)	A or B	**1c**: 0 (mixture)
4	TTF	**6d** (5)	A or B	**1d**: 0 (mixture)
5	**5**	**6a** (2.5)	A^a^	**2a**: 75
6	**5**	**6b** (2.5)	A^a^	**2b**: 86

^a^Reaction time 24 h.

### Theoretical calculations

**Figure 2:**
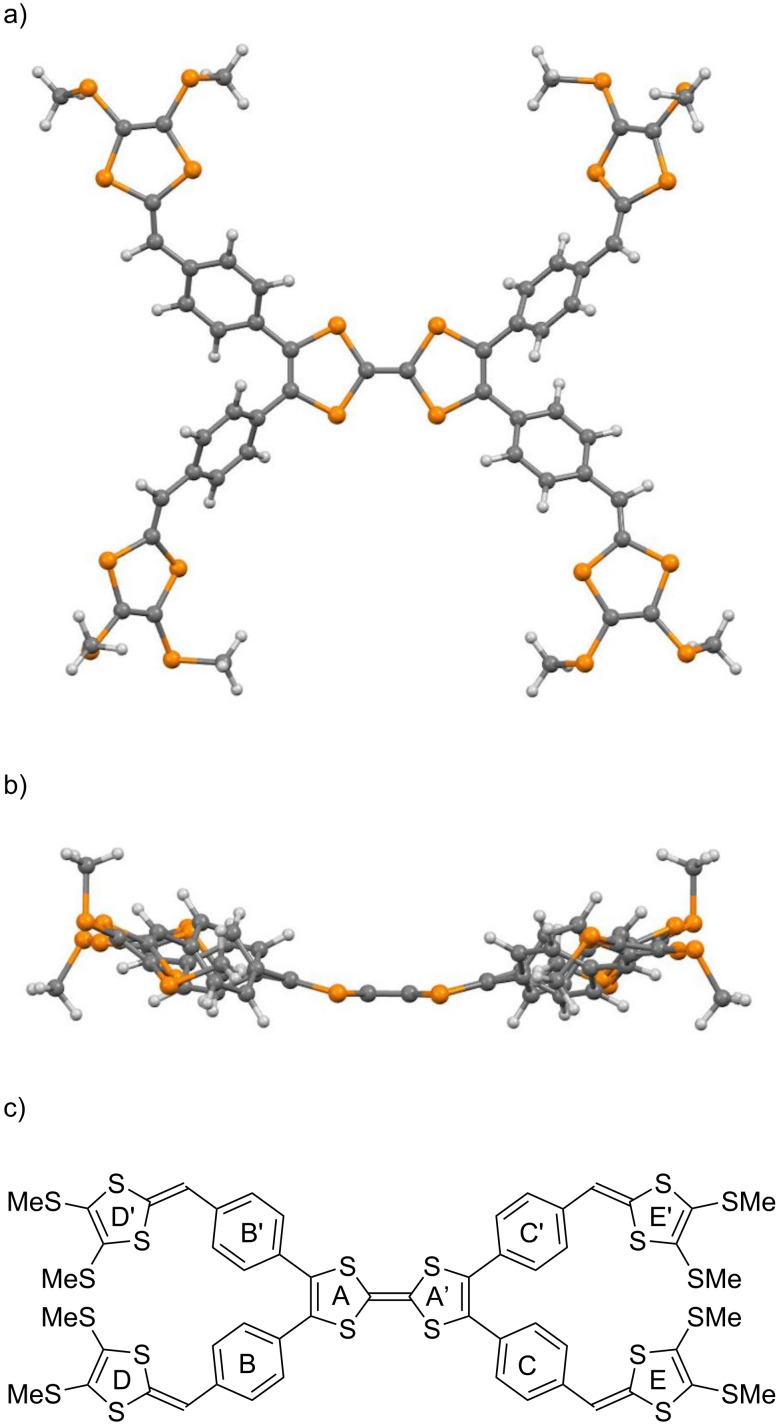
An optimized structure of **1a**. a) Top view, b) side view, and c) labeling of the 1,3-dithiole rings.

**Figure 3:**
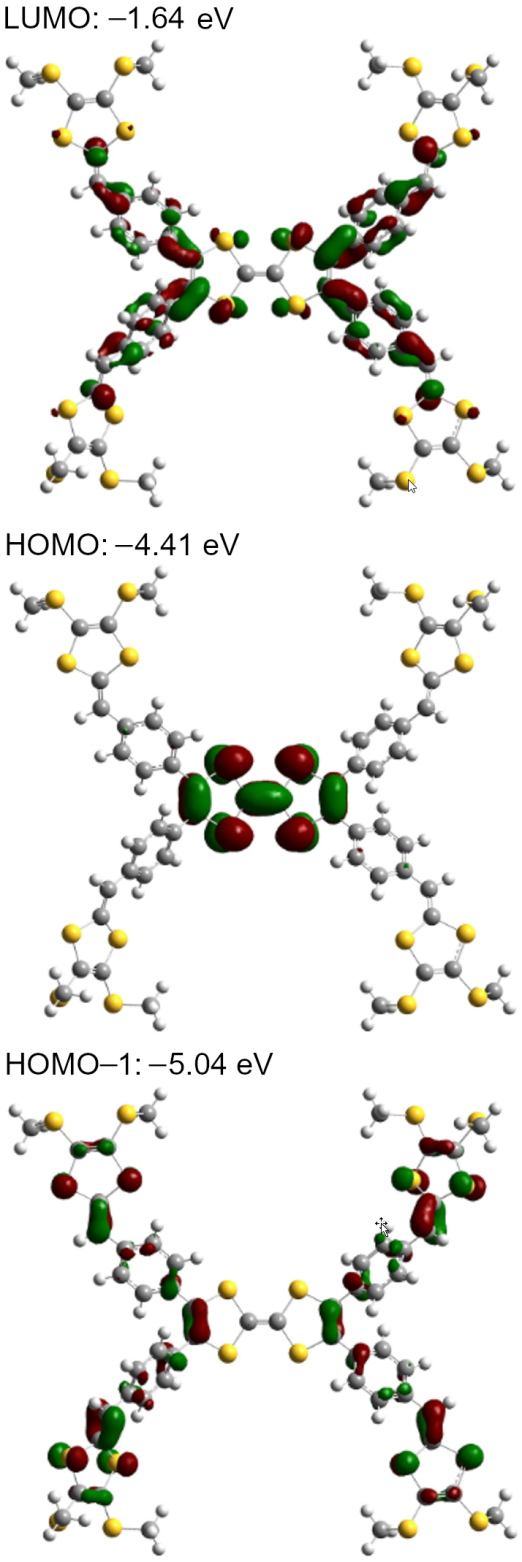
Molecular orbitals of **1a**.

The DFT calculations of compounds **1a**, **3a**, and **4** have been carried out by using the B3LYP/6-31G(d,p) method [42]. Figure 2 shows an optimized geometry of **1a**, and the 1,3-dithiole rings are labeled as A–E and A'–E'. This molecule adopted a nonplanar structure. The angle between the two 1,3-dithiole rings in the center (A–A') was 158.0°. The dihedral angles between A and B, A and B', A' and C, and A' and C' were 49.8°, 137.7°, 137.7°, and 49.9°, respectively. The HOMO, HOMO−1, and LUMO of **1a** are shown in Figure 3. The HOMO of **1a** was mainly located on the TTF moiety, whereas the HOMO−1 was located on the benzene ring and the outer 1,3-dithiole rings at the peripheral part of TTF. The LUMO of **1a** spread over the whole molecule except the outer 1,3-dithiole rings. The orbital energy of the HOMO of **1a** (−4.41 eV) was slightly higher than that of TTF (−4.57 eV). As such, the first oxidation of **1a** might occur at a lower potential than TTF [43].

### Cyclic voltammetry analysis

**Figure 4:**
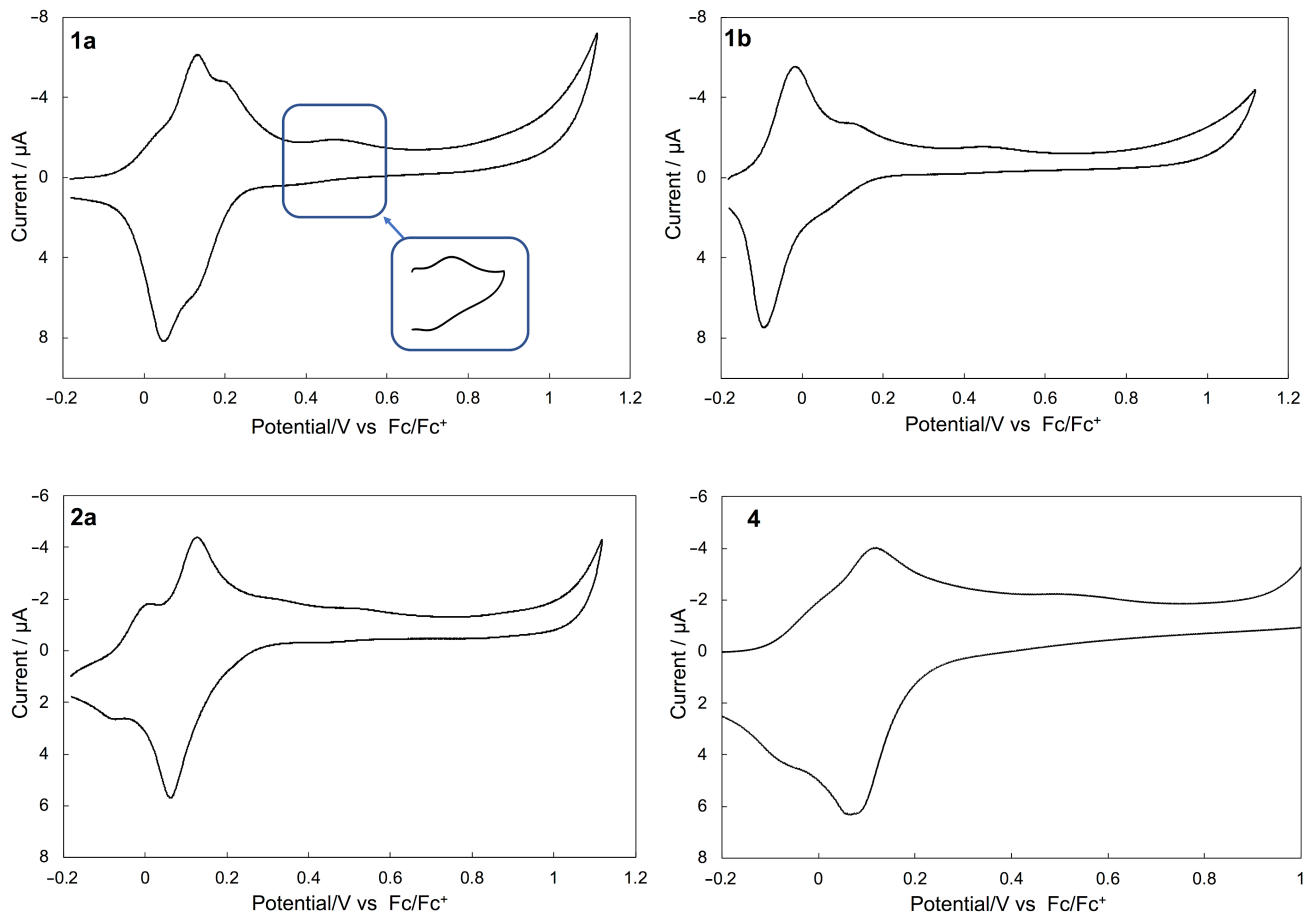
Cyclic voltammograms of **1a**,**b**, **2a**, and **4** in PhCN/CS_2_ 1:1 (v/v) solution.

The redox behavior of **1**–**4** was investigated by cyclic voltammetry. The compounds **1a** and **1b** exhibited four and three pairs of redox waves, respectively (around +0.03, +0.10, +0.17, and +0.42 V vs Fc/Fc^+^ for **1a** and −0.05, +0.10, and +0.46 V vs Fc/Fc^+^ for **1b**, Figure 4). The redox potentials of **1a**,**b** are summarized in Table 2 together with the related compound TTF. The redox waves observed at +0.42 V for **1a** and +0.46 V for **1b** were likely related to the second redox of the central TTF moiety because they were close to the *E*_2_ of TTF (+0.37 V). The remaining redox processes observed at around +0.03, +0.10, and +0.17 V for **1a**, and −0.05 and +0.10 V for **1b** might have been derived from the first redox of the central TTF moiety and the redox of the four outer 1,3-dithiole rings. To elaborate the exact oxidation potentials and the number of electrons involved in each oxidation step, a digital simulation technique was applied [44]. As a result, the observed redox waves of **1a** matched the simulated waves (Table 2). It was indicated that the redox wave at +0.10 V was due to an overlap of the sequential two stages of the one- and two-electron transfer waves at +0.07 and +0.12 V, while the other waves corresponded to one-electron transfer processes. The simulation results of **1a** also showed that the redox wave simulated at +0.020 V might have been derived from the central TTF moiety because of the close Δ*E* values (+0.40 V for **1a** and +0.46 V for TTF). The same discussion was applied to **1b**. In addition, the potentials related to the outer 1,3-dithiole rings of **1a**,**b** were influenced by the substituents, that is, **1b** bearing electron-donating methyl groups had more negative redox potentials than **1a**. As a consequence, the one-electron redox process of the TTF moiety and the multi-electron redox processes of the outer 1,3-dithiole rings might have overlapped in **1b**.

**Table 2:** Redox potentials of **1**, **2a**, **4**, and related compounds^a^.

compound	observed or simulated value	*E*_1_	*E*_2_	*E*_3_	*E*_4_	*E*_5_	*E*_6_	*E*_7_	*E*_8_	*E*_9_	*E*_10_

**1a**	observed	around +0.03^b^	+0.10			+0.17	+0.42				
	simulated	+0.020	+0.070	+0.120		+0.200	+0.420				
**1b**	observed	−0.05				+0.10	+0.46^b^				
**2a**	observed	−0.05	+0.09		+0.49						
**4**	observed	around −0.09			+0.09						+0.53^b^
	simulated	−0.060	−0.030	+0.010	+0.047	+0.053	+0.098	+0.102	+0.110	+0.180	+0.500
TTF^c^	observed	−0.09	+0.37								
**5**^c^	observed	−0.01	+0.42								
**14**^c^	observed	−0.07	+0.09								

^a^In PhCN/CS_2_ 1:1 (v/v) containing 0.1 M *n*-Bu_4_NPF_6_. ^b^Anodic peak. ^c^In PhCN containing 0.1 M *n*-Bu_4_NPF_6_. All potentials were measured against an Ag/Ag^+^ reference electrode and converted to vs Fc/Fc^+^.

Compound **2a** exhibited three pairs of redox waves (−0.05, +0.09, and +0.49 V vs Fc/Fc^+^). The redox waves observed at −0.05 and +0.49 V were likely related to the TTF derivative (**5**) moiety, because they were close to the *E*_1_ and *E*_2_ of **5**, respectively (Table 2). The comparison of the peak currents of each wave indicated that the redox wave observed at +0.09 V involved a two-electron transfer, while the redox waves observed at −0.05 and +0.49 V corresponded to one-electron transfer processes (see the differential pulse voltammetry (DPV) in Supporting Information File 1, Figure S2). These results supported the above-mentioned oxidation potentials and the number of electrons involved in each oxidation step of **1a**,**b**.

**Figure 5:**
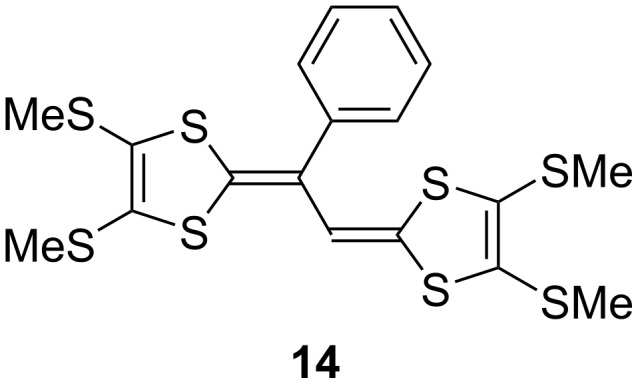
Related compound **14**.

Compound **4** exhibited three pairs of redox waves (around −0.09, +0.09, and +0.53 V vs Fc/Fc^+^). The redox potentials of **4** and the simulation results are also summarized in Table 2, together with those of the related compounds TTF and **14** (see Figure 5). The redox process observed at +0.53 V was likely related to the second redox of the central TTF moiety because this was the closest to the value of the *E*_2_ of TTF (+0.37 V) out of all potentials of the related compounds TTF and **14**. The remaining redox processes observed at around −0.09 and +0.09 V might have been due to the first redox of the central TTF moiety, and the overall redox of the EBDT sites, respectively. The observed potentials of **4** were generally consistent with the simulated ones. The results of a digital simulation also showed that the redox wave observed at around −0.09 V and +0.09 V corresponded to three stages of one-electron transfer and six stages of one-electron transfer processes, respectively. In addition to the overlap of the first redox of the central TTF moiety and the redox of the EBDT sites, each redox potential of the succeeding eight-electron oxidations of the EBDT sites might have slightly shifted due to the non-equivalence of them. Also, the small Δ*E* value (0.16 V) of **14** caused the redox wave overlap. For these reasons, the first and second redox waves of **4** were broad compared to those of **1a** and **1b**.

The redox waves of **1a**,**b** and **4** derived from the second redox of the central TTF moiety (+0.42 V for **1a**, +0.46 V for **1b**, and +0.53 V for **4**) shifted to higher potentials than the second redox of TTF because of the instability of the hexacationic state of **1a**,**b**, and the decacationic state of **4** compared to the dicationic states of TTF caused by on-site Coulomb repulsion between positive charges in the central TTF moiety and the outer 1,3-dithiole rings. The same discussion could be applied to compounds **2a**. In addition, the observed peak currents of **1a** and **4** in the high potential region at +0.4 to +0.5 V were smaller than those of the simulated waves. This phenomenon might be understood by considering that electron transfer of this redox reaction was slow enough to become the rate-determining step because the crowded structure by which the TTF core is participating in this redox process is surrounded by extended aromatic rings bearing 1,3-dithiol rings.

## Conclusion

We have synthesized novel multistage TTF derivatives **1**–**4** bearing 6-aryl-1,4-dithiafulvene moieties by palladium-catalyzed direct C–H arylation. The DFT calculations revealed the nonplanar structure of the compounds. Cyclic voltammograms of **1a** and **4** comprised four and three pairs of redox waves, respectively. As a result of the digital simulation of **1a**, it was shown that the redox wave observed at +0.10 V involved two stages of one- and two-electron transfer(s), while the other redox waves corresponded to one-electron transfer. The digital simulation of **4** showed 10 stages of one-electron transfer in total. In addition, the first and second redox waves of **4** were broad compared to those of **1** owing to the following three reasons: a) overlap of the central TTF moiety and the redox of the EBDT sites, b) the succeeding eight-electron oxidations of the non-equivalent EBDT sites, and c) the small Δ*E* value (0.16 V) of the EBDT sites.

## Supporting Information

File 1 Synthetic procedures, theoretical chemical and electrochemical details, and copies of NMR spectra.
