# Supplementary material for: Synthesis and properties of tetrathiafulvalenes bearing 6-aryl-1,4-dithiafulvenes
Source: Beilstein J Org Chem. 2020 May 12;16:974–81. doi: 10.3762/bjoc.16.86 (PMC7237811; doi:10.3762/bjoc.16.86)
Supplement: File 1 — Synthetic procedures, theoretical chemical and electrochemical details, and copies of NMR spectra. [file Beilstein_J_Org_Chem-16-974-s001.pdf]

BEILSTEIN JOURNAL OF ORGANIC CHEMISTRY

Supporting Information

for

Synthesis and properties of tetrathiafulvalenes bearing 6-aryl-1,4-dithiafulvenes

Aya Yoshimura, Hitoshi Kimura, Kohei Kagawa, Mayuka Yoshioka, Toshiki Itou, Dhananjayan Vasu, Takashi Shirahata, Hideki Yorimitsu and Yohji Misaki

Beilstein J. Org. Chem. **2020,** *16,* 974–981. doi:10.3762/bjoc.16.86

Synthetic procedures, theoretical chemical and electrochemical details, and copies of NMR spectra

License and Terms: This is a supporting information file under the terms of the Creative Commons Attribution License (http://creativecommons.org/licenses/by/4.0). Please note that the reuse, redistribution and reproduction in particular requires that the authors and source are credited. The license is subject to the *Beilstein Journal of Organic Chemistry* terms and conditions: (https://www.beilstein-journals.org/bjoc)

Table of contents

General comments..........S2

Preparation of compounds and spectral data of **1–4, 6, 7, 9, 10, 12, 13**, and **21** S3

Theoretical calculations..........S8

Dihedral angles S10

Optimized geometry..........S12

Cyclic voltammograms..........S24

Results of the digital simulations of **1a** and **4** S25

NMR spectra S27

General comments

Unless otherwise noted, all manipulations were performed under an argon atmosphere and all reagents were purchased from commercial suppliers and used without further purification. Toluene was distilled by standard methods. The products were isolated by silica gel (KANTO KAGAKU Ltd., silica gel 60N 100–210 μm) or alumina gel (Merck Ltd., alumina 90, activated, neutral, activity I, 63–200 μm) column chromatography. ^{1}H NMR and ^{13}C NMR spectra were recorded on a Bruker Biospin AVANCE 400 spectrometer equipped with a CryoProbe (400 MHz for ^{1}H, and 100 MHz for ^{13}C) using $CDCl_3$ or C_6D_6/CS_2 as solvents. The chemical shifts were referenced to tetramethylsilane for ^{1}H and ^{13}C NMR or to the solvent resonances for ^{13}C NMR as internal standards ($CDCl_3$: 77.0 ppm, C_6D_6: 128.0 ppm). Mass spectra were recorded on a JEOL JMS-S3000. Melting points were determined with a Yanaco MP-500D. Cyclic voltammetry (CV) was recorded on an ALS/chi 617B electrochemical analyzer. The CV cell consisted of a Pt working electrode, a Pt wire counter electrode, and $Ag/AgNO_3$ as the reference electrode. The measurements were carried out in benzonitrile with 0.1 M n-$Bu_4N^+PF_6^-$ as a supporting electrolyte, with a scan rate 50 mV/s at 25 °C. All redox potentials were measured against Ag/Ag^+ and converted to vs Fc/Fc^+.

Preparation of compounds 1–4, 6, 7, 9, 10, 12, 13, and 21

Typical procedure for the synthesis of 1, 2, and 4: $Pd(OAc)_2$ (6.8 mg, 0.0303 mmol), P*t*-Bu_3·HBF_4 (26.3 mg, 0.0906 mmol), and Cs_2CO_3 (196.1 mg, 0.602 mmol) were placed in a 30-mL reaction flask under an argon atmosphere. 1,4-Dioxane (2 mL) was added and the mixture was stirred for 10 min at 50 °C, and then, compound **4a** (182.3 mg, 0.502 mmol), and TTF (20.2 mg, 0.0988 mmol) were added. The mixture was heated at 110 °C for 36 h. The organic compounds were extracted with dichloromethane three times. The combined organic layer was washed with H_2O, dried over anhydrous Na_2SO_4, and concentrated in vacuo. The residue was purified by silica gel chromatography with a mixture of dichlorometane/carbon disulfide 2:3 as the eluent to yield **1a** as orange powder (61.3 mg, 46%).

1a: Orange powder; ^{1}H NMR ($CDCl_3$, 400 MHz) δ 2.42 (s 12H), 2.43 (s 12H), 6.41 (s, 4H), 7.07 (d, *J* = 8.0 Hz, 8H), 7.21 (d, *J* = 8.0 Hz, 8H); ^{13}C NMR (C_6D_6-CS_2, 100 MHz) δ19.2, 19.3, 114.3, 125.1, 127.9, 128.3, 129.1, 129.5, 130.3, 131.3, 134.3, 136.4; Mp 112−113 ^{o}C (decomposed); HRMS (MALDI-TOF): m/z calcd for $C_{54}H_{44}S_{20}$: 1331.7857; found: 1331.7732.

1b: Red brown powder; ^{1}H NMR (C_6D_6-CS_2, 400 MHz) δ 1.84 (s 12H), 1.87 (s 12H), 6.18 (s, 4H), 6.95 (d, *J* = 8.0 Hz, 8H), 7.08 (d, *J* = 8.0 Hz, 8H); ^{13}C NMR (C_6D_6-CS_2, 100 MHz) δ 13.0, 13.8, 111.5, 118.4, 120.3, 121.5, 121.9, 126.5, 126.8, 127.0, 129.0, 129.3, 129.4, 136.0, 137.0; Mp 144−145 ^{o}C (decomposed); HRMS (MALDI-TOF): m/z calcd for $C_{54}H_{44}S_{12}$: 1076.0091 found: 1076.0004.

2a: Red powder; ^{1}H NMR ($CDCl_3$, 400 MHz) δ 2.41 (s 6H), 2.43 (s 6H), 6.40 (s, 2H), 7.06 (d, *J* = 8.4 Hz, 4H), 7.10-7.11 (m, 2H), 7.20 (d, *J* = 8.4 Hz, 4H), 7.24-7.25 (m, 2H); ^{13}C NMR ($CDCl_3$, 100 MHz) δ 19.9, 20.0, 108.5, 111.9, 114.7, 122.8, 125.4, 126.8, 127.8, 128.6, 129.4, 130.2, 130.7, 134.6, 137.2, 138.0; Mp 104−105 ^{o}C; HRMS (MALDI-TOF): m/z calcd for $C_{34}H_{26}S_{12}$: 817.8683; found: 817.8531.

2b: Red powder; ^{1}H NMR (C_6D_6-CS_2, 400 MHz) δ 1.82 (s 6H), 1.84 (s 6H), 6.18 (s, 2H), 6.92-6.96 (m, 6H), 7.03-708 (m, 6H); ^{13}C NMR (C_6D_6-CS_2, 100 MHz) δ 13.0, 13.8, 107.2, 111.4, 111.7, 121.5, 121.9, 122.0, 125.9, 126.8, 128.6, 129.1, 129.4, 136.2, 137.1, 137.8; Mp 204−205 ^{o}C; HRMS (MALDI-TOF): m/z calcd for $C_{34}H_{26}S_8$: 689.9800; found: 689.9817.

4: Red brown powder; ^{1}H NMR ($CDCl_3$, 400 MHz) δ 2.15 (s 12H), 2.21 (s, 12H), 2.27 (s, 12H), 2.31 (s, 12H), 5.79 (s, 4H), 7.10 (d, *J* = 8.0 Hz, 8H), 7.18 (d, *J* = 8.0 Hz, 8H); Mp 157−158 ^{o}C (decomposed).

Typical procedure for the synthesis of 3: Compound **10** (15.1 mg, ca. 0.0233 mmol), **11** (43.0 mg, 0.186 mmol), dry toluene (0.9 mL), and $P(OEt)_3$ (0.9 mL) were placed in a 50-mL reaction flask under an argon atmosphere. The mixture was heated at reflux for 12 h. After removal of the solvent and excess $P(OEt)_3$, the residue was purified by silica gel chromatography with dichloromethane as the eluent. The product **3** was obtained by suction filtration with methanol and hexane in 29% yield from TTF (16.7 mg).

3: Brown powder; ^{1}H NMR ($CDCl_3$, 400 MHz) δ 2.42 (s 12H), 2.44 (s 12H), 6.60 (s, 4H), 6.74 (d, J = 4.0 Hz, 4H), 7.02 (d, J = 4.0 Hz, 4H); Mp 275–276 °C. This compound is too insoluble to record a ^{13}C NMR spectrum.

Synthesis of 4 (Scheme 2b): Compound **13** (24.3 mg, 0.017 mmol), **11** (61.2 mg, 0.270 mmol), dry toluene (0.7 mL), and $P(OEt)_3$ (0.7 mL) were placed in a 50-mL reaction flask under an argon atmosphere. The mixture was heated at reflux for 12 h. After removal of the solvent and excess $P(OEt)_3$ in vacuo, the residue was precipitated from dichloromethane/hexane and washed with methanol, hexane, and acetone to give **4** in 44% yield from **1a**.

Compounds 6 and 7 were synthesized as outlined below

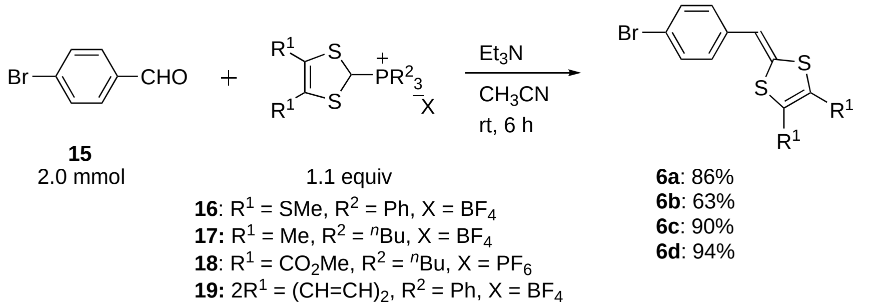

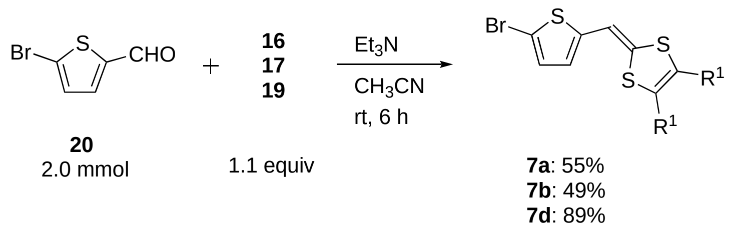

Typical procedure for the synthesis of 6 and 7: To a mixture of **15** (370.3 mg, 2.00 mmol) and **16** (1.151 g, 2.11 mmol) in dry acetonitrile (10 mL), triethylamine (2.5 mL) was added at 0 °C under an argon atmosphere, and the mixture was stirred for 6 h at room temperature. After removal of the solvent and excess triethylamine, the residue was purified by suction filtration with cold methanol. The product **6a** was obtained in 86% yield (627.9 mg, 1.73 mmol).

6a: Yellow powder; ^{1}H NMR ($CDCl_3$, 400 MHz) δ 2.42 (s 3H), 2.44 (s 3H), 6.41 (s 1H), 7.07 (d, *J* = 8.4 Hz, 2H), 7.46 (d, *J* = 8.4 Hz, 2H); ^{13}C NMR ($CDCl_3$, 100 MHz) δ 18.9, 19.0, 113.4, 119.3, 124.2, 127.0, 128.2, 131.6, 133.3, 135.1; Mp 91–92 °C; HRMS (MALDI-TOF): m/z calcd for $C_{12}H_{11}BrS_4$: 361.8927; found: 361.8902.

6b: Yellow powder; ^{1}H NMR ($CDCl_3$, 400 MHz) δ 1.95 (s 3H), 1.97 (s 3H), 6.34 (s 1H), 7.09 (d, *J* = 8.4 Hz, 2H), 7.43 (d, *J* = 8.4 Hz, 2H); ^{13}C NMR ($CDCl_3$, 100 MHz) δ 12.9, 13.6, 110.1, 118.2, 121.1, 121.2, 127.9, 131.4, 135.5 135.9; Mp 91–92 °C; HRMS (MALDI-TOF): m/z calcd for $C_{12}H_{11}BrS_2$: 297.9486; found: 297.9540.

6c: Orange powder; ^{1}H NMR ($CDCl_3$, 400 MHz) δ 3.85 (s 3H), 3.87 (s 3H), 6.39 (s 1H), 7.08 (d, *J* = 8.4 Hz, 2H), 7.32 (d, *J* = 8.4 Hz, 2H); ^{13}C NMR ($CDCl_3$, 100 MHz) δ 53.3, 53.4, 114.2, 120.0, 128.2, 129.0, 131.4, 131.6, 131.7 134.6, 159.6, 160.1; Mp 112–113 °C; HRMS (MALDI-TOF): m/z calcd for $C_{14}H_{11}BrO_4S_2$: 385.9282; found: 385.9268.

6d: White powder; ^{1}H NMR ($CDCl_3$, 400 MHz) δ 6.49 (s 1H), 7.10-7.16 (m 2H), 7.20 (d, *J* = 8.4 Hz, 2H), 7.24-7.28 (m 2H), 7.48 (d, *J* = 8.4 Hz, 2H); ^{13}C NMR ($CDCl_3$, 100 MHz) δ 106.0, 113.4, 119.6, 121.2, 122.0, 125.9, 126.3, 128.5, 131.7 134.0, 134.8, 135.6; Mp 182–183 °C; HRMS (MALDI-TOF): m/z calcd for $C_{14}H_9BrS_2$: 319.9329; found: 319.9322.

7a: Yellow powder; ^{1}H NMR ($CDCl_3$, 400 MHz) δ 2.43 (s 3H), 2.45 (s 3H), 6.56 (s 1H), 6.60 (d, *J* = 4.0 Hz, 2H), 6.97 (d, *J* = 4.0 Hz, 2H); ^{13}C NMR ($CDCl_3$, 100 MHz) δ 18.9, 19.1, 107.6, 110.7, 123.7, 125.1, 127.9, 130.1, 131.6 142.0; Mp 41–42 °C (decomposed).

7b: White powder; ^{1}H NMR (C_6D_6, 400 MHz) δ 1.93 (s 3H), 1.97 (s 3H), 6.33 (s 1H), 6.43 (d, *J* = 3.6 Hz, 2H), 6.80 (d, *J* = 3.6 Hz, 2H); ^{13}C NMR ($CDCl_3$, 100 MHz) δ 12.9, 13.6,110.1, 118.2, 121.1, 121.2, 127.9, 131.4, 135.5 135.9; Mp 61–62 °C (decomposed).

7d: White powder; ^{1}H NMR ($CDCl_3$, 400 MHz) δ 6.64 (s 1H), 6.68 (d , *J* = 4.0 Hz, 1H), 6.99 (d , *J* = 4.0 Hz, 1H), 7.12-7.17 (m, 2H), 7.23-7.25 (m, 1H) , 7.28-7.32 (m, 1H); ^{13}C NMR ($CDCl_3$, 100 MHz) δ 107.4, 110.8, 121.2, 122.0, 124.0, 125.7, 126.2, 130.0, 132.0, 135.2, 136.2, 142.2; Mp 68–69 °C (decomposed).

Synthesis of 9: $Pd(OAc)_2$ (25.5 mg, 0.113 mmol), P*t*-Bu_3·HBF_4 (98.5 mg, 0.340 mmol), and Cs_2CO_3 (737.7 mg, 2.263 mmol) were placed in a 30-mL reaction flask under argon. 1,4-Dioxane (2mL) was added and the mixture was stirred for 10 min at 50 °C. A solution of compound **8** (516.2 mg, 1.947 mmol) in 1,4-dioxane (2 mL) and TTF (38.4 mg, 0.188 mmol) were added. The mixture was heated at 110 °C for 72 h. The obtained solid was washed with dichloromethane and methanol to yield **9** as dark brown oil. Being identified by ^{1}H NMR, this compound was used for the next step without further purification.

Synthesis of 10: To a mixture of **9** (300.8 mg) in DMF (10 mL) and distilled water (10 mL) PTSA·H_2O (244.0 mg, 0.602 mmol) was added at room temperature, and the mixture was stirred for 1 h. The organic compounds were extracted with dichloromethane three times. The combined organic layer was washed with H_2O, sat. $NaHCO_3$ aq., and brine, dried over anhydrous Na_2SO_4, and concentrated in vacuo. The obtained solid was washed with methanol and hexane to yield **10** as black solid. Being identified by ^{1}H NMR, this compound was used for the next step without further purification.

Compound 12 was synthesized as outlined below

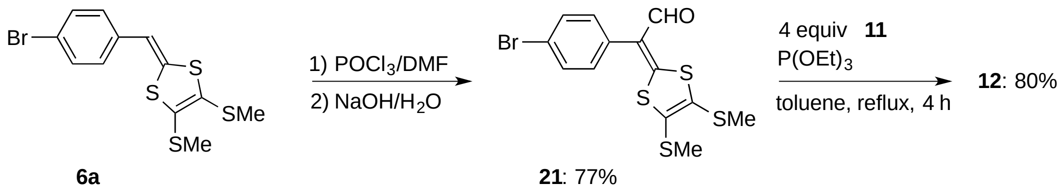

Synthesis of 12: Compound **21** (196.1 mg, 0.501 mmol), **11** (456.0 mg, 2.01 mmol), dry toluene (2.4 mL), and $P(OEt)_3$ (2.4 mL) were placed in a 50-mL reaction flask under an argon atmosphere. The mixture was heated at reflux for 4 h. After removal of the solvent and excess $P(OEt)_3$ in vacuo, the residue was precipitated from dichrolomethane/hexane and washed with methanol to give **12** in 80% yield.

Yellow powder; ^{1}H NMR ($CDCl_3$, 400 MHz) δ 2.21 (s 3H), 2.32 (s 3H), 2.37 (s 3H), 2.43 (s, 3H), 5.95 (s, 1H) , 7.15 (d , J = 8.4 Hz, 2H) 7.52 (d , J = 8.4 Hz, 2H); ^{13}C NMR ($CDCl_3$, 100 MHz) δ 18.8, 18.8 (2C), 18.9, 111.7, 122.1, 123.2, 126.0, 126.2, 126.5, 127.0, 131.1, 131.2, 132.3, 132.7, 135.5; Mp 133–134 °C; HRMS (MALDI-TOF): m/z calcd for $C_{18}H_{17}BrS_8$: 567.8279; found: 567.8280.

Synthesis of 13: To a mixture of **1a** (33.0 mg, 0.025 mmol) in DMF (10 mL) $POCl_3$ (74 µL, 0.79 mmol) was added at 0 °C, and the mixture was warmed to room temperature and stirred for 2 h. Afterwards, 1 M NaOH aq. (1.2 mL) was added at 0 °C and the mixture was warmed to room temperature and stirred for 30 min. The mixture was extracted with dichloromethane. The combined organic layers were washed with H_2O and brine three times, dried over anhydrous Na_2SO_4, and concentrated in vacuo. The product was obtained by suction filtration with dichloromethane and hexane in 44% yield from **1a**. Being identified by ^{1}H NMR, this compound was used for the next step without further purification.

Synthesis of 21: To compound **6a** (1.150 g, 3.16 mmol) in DMF (20 mL) $POCl_3$ (1.2 mL, 12.7 mmol) was added at 0 °C and the mixture was warmed to room temperature and stirred for 2 h. After the reaction, 1 M NaOH aq. (38 mL) was added at 0 °C and the mixture was warmed to room temperature, and stirred for 30 min. The mixture was extracted with dichlorometane. The combined organic layers were washed with H_2O and brine three times,

dried over anhydrous Na_2SO_4, and concentrated in vacuo. The product was obtained by suction filtration with dichlorometane and hexane in 77% yield.

21: Yellow powder; ^{1}H NMR ($CDCl_3$, 400 MHz) δ 2.41 (s 3H), 2.56 (s 3H), 7.27 (d , *J* = 8.0 Hz, 2H), 7.60 (d , *J* = 8.0 Hz, 2H), 9.31 (s, 1H); ^{13}C NMR ($CDCl_3$, 100 MHz) δ 18.8, 19.2, 121.3, 122.3, 125.8, 130.2, 132.5, 133.6, 135.5, 160.8, 183.2; Mp 158−159 °C; HRMS (MALDI-TOF): m/z calcd for $C_{13}H_{11}BrOS_4$: 389.8876; found: 389.8856.

Theoretical calculations

The theoretical calculations of the compounds **1a**, **3a**, and **4**, and their pristine compounds were carried out based on density functional theory (DFT) using the spin-restricted B3LYP/6-31G(d,p) level of theory. The compounds can adopt a number of conformations. The calculations of 14 conformations for the pristine compounds of **1** and **4** and 42 conformations for the pristine compound of **3** were performed and the predominant conformations were determined. The calculations of 14 conformations of **1a** based on the conformations of pristine **1** were also carried out and we confirmed that a lowest-energy conformation was obtained by the same model of pristine **1**. Since the calculations of **3a** and **4** were expected to require high computational costs, several conformations of **3a** and **4**, based on the lower-energy conformation of the pristine compound, were calculated. Frequency calculations were performed to confirm that these structures were indeed local minima. The Gaussian 16 software [1] was used for all calculations.

References

[1] Gaussian 16, *Revision C.01,* Gaussian, Inc.: Wallingford CT, 2019.

a) **3a**

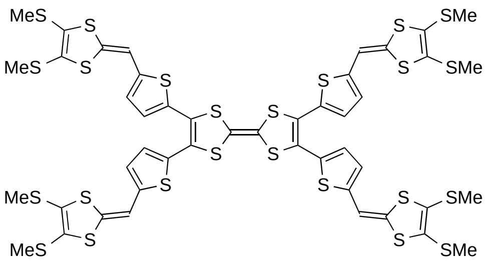

b) **4**

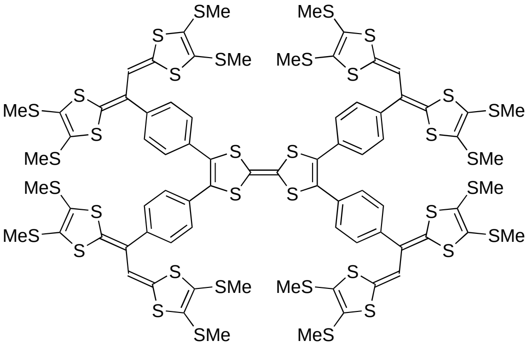

LUMO: –1.75 eV

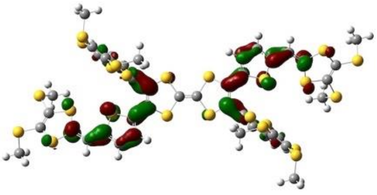

LUMO: –1.72 eV

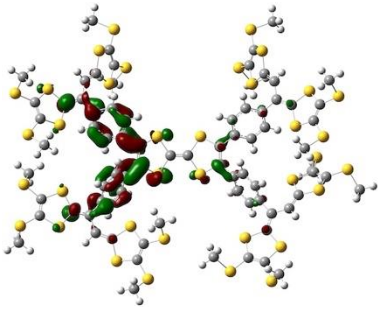

HOMO: –4.49 eV

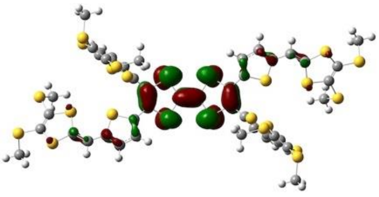

HOMO: –4.49 eV

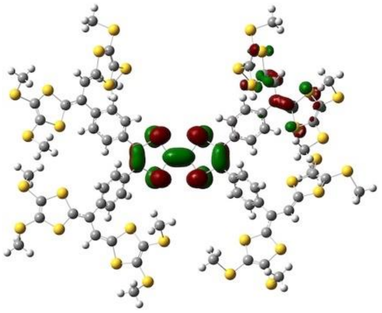

HOMO–1: –4.87 eV

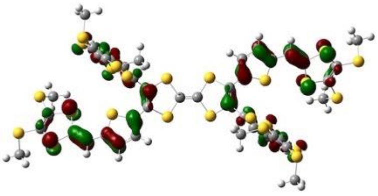

HOMO–1: –4.62 eV

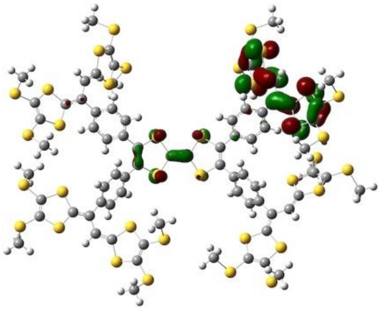

Figure S1: a) Molecular orbitals of **3a** and b) **4**.

Dihedral angles

Table S1: Dihedral angles [°] of **1a**, **3a**, and **4**

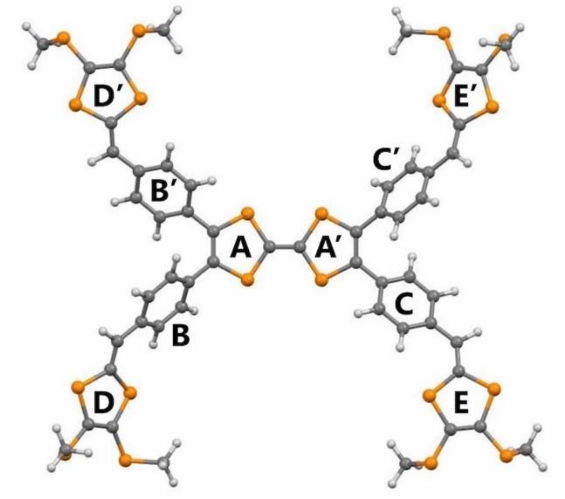

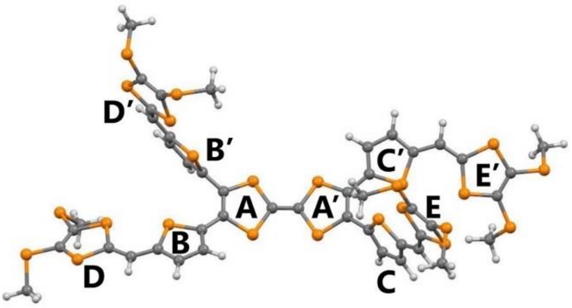

**1a**		**3a**	
A–A’	158.0	A–A’	153.0
A–B	49.8	A–B	20.2
A–B’	137.7	A–B’	90.8
A’–C	137.7	A’–C	104.5
A’–C’	49.9	A’–C’	26.6
B–D	28.6	B–D	12.7
B’–D’	28.5	B’–D’	12.4
C–E	28.5	C–E	12.4
C’–E’	28.6	C’–E’	12.6
A–D	153.7	A–D	152.7
A–D’	18.1	A–D’	77.7
A’–E	18.1	A’–E	84.9
A’–E’	153.6	A’–E’	140.8

Table S1: *Continued*

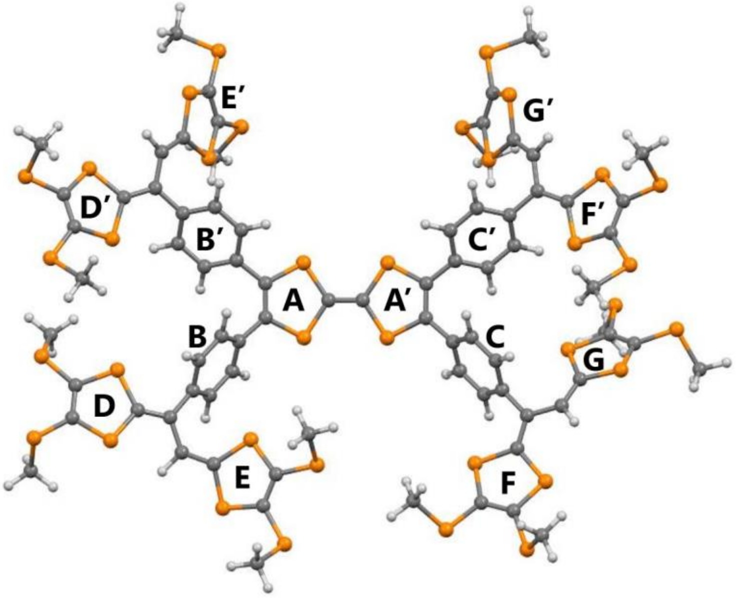

4

A’–A	153.3		
A–B	49.4	A–B’	138.6
A’–C	49.8	A’–C’	141.2
B–D	112.7	B’–D’	116.9
C–F	110.3	C’–F’	113.0
D–E	32.5	D’–E’	33.8
F–G	29.1	F’–G’	30.8
A–D	18.8	A–D’	21.7
A’–F	23.5	A’–F’	28.5
B–E	95.9	B’–E’	100.6
C–G	95.9	C’–G’	98.6
A–E	144.8	A–E’	134.3
A’–G	130.0	A’–G’	129.5

Optimized geometry

Pristine compound of **1**

Energy data for pristine compound of **1**

	E [hartree]	ZP*E* [hartree]	*E* + ZP*E* [hartree]	Δ*E* [kcal/mol]	Δ(*E* + ZP*E*) [kcal/mol]
model-1	-6547.910164	0.596811	-6547.313353	0.060868	0.062123
model-2	-6547.910070	0.596828	-6547.313242	0.119854	0.131777
model-3	-6547.910116	0.596827	-6547.313289	0.090989	0.102284
model-4	-6547.910095	0.596798	-6547.313297	0.104167	0.097264
model-5	-6547.910174	0.596847	-6547.313327	0.054593	0.078439
model-6	-6547.910179	0.596839	-6547.313340	0.051456	0.070281
model-7	-6547.910154	0.596814	-6547.313340	0.067144	0.070281
model-8	-6547.910216	0.596764	**-6547.313452**	0.028238	**0**
model-9	-6547.910167	0.596848	-6547.313319	0.058986	0.083459
model-10	-6547.910214	0.596840	-6547.313374	0.029493	0.048946
model-11	-6547.910195	0.596802	-6547.313393	0.041416	0.037023
model-12	-6547.910255	0.596840	-6547.313415	0.003765	0.023218
model-13	**-6547.910261**	0.596832	-6547.313429	**0**	0.014433
model-14	-6547.910239	0.596800	-6547.313439	0.013805	0.008158

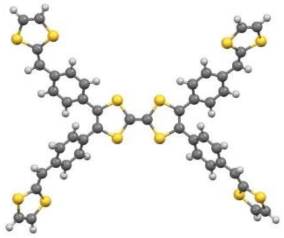

model-1
Δ(*E* + ZP*E*) = 0.062123 kcal/mol

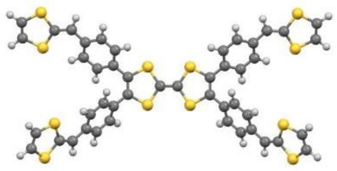

model-2
Δ(*E* + ZP*E*) = 0.131777 kcal/mol

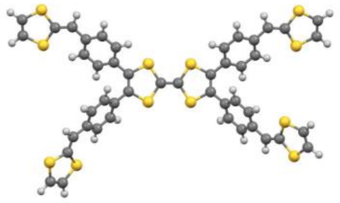

model-3
Δ(*E* + ZP*E*) = 0.102284 kcal/mol

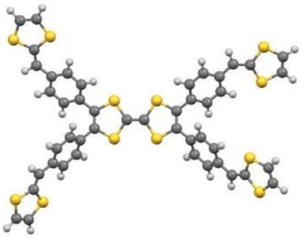

model-4
Δ(*E* + ZP*E*) = 0.097264 kcal/mol

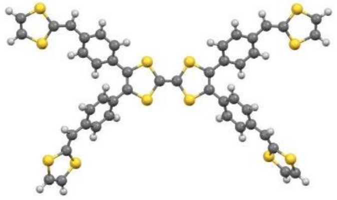

model-5
Δ(*E* + ZP*E*) = 0.078439 kcal/mol

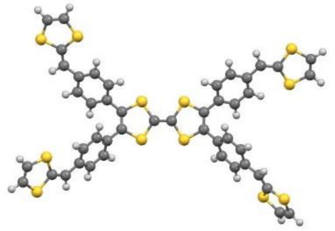

model-6
Δ(*E* + ZP*E*) = 0.070281 kcal/mol

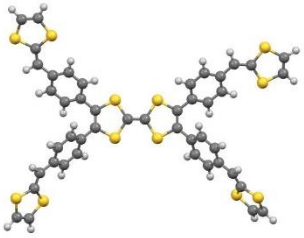

model-7
Δ(*E* + ZP*E*) = 0.070281 kcal/mol

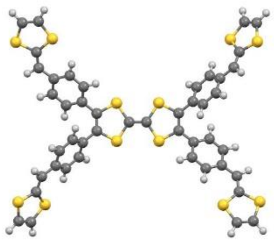

model-8
Δ(*E* + ZP*E*) = 0 kcal/mol

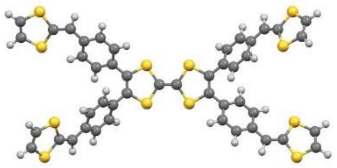

model-9
Δ(*E* + ZP*E*) = 0.083459 kcal/mol

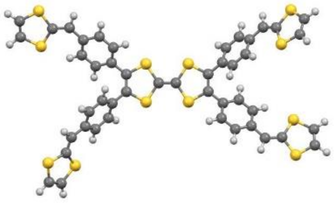

model-10
Δ(E + ZPE) = 0.048946 kcal/mol

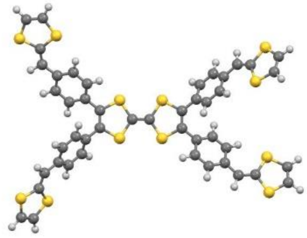

model-11
Δ(E + ZPE) = 0.037023 kcal/mol

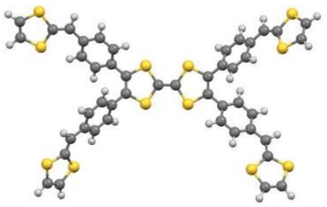

model-12
Δ(E + ZPE) = 0.023218 kcal/mol

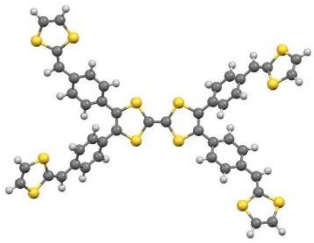

model-13
Δ(E + ZPE) = 0.014433 kcal/mol

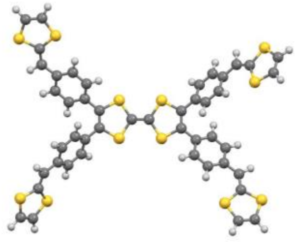

model-14
Δ(E + ZPE) = 0.008158 kcal/mol

Compound **1a**

Energy data for compound **1a**

	E [hartree]	ZP*E* [hartree]	*E* + ZP*E* [hartree]	Δ*E* [kcal/mol]	Δ(*E* + ZP*E*) [kcal/mol]
model-1	-10047.929607	0.826758	-10047.102849	0.331325	0.328187
model-2	-10047.929485	0.826703	-10047.102782	0.407881	0.370231
model-3	-10047.929668	0.826734	-10047.102934	0.293047	0.274849
model-4	-10047.929602	0.826712	-10047.102890	0.334463	0.302460
model-5	-10047.929803	0.826737	-10047.103066	0.208333	0.192018
model-6	-10047.929951	0.826762	-10047.103189	0.115462	0.114834
model-7	-10047.929756	0.826761	-10047.102995	0.237826	0.236571
model-8	**-10047.930135**	0.826763	**-10047.103372**	**0**	**0**
model-9	-10047.929555	0.826708	-10047.102847	0.284889	0.288654
model-10	-10047.929718	0.826719	-10047.102999	0.363956	0.329443
model-11	-10047.929870	0.826754	-10047.103116	0.261671	0.234061
model-12	-10047.929867	0.826762	-10047.103105	0.166290	0.160642
model-13	-10047.929881	0.826732	-10047.103149	0.168173	0.167545
model-14	-10047.930047	0.826764	-10047.103283	0.159387	0.139935

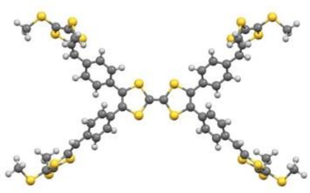

model-1
Δ(*E* + ZP*E*) = 0.328187 kcal/mol

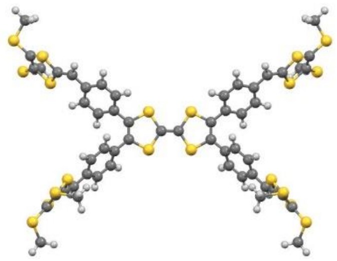

model-2
Δ(*E* + ZP*E*) = 0.370231 kcal/mol

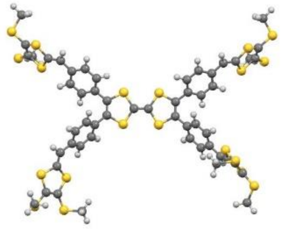

model-3
Δ(*E* + ZP*E*) = 0.274849 kcal/mol

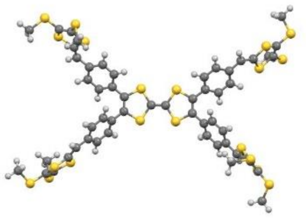

model-4
Δ(*E* + ZP*E*) = 0.302460 kcal/mol

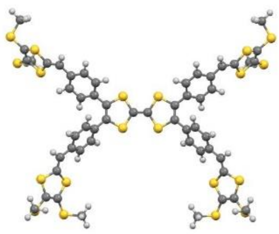

model-5
Δ(*E* + ZP*E*) = 0.192018 kcal/mol

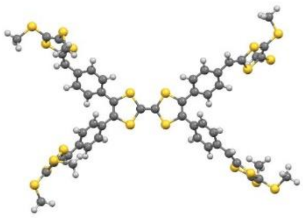

model-6
Δ(*E* + ZP*E*) = 0.114834 kcal/mol

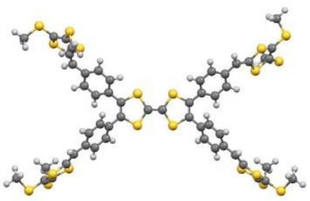

model-7
Δ(*E* + ZP*E*) = 0.236571 kcal/mol

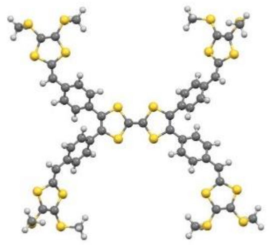

model-8
Δ(*E* + ZP*E*) = 0 kcal/mol

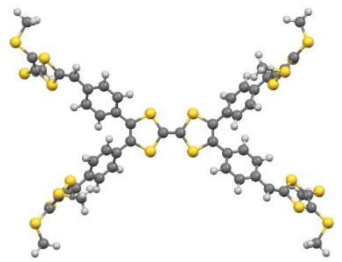

model-9
Δ(*E* + ZP*E*) = 0.288654 kcal/mol

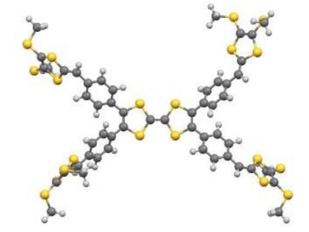

model-10
Δ(E + ZPE) = 0.329443 kcal/mol

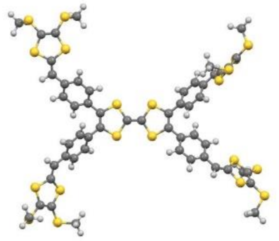

model-11
Δ(E + ZPE) = 0.234061 kcal/mol

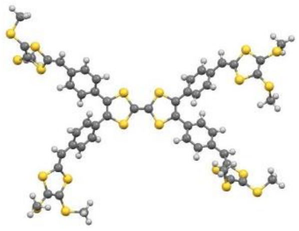

model-12
Δ(E + ZPE) = 0.160642 kcal/mol

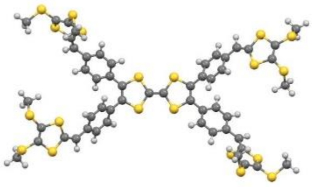

model-13
Δ(E + ZPE) = 0.167545 kcal/mol

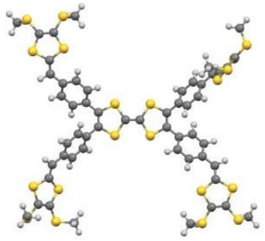

model-14
Δ(E + ZPE) = 0.139935 kcal/mol

Pristine compound of **3**

Energy data for pristine compound of **3**

	E [hartree]	Z PE [hartree]	E + ZPE [hartree]	ΔE [kcal/mol]	Δ(E + ZPE) [kcal/mol]
model-1	-7830.927826	0.461865	-7830.465961	0.338372	0.279386
model-2	-7830.925824	0.462149	-7830.463675	1.594665	1.713892
model-3	-7830.926346	0.462078	-7830.464268	1.266634	1.341308
model-4	-7830.926905	0.462016	-7830.464889	0.916333	0.952101
model-5	-7830.926839	0.462012	-7830.464827	0.957724	0.990982
model-6	-7830.926931	0.462019	-7830.464912	0.899736	0.937386
model-7	-7830.927355	0.461943	-7830.465412	0.633741	0.623701
model-8	-7830.928058	0.461865	-7830.466193	0.192645	0.133660
model-9	-7830.925844	0.462154	-7830.463690	1.581952	1.704316
model-10	-7830.926448	0.462092	-7830.464356	1.202936	1.286395
model-11	-7830.926950	0.462009	-7830.464941	0.887926	0.919301
model-12	-7830.926973	0.462011	-7830.464962	0.873493	0.906124
model-13	-7830.927056	0.462028	-7830.465028	0.821410	0.864708
model-14	-7830.927558	0.461945	-7830.465613	0.506400	0.497615
model-15	-7830.928220	0.461918	-7830.466302	0.090989	0.065261
model-16	-7830.926231	0.462186	-7830.464045	1.339105	1.481550
model-17	-7830.926752	0.462110	-7830.464642	1.012173	1.106927
model-18	-7830.927342	0.462051	-7830.465291	0.641942	0.699673
model-19	-7830.927143	0.462064	-7830.465079	0.766817	0.832705
model-20	-7830.927335	0.462075	-7830.465260	0.646335	0.719126
model-21	-7830.927917	0.462011	-7830.465906	0.281124	0.313755
model-22	-7830.928253	0.461962	-7830.466291	0.070281	0.072164
model-23	-7830.926518	0.462218	-7830.464300	1.159010	1.321535
model-24	-7830.926901	0.462154	-7830.464747	0.918674	1.041038
model-25	-7830.927395	0.462081	-7830.465314	0.608684	0.685240
model-26	-7830.927278	0.462105	-7830.465173	0.611822	0.701556
model-27	-7830.927480	0.462114	-7830.465366	0.555346	0.652610
model-28	-7830.927952	0.462044	-7830.465908	0.259161	0.312500
model-29	**-7830.928365**	0.461959	**-7830.466406**	**0**	**0**
model-30	-7830.926614	0.462215	-7830.464399	1.098769	1.259412
model-31	-7830.927204	0.462175	-7830.465029	0.728539	0.864081
model-32	-7830.927504	0.462089	-7830.465415	0.540286	0.621862
model-33	-7830.927586	0.462135	-7830.465451	0.488830	0.599272
model-34	-7830.927771	0.462139	-7830.465632	0.372741	0.485692
model-35	-7830.928066	0.462053	-7830.466013	0.187625	0.246611
model-36	-7830.928134	0.461924	-7830.466210	0.144955	0.122992
model-37	-7830.926193	0.462186	-7830.464007	1.362951	1.505395
model-38	-7830.926755	0.462115	-7830.464640	1.010290	1.108182
model-39	-7830.927247	0.462034	-7830.465213	0.701556	0.748619
model-40	-7830.927147	0.462056	-7830.465091	0.764307	0.825175
model-41	-7830.927325	0.462077	-7830.465248	0.652610	0.726656
model-42	-7830.927828	0.462001	-7830.465827	0.336973	0.363328

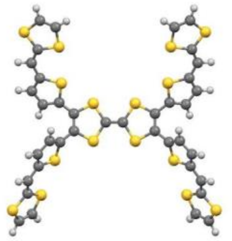

model-1

$\Delta(E + \mathrm{ZP}E)$ = 0.279386 kcal/mol

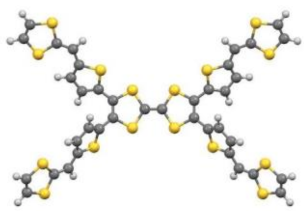

model-2

$\Delta(E + \mathrm{ZP}E)$ = 1.713892 kcal/mol

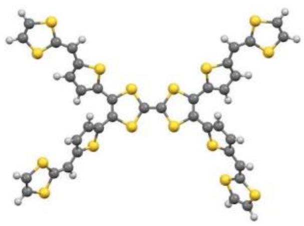

model-3

$\Delta(E + \mathrm{ZP}E)$ = 1.341308 kcal/mol

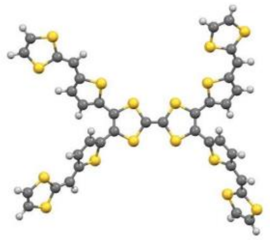

model-4

$\Delta(E + \mathrm{ZP}E)$ = 0.952101 kcal/mol

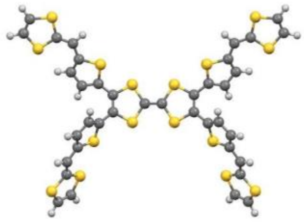

model-5

$\Delta(E + \mathrm{ZP}E)$ = 0.990982 kcal/mol

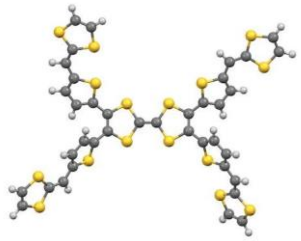

model-6

$\Delta(E + \mathrm{ZP}E)$ = 0.937386 kcal/mol

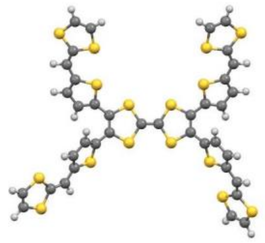

model-7

$\Delta(E + \mathrm{ZP}E)$ = 0.623701 kcal/mol

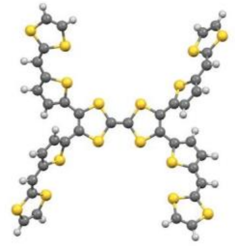

model-8

$\Delta(E + \mathrm{ZP}E)$ = 0.133660 kcal/mol

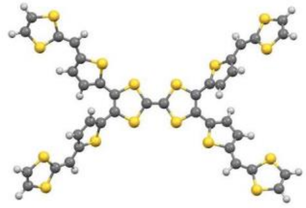

model-9

$\Delta(E + \mathrm{ZP}E)$ = 1.704316 kcal/mol

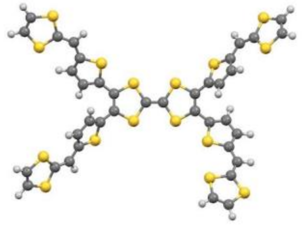

model-10

$\Delta(E + \mathrm{ZP}E)$ = 1.286395 kcal/mol

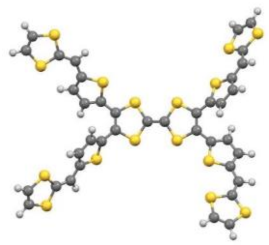

model-11

$\Delta(E + \mathrm{ZP}E)$ = 0.919301 kcal/mol

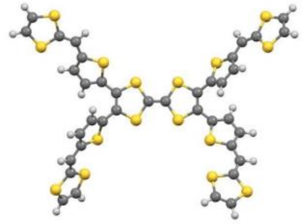

model-12

$\Delta(E + \mathrm{ZP}E)$ = 0.906124 kcal/mol

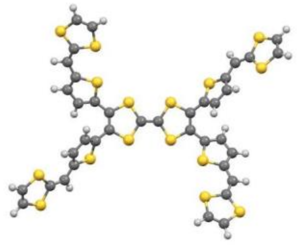

model-13

$\Delta(E + \mathrm{ZP}E)$ = 0.864708 kcal/mol

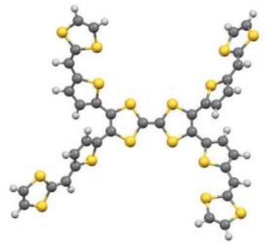

model-14

$\Delta(E + \mathrm{ZP}E)$ = 0.497615 kcal/mol

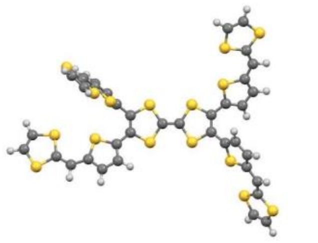

model-15

$\Delta(E + \mathrm{ZP}E)$ = 0.065261 kcal/mol

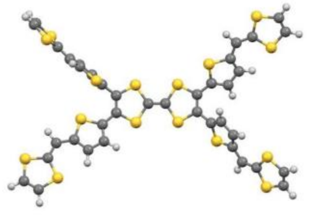

model-16
$\Delta(E + ZPE)$ = 1.481550 kcal/mol

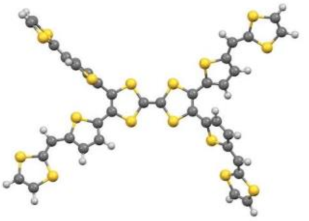

model-17
$\Delta(E + ZPE)$ = 1.106927 kcal/mol

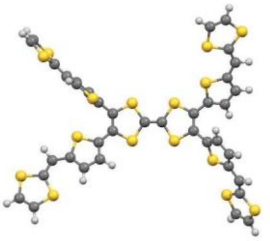

model-18
$\Delta(E + ZPE)$ = 0.699673 kcal/mol

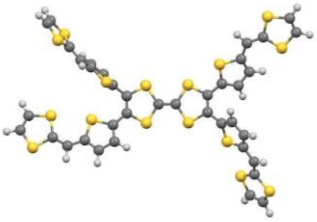

model-19
$\Delta(E + ZPE)$ = 0.832705 kcal/mol

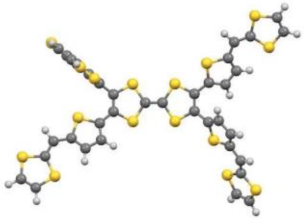

model-20
$\Delta(E + ZPE)$ = 0.719126 kcal/mol

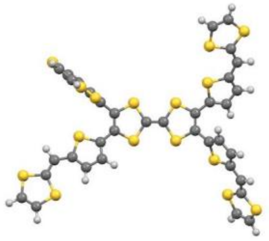

model-21
$\Delta(E + ZPE)$ = 0.313755 kcal/mol

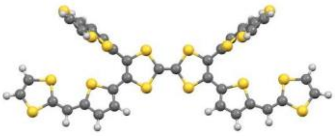

model-22
$\Delta(E + ZPE)$ = 0.072164 kcal/mol

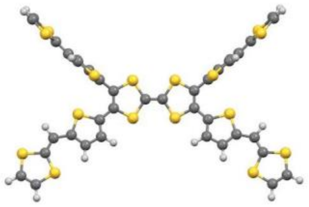

model-23
$\Delta(E + ZPE)$ = 1.321535 kcal/mol

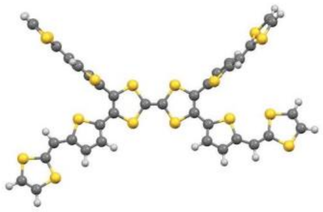

model-24
$\Delta(E + ZPE)$ = 1.041038 kcal/mol

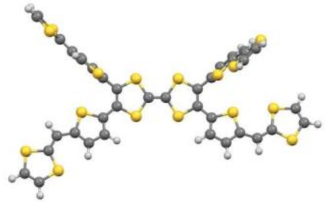

model-25
$\Delta(E + ZPE)$ = 0.685240 kcal/mol

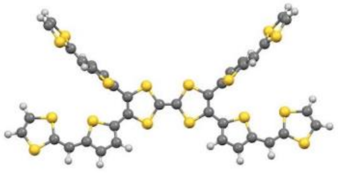

model-26
$\Delta(E + ZPE)$ = 0.701556 kcal/mol

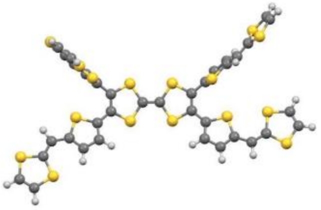

model-27
$\Delta(E + ZPE)$ = 0.652610 kcal/mol

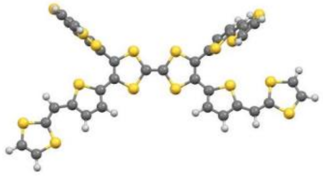

model-28
$\Delta(E + ZPE)$ = 0.312500 kcal/mol

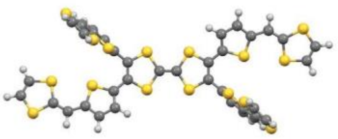

model-29
$\Delta(E + ZPE)$ = 0 kcal/mol

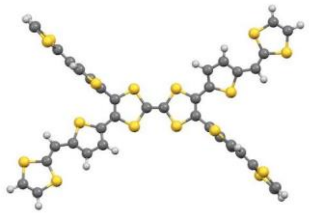

model-30
$\Delta(E + ZPE)$ = 1.259412 kcal/mol

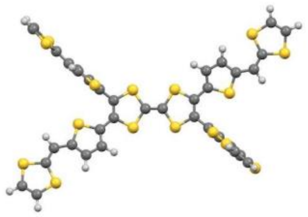

model-31
Δ(E + ZPE) = 0.864081 kcal/mol

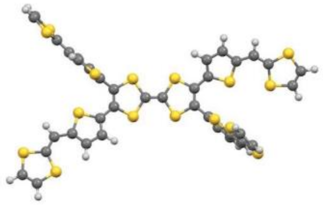

model-32
Δ(E + ZPE) = 0.621862 kcal/mol

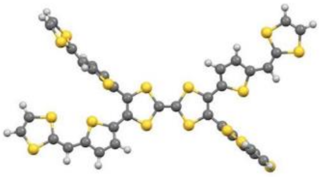

model-33
Δ(E + ZPE) = 0.599272 kcal/mol

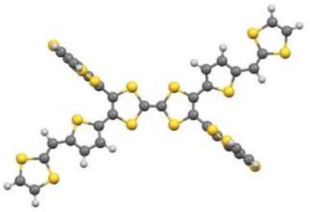

model-34
Δ(E + ZPE) = 0.485692 kcal/mol

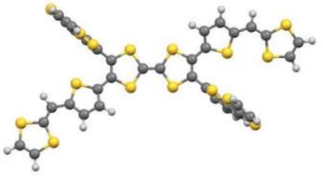

model-35
Δ(E + ZPE) = 0.246611 kcal/mol

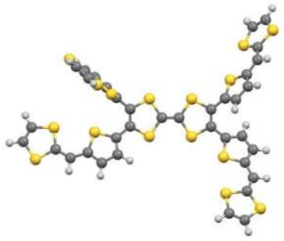

model-36
Δ(E + ZPE) = 0.122992 kcal/mol

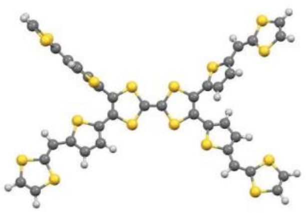

model-37
Δ(E + ZPE) = 1.505395 kcal/mol

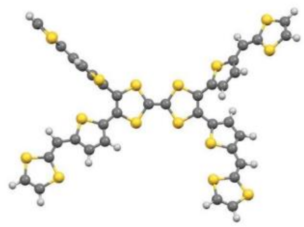

model-38
Δ(E + ZPE) = 1.108182 kcal/mol

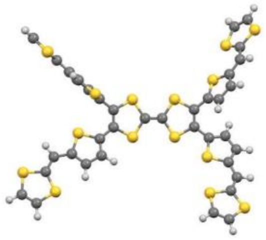

model-39
Δ(E + ZPE) = 0.748619 kcal/mol

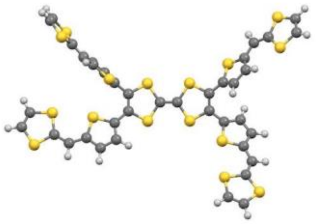

model-40
Δ(E + ZPE) = 0.825175 kcal/mol

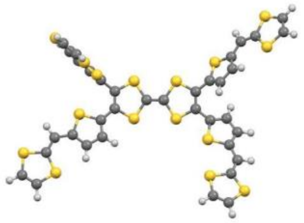

model-41
Δ(E + ZPE) = 0.726656 kcal/mol

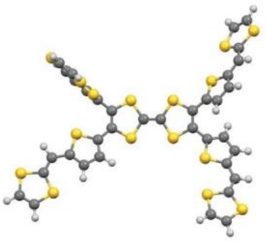

model-42
Δ(E + ZPE) = 0.008158 kcal/mol

Compound **3a**

Energy data for compound **3a**

	E [hartree]	ZP*E* [hartree]	*E* + ZP*E* [hartree]	Δ*E* [kcal/mol]	Δ(*E* + ZP*E*) [kcal/mol]
model-15	-11330.947362	0.691799	-11330.255563	0.122992	0.080949
model-22	-11330.947418	0.691850	-11330.255568	0.087851	0.077811
model-29	**-11330.947558**	0.691866	**-11330.255692**	**0**	**0**

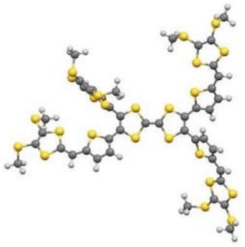

model-15
Δ(*E* + ZP*E*) = 0.080949 kcal/mol

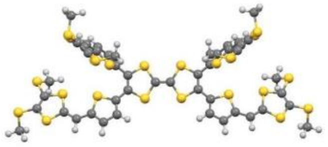

model-22
Δ(*E* + ZP*E*) = 0.077811 kcal/mol

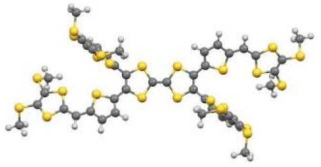

model-29
Δ(*E* + ZP*E*) = 0 kcal/mol

Pristine compound of **4**

Energy data for pristine compound of **4**

	E [hartree]	ZPE [hartree]	E + ZPE [hartree]	ΔE [kcal/mol]	Δ(E + ZPE) [kcal/mol]
model-1	-10347.811035	0.787141	-10347.023894	0.078439	0.086596
model-2	**-10347.811160**	0.787146	-10347.024014	**0**	0.011295
model-3	-10347.810675	0.787078	-10347.023597	0.304342	0.272967
model-4	-10347.810067	0.787022	-10347.023045	0.685868	0.619352
model-5	-10347.811154	0.787122	**-10347.024032**	0.003765	**0**
model-6	-10347.810643	0.787061	-10347.023582	0.324422	0.282379
model-7	-10347.811128	0.787106	-10347.024022	0.020080	0.006275
model-8	-10347.811079	0.787124	-10347.023955	0.050828	0.048318
model-9	-10347.810858	0.787093	-10347.023765	0.189508	0.167545
model-10	-10347.810333	0.787032	-10347.023301	0.518950	0.458709
model-11	-10347.810658	0.787053	-10347.023605	0.315010	0.267947
model-12	-10347.811041	0.787118	-10347.023923	0.074674	0.068399
model-13	-10347.810513	0.787038	-10347.023475	0.405999	0.349523
model-14	-10347.811096	0.787121	-10347.023975	0.040161	0.035768

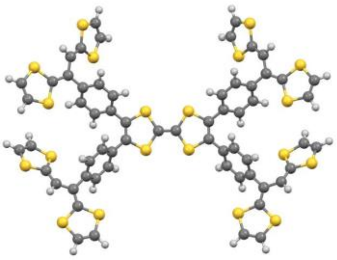

model-1
Δ(E + ZPE) = 0.086596 kcal/mol

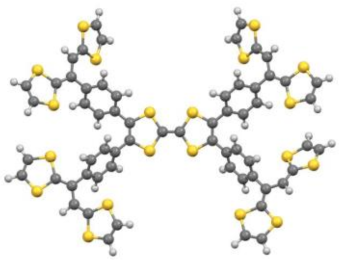

model-2
Δ(E + ZPE) = 0.011295 kcal/mol

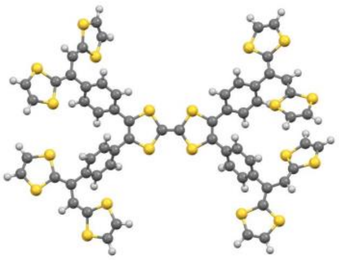

model-3
Δ(E + ZPE) = 0.272967 kcal/mol

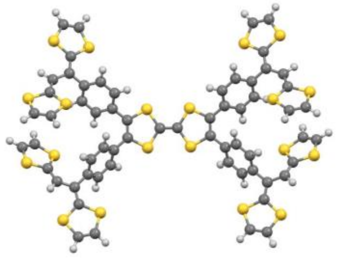

model-4
Δ(E + ZPE) = 0.619352 kcal/mol

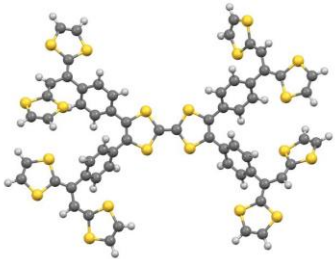

model-5
Δ(E + ZPE) = 0 kcal/mol

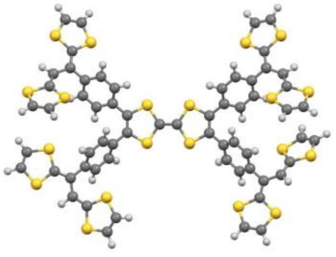

model-6
Δ(E + ZPE) = 0.282379 kcal/mol

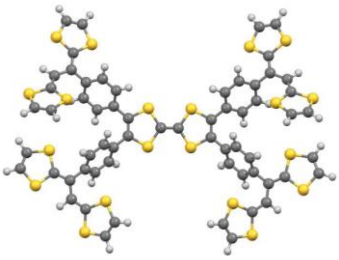

model-7
Δ(E + ZPE) = 0.006275 kcal/mol

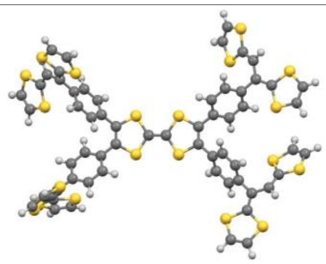

model-8
Δ(E + ZPE) = 0.048318 kcal/mol

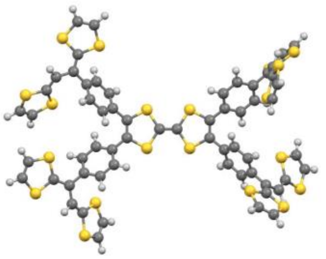

model-9
Δ(E + ZPE) = 0.167545 kcal/mol

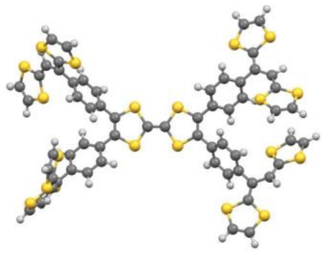

model-10
Δ(E + ZPE) = 0.458709 kcal/mol

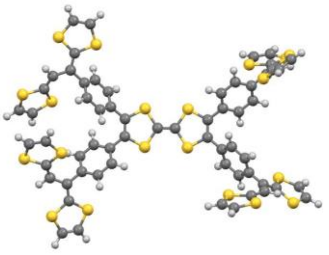

model-11
Δ(E + ZPE) = 0.267947 kcal/mol

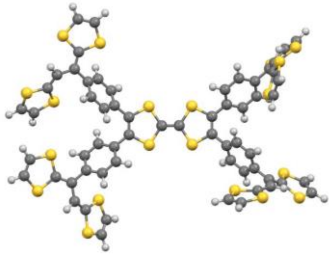

model-12
Δ(E + ZPE) = 0.068399 kcal/mol

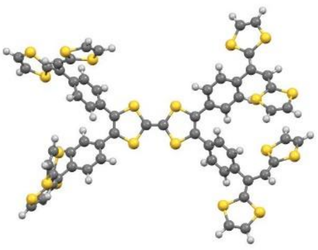

model-13
Δ(E + ZPE) = 0.349523 kcal/mol

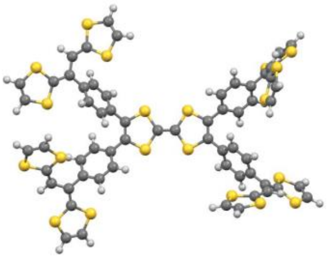

model-14
Δ(E + ZPE) = 0.035768 kcal/mol

Compound of **4**

Energy data for compound **4**

	E [hartree]	ZPE [hartree]	E + ZPE [hartree]	ΔE [kcal/mol]	Δ(E + ZPE) [kcal/mo l]
model-2	**-17347.853473**	1.247282	**-17346.606191**	**0**	**0**
model-5	-17347.853193	1.247262	-17346.605931	0.175703	0.163152

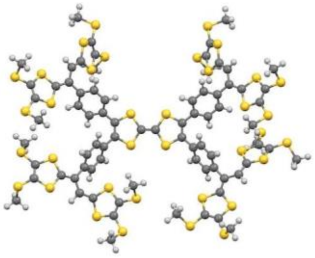

model-2

Δ(E + ZPE) = 0 kcal/mol

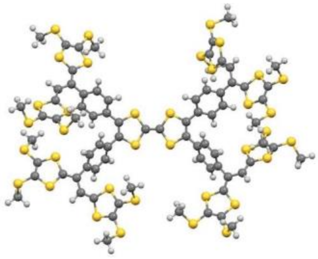

model-5

Δ(E + ZPE) = 0.163152 kcal/mol

Cyclic voltammograms

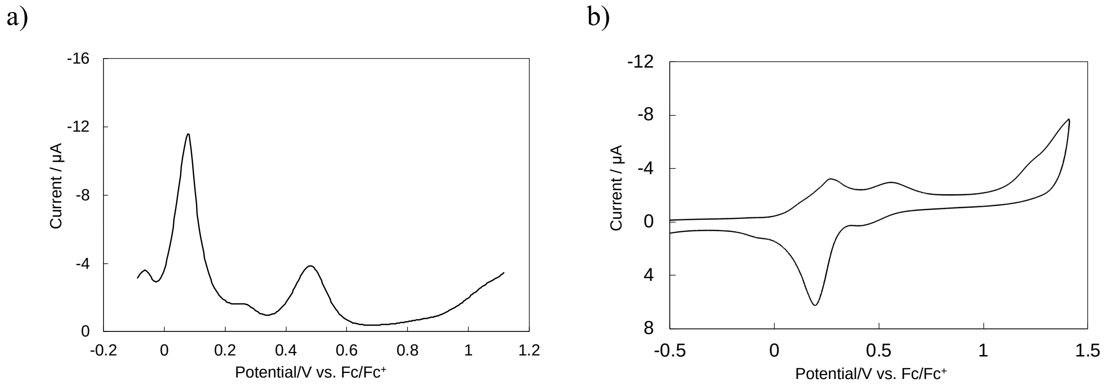

Figure S2: a) Differential pulse voltammetry of **2a** and b) cyclic voltammogram of **3a** in PhCN/CS_2 1:1 (v/v, 0.3 mM) solution in the designated solvent containing 0.1 M *n*-Bu_4NPF_6.

Table S2: Redox potentials of compounds **2a** and **3a**, and related compounds[a].

	E_1/V	E_2/V	E_3/V	E_4/V	E_5/V	E_6/V
**2a**[b]	–0.05	+0.08		+0.48		
**3a**	+0.14		+0.25			+0.52
**10**[c]	+0.25	+0.60				
**22**[c]	+0.00	+0.47				

[a]In PhCN/CS_2 1:1(v/v) containing 0.1 M *n*-Bu_4NPF_6; all potentials were measured against Ag/Ag^+ as the reference electrode and converted to vs Fc/Fc^+. [b]Differential pulse voltammetry. [c]In PhCN containing 0.1 M *n*-Bu_4NPF_6, all potentials were measured against Ag/Ag^+ as the reference electrode and converted to vs Fc/Fc^+.

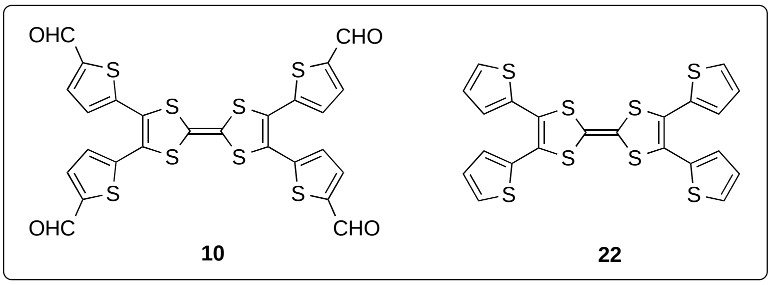

Results of the digital simulations of 1a and 4

a) **1a**

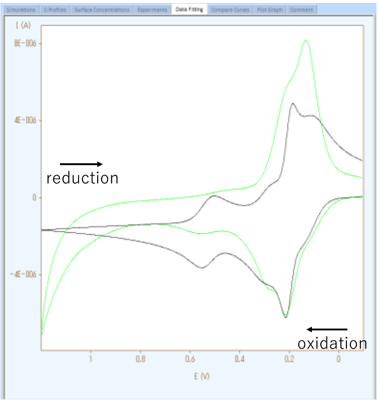

b) **4**

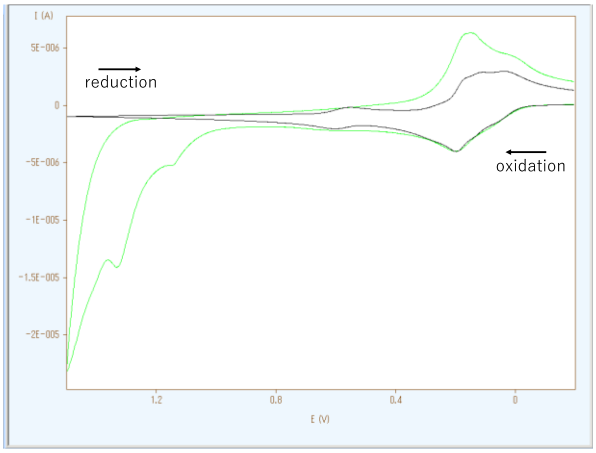

Figure S3: The results of the digital simulations of a) **1a** and b) **4**. Black line: digital simulated wave. Green line: observed wave.

Table S3: The following charge-transfer reaction and redox potentials were used for the digital simulations of **1a** and **4**.

a) **1a**

charge-transfer reaction	redox potentials (V)
A + e = B	0.502
B + e = C1	0.285
C + 2e = D1	0.2
D1 + e = D2	0.15
D2 + e = D3	0.1

b) **4**

charge-transfer reaction	redox potentials (V)
A + e = B	0.582
B + e = C1	0.26
C1 + e = C2	0.19
C2 + e = C3	0.184
C3 + e = C4	0.18
C4 + e = D	0.133
D + e = E1	0.129
E1 + e = E2	0.088
E2 + e = E3	0.05
E3 + e = F	0.02

^{1}H NMR of **1a**

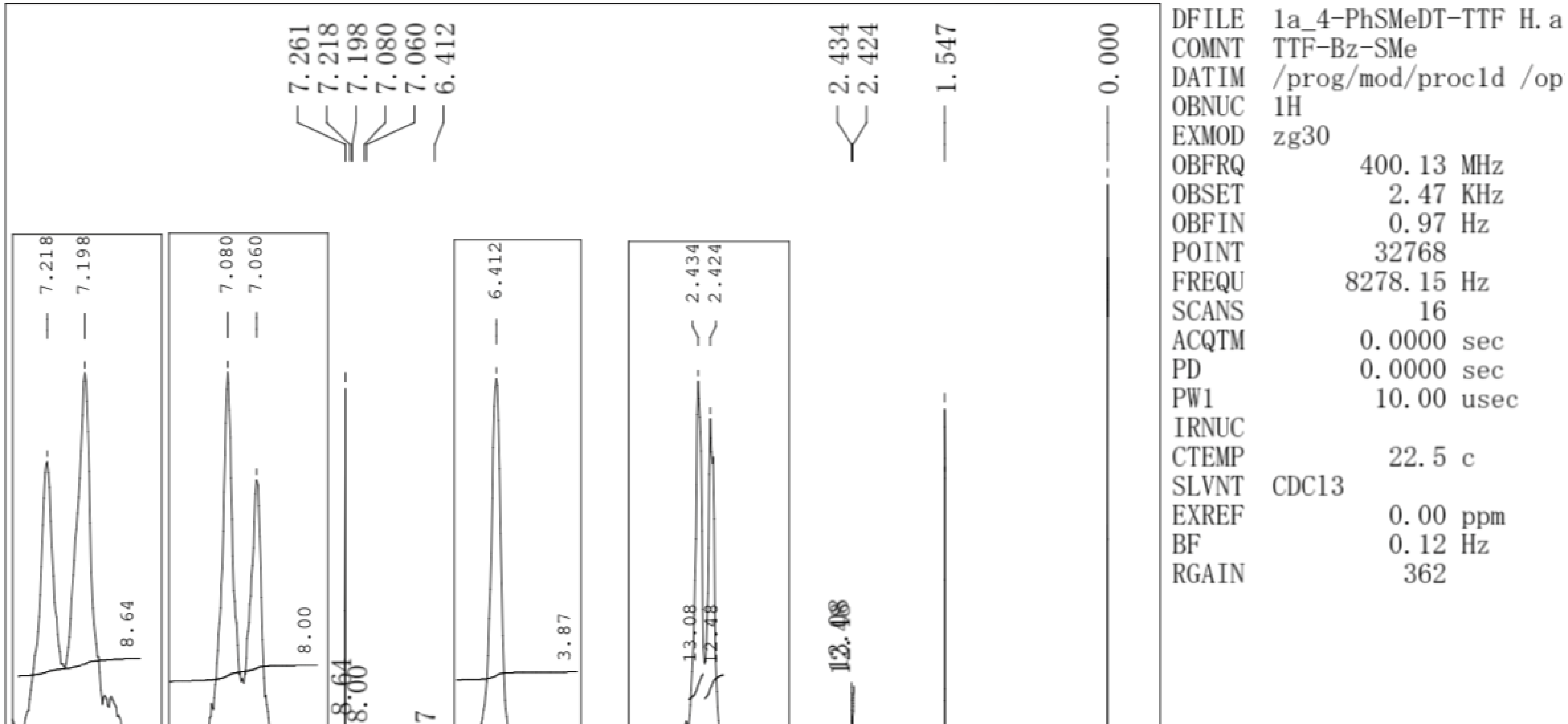

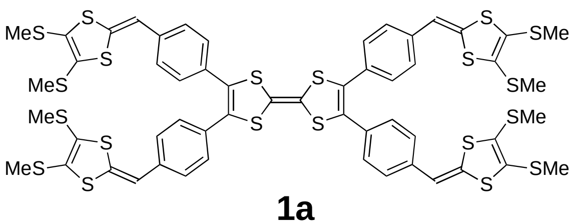

1a

^{13}C NMR of **1a**

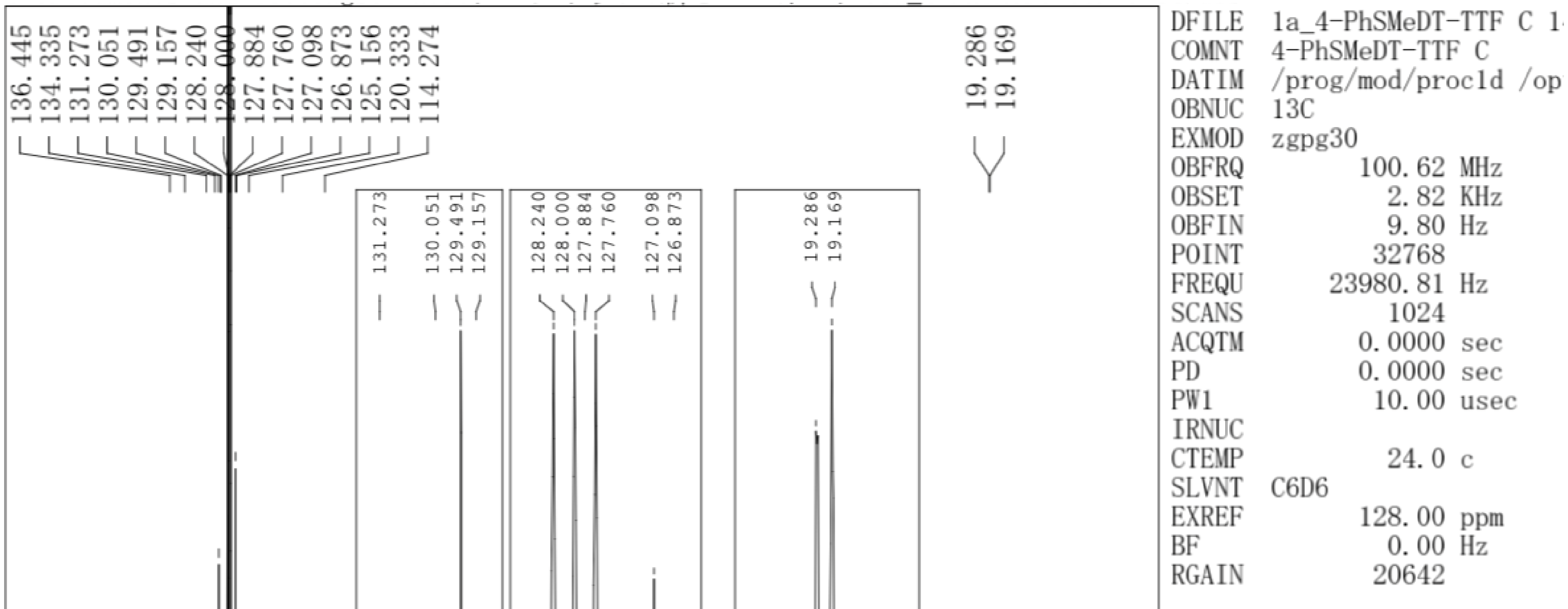

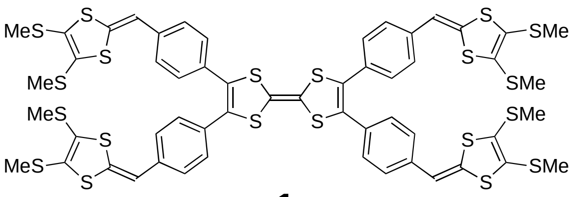

1a

^{1}H NMR of **1b**

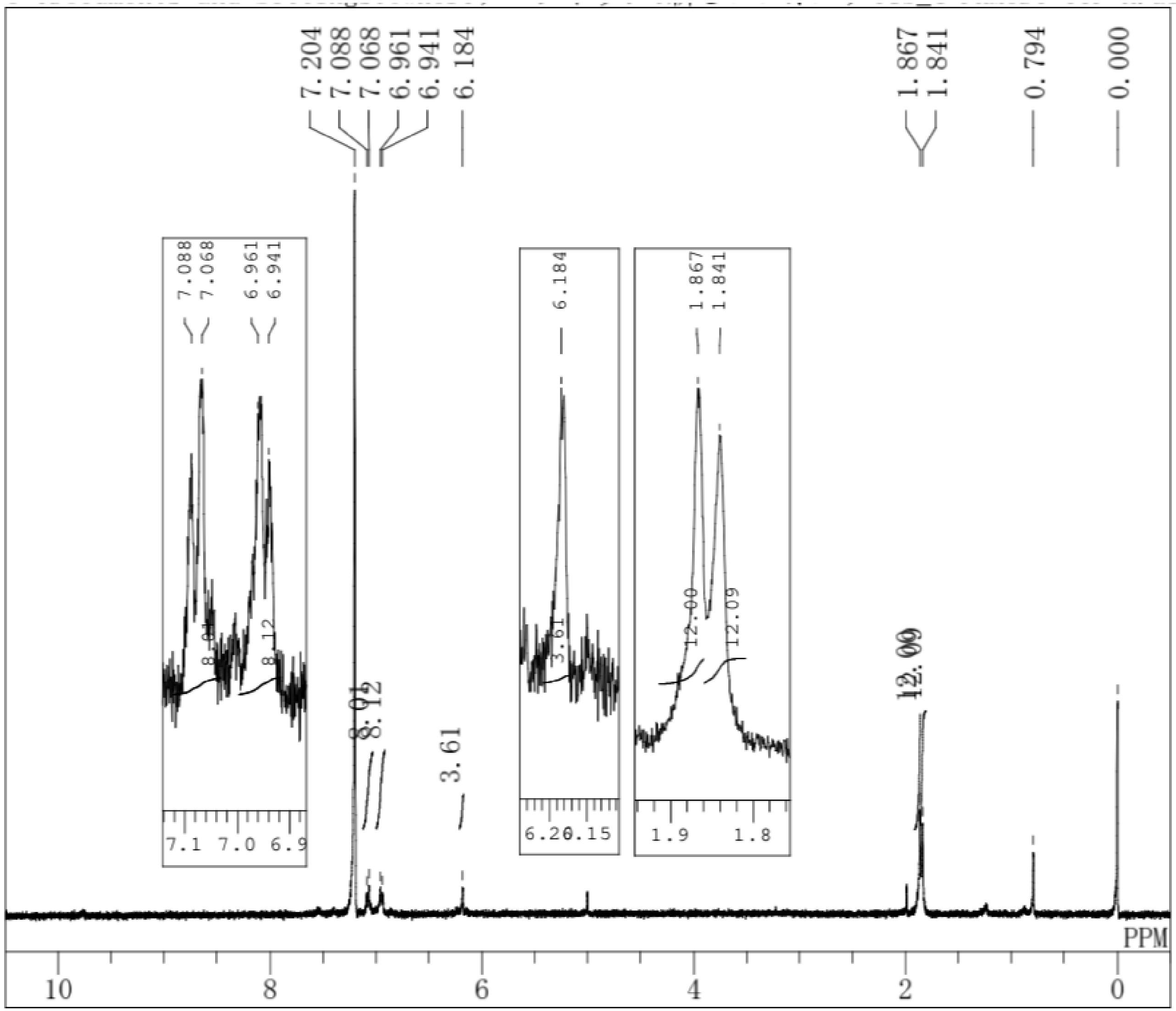

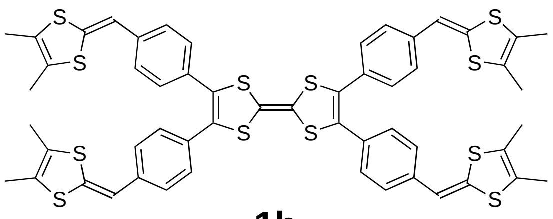

1b

^{13}C NMR of **1b**

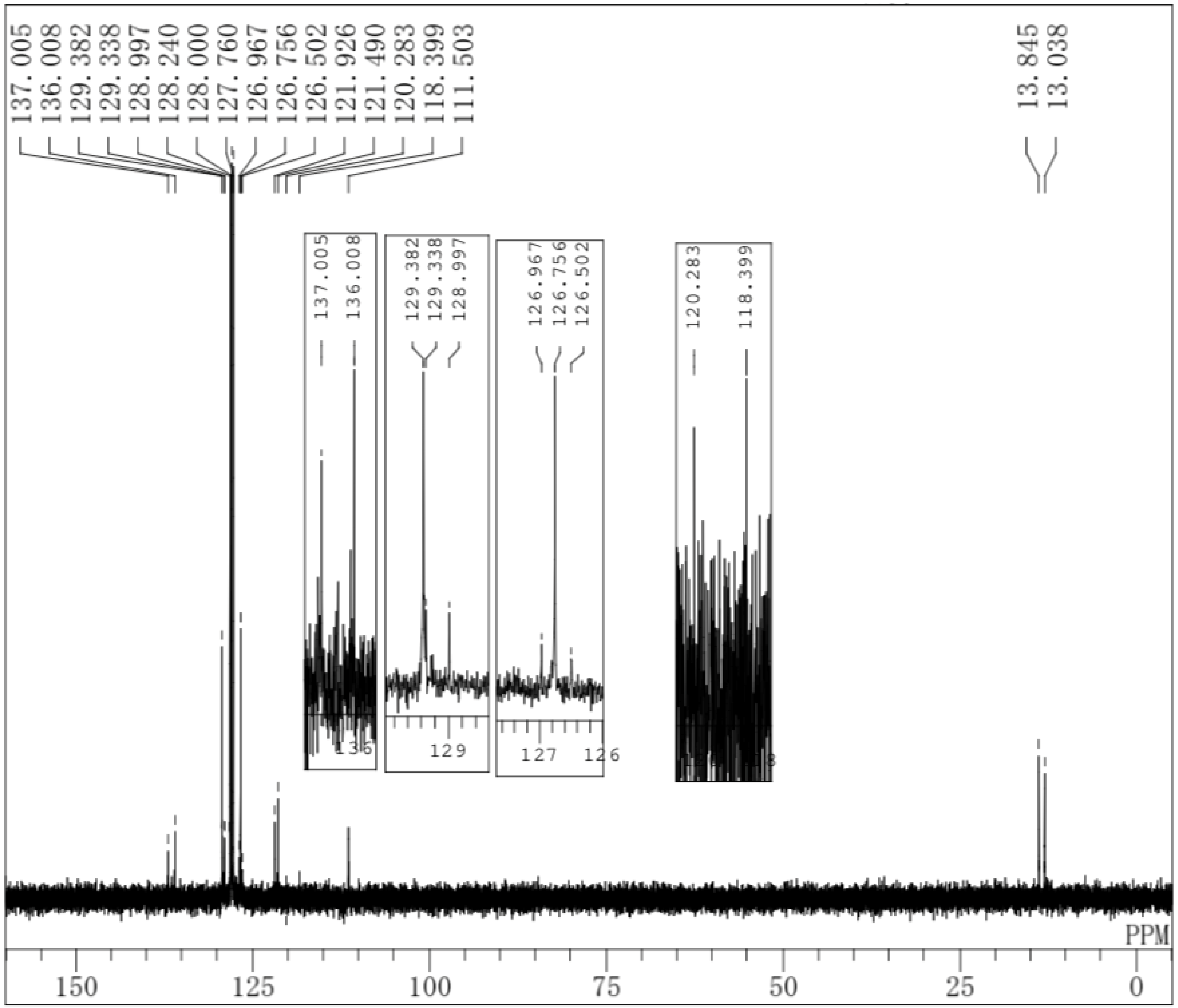

DFILE fid
COMNT 4-PhMeDT-TTF C
DATIM /prog/mod/proc1d /op
OBNUC 13C
EXMOD zgpg30
OBFRQ 100.62 MHz
OBSET 2.82 KHz
OBFIN 9.80 Hz
POINT 32768
FREQU 23980.81 Hz
SCANS 1024
ACQTM 0.0000 sec
PD 0.0000 sec
PW1 10.00 usec
IRNUC
CTEMP 23.9 c
SLVNT C6D6
EXREF 128.00 ppm
BF 0.00 Hz
RGAIN 20642

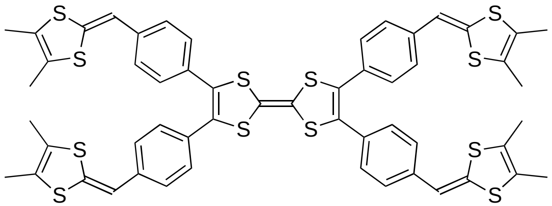

1b

^{1}H NMR of **2a**

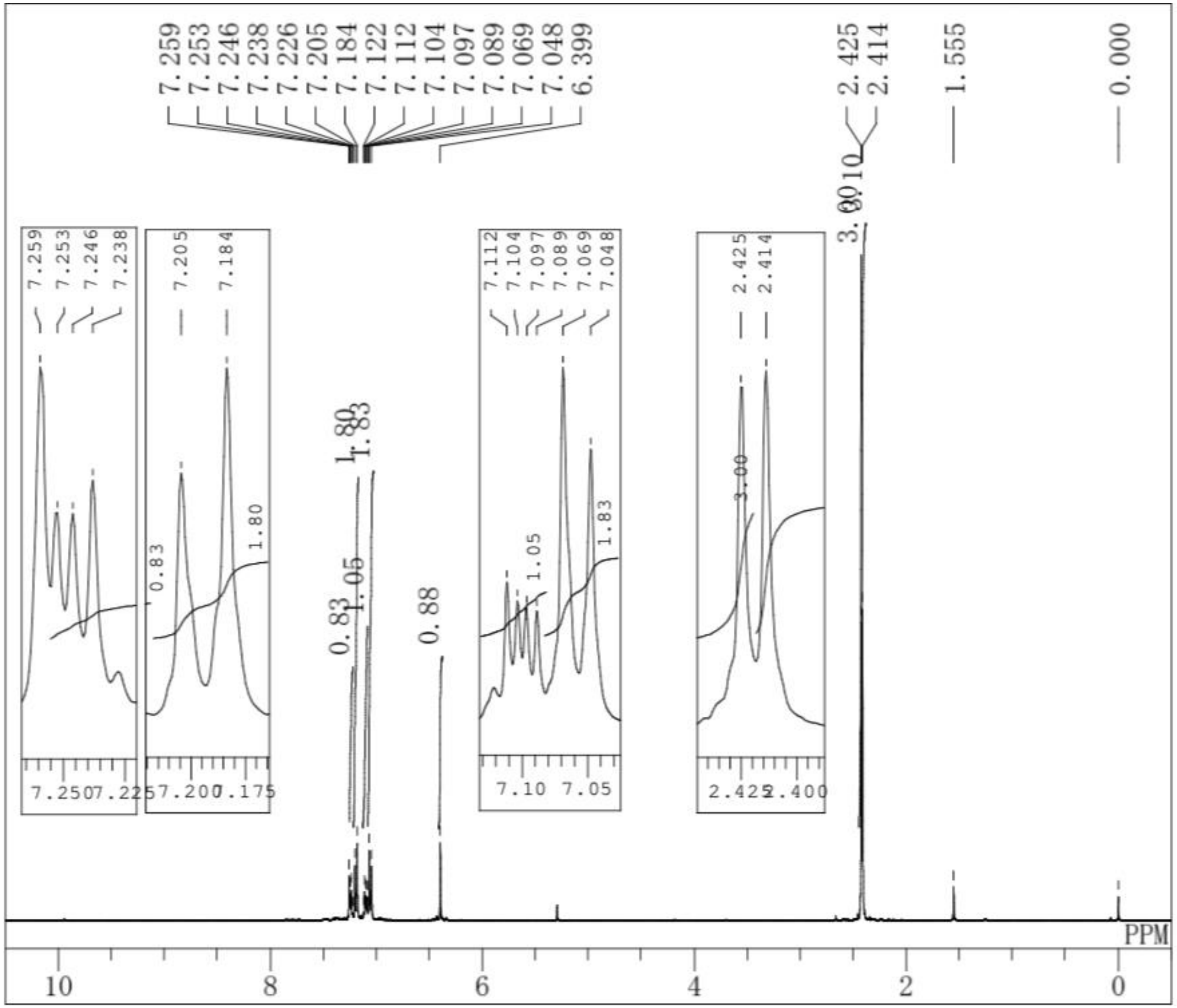

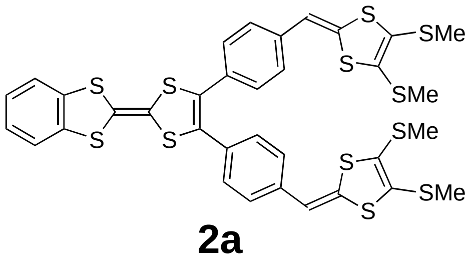

2a

^{13}C NMR of **2a**

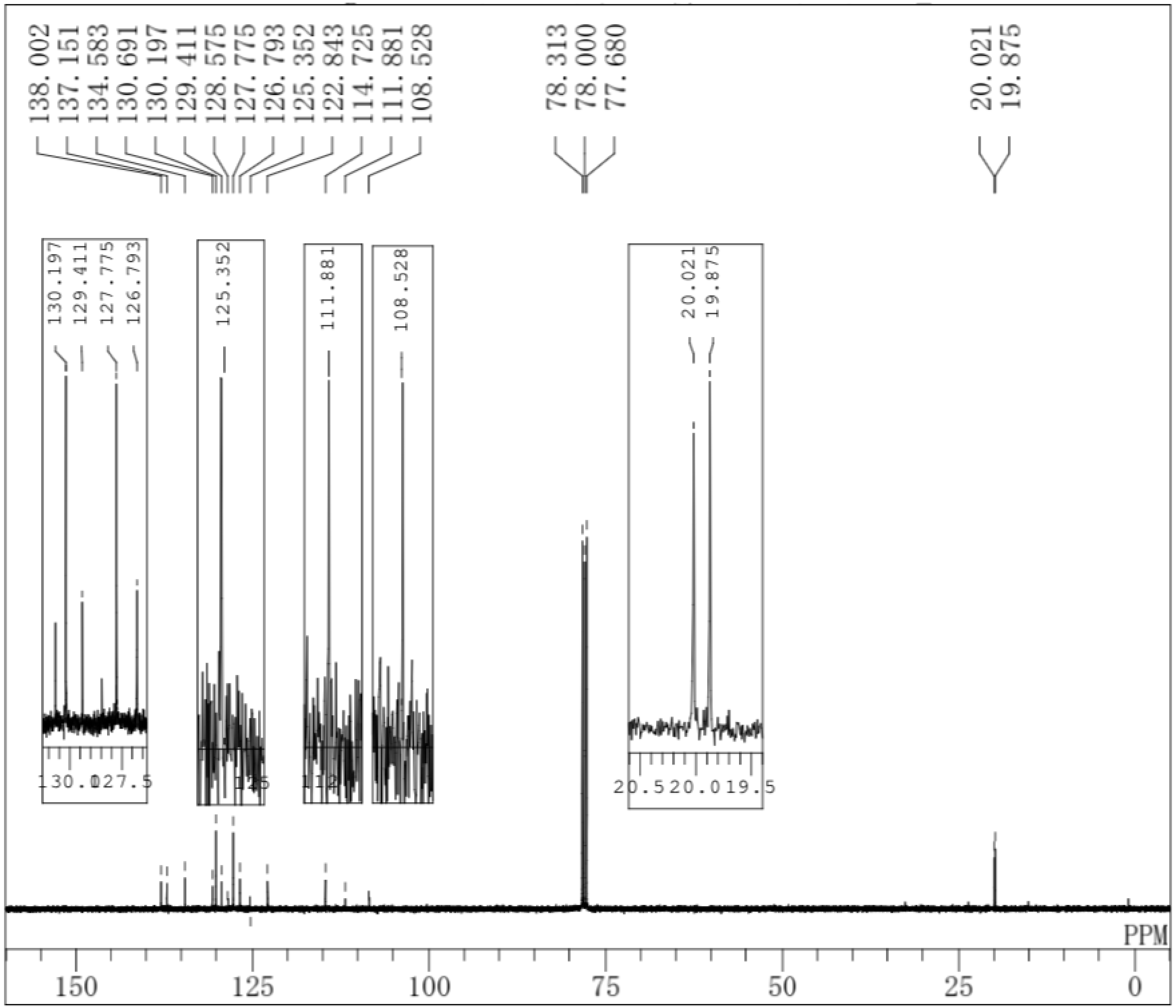

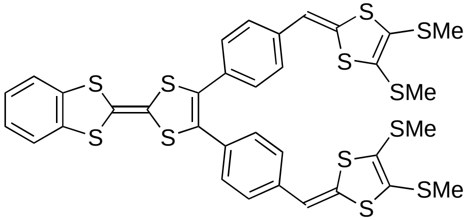

2a

^{1}H NMR of **2b**

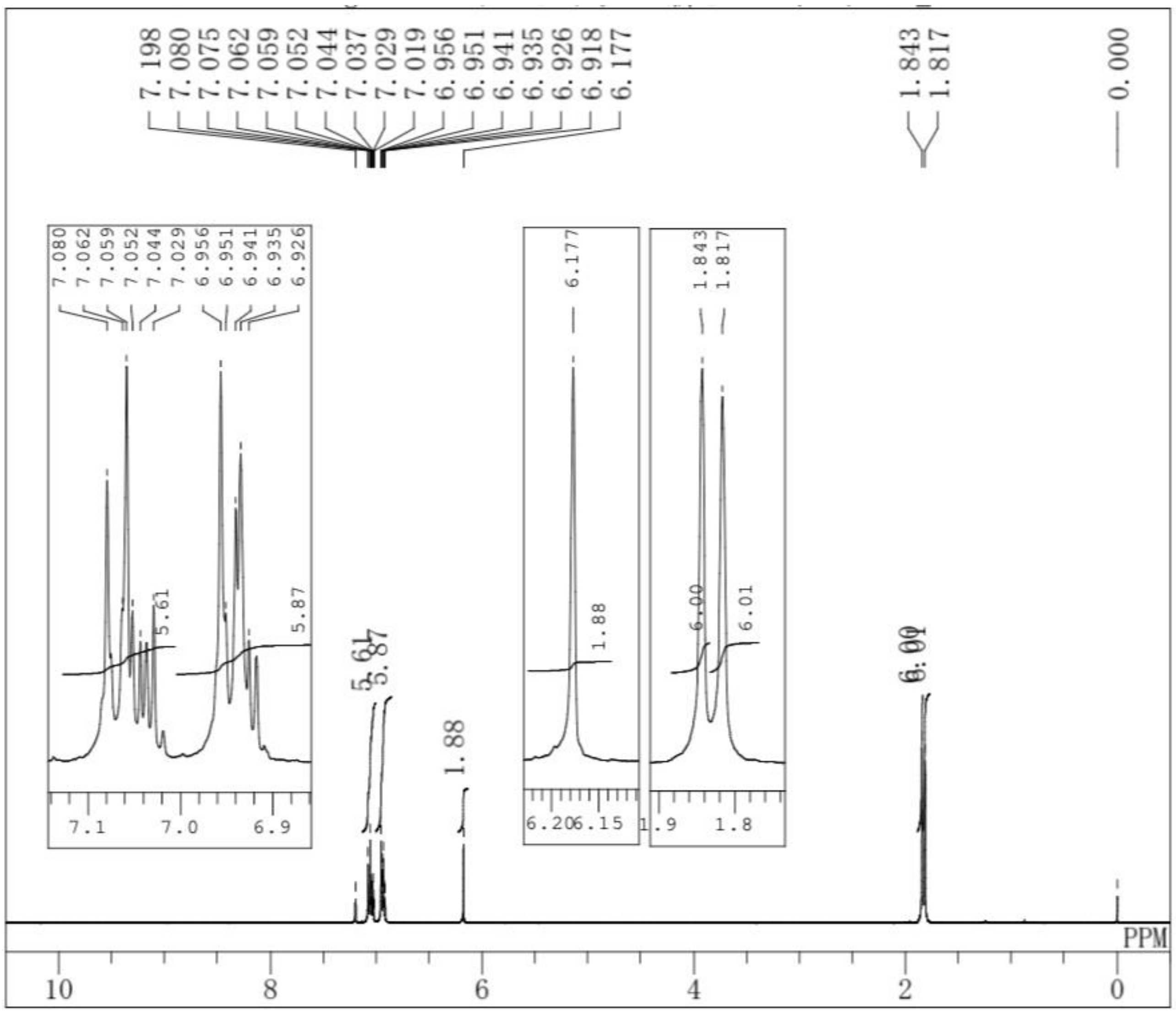

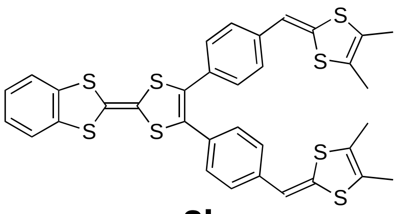

2b

^{13}C NMR of **2b**

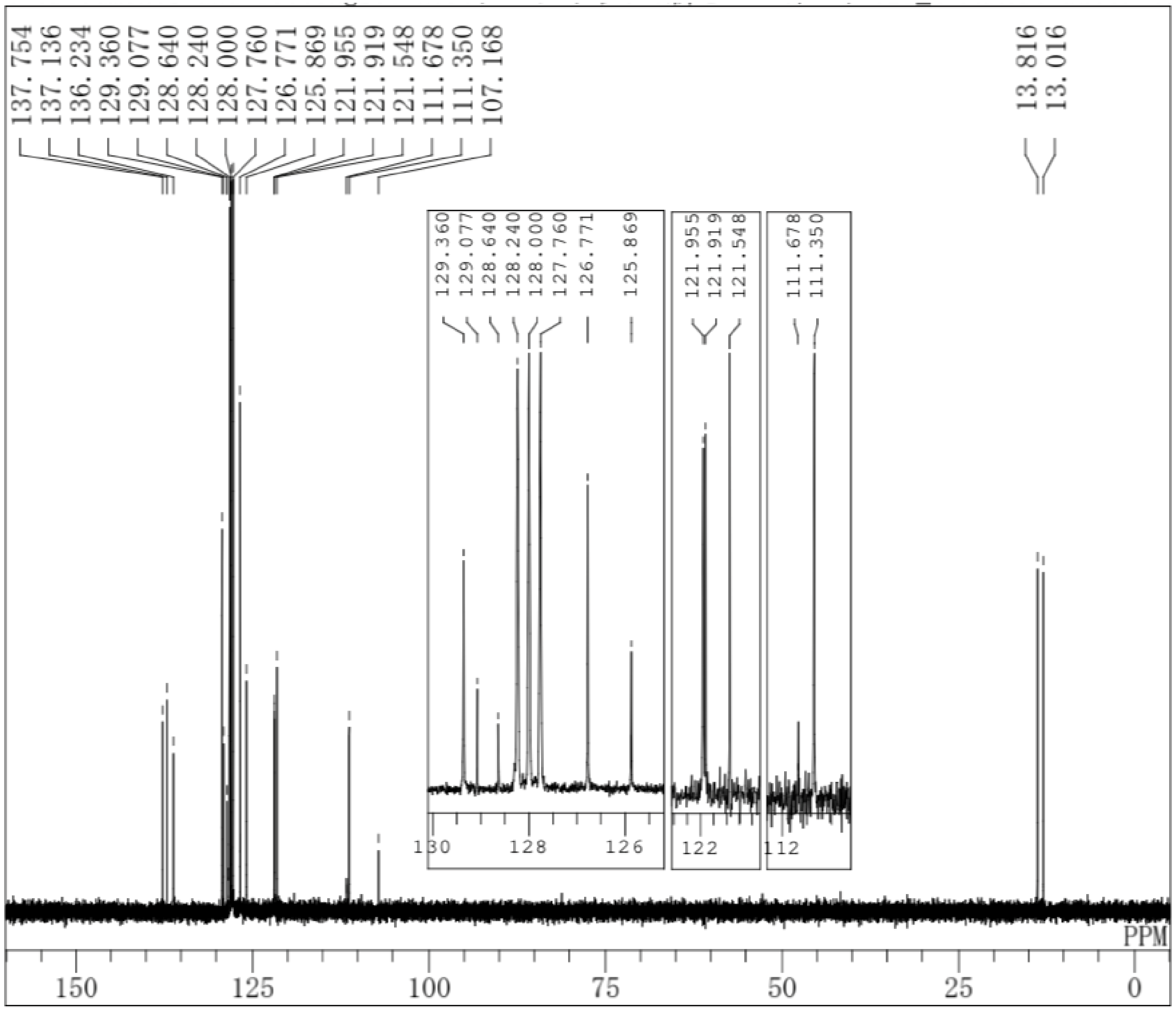

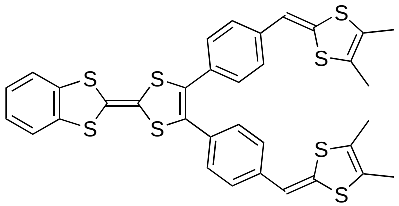

2b

^{1}H NMR of **3a**

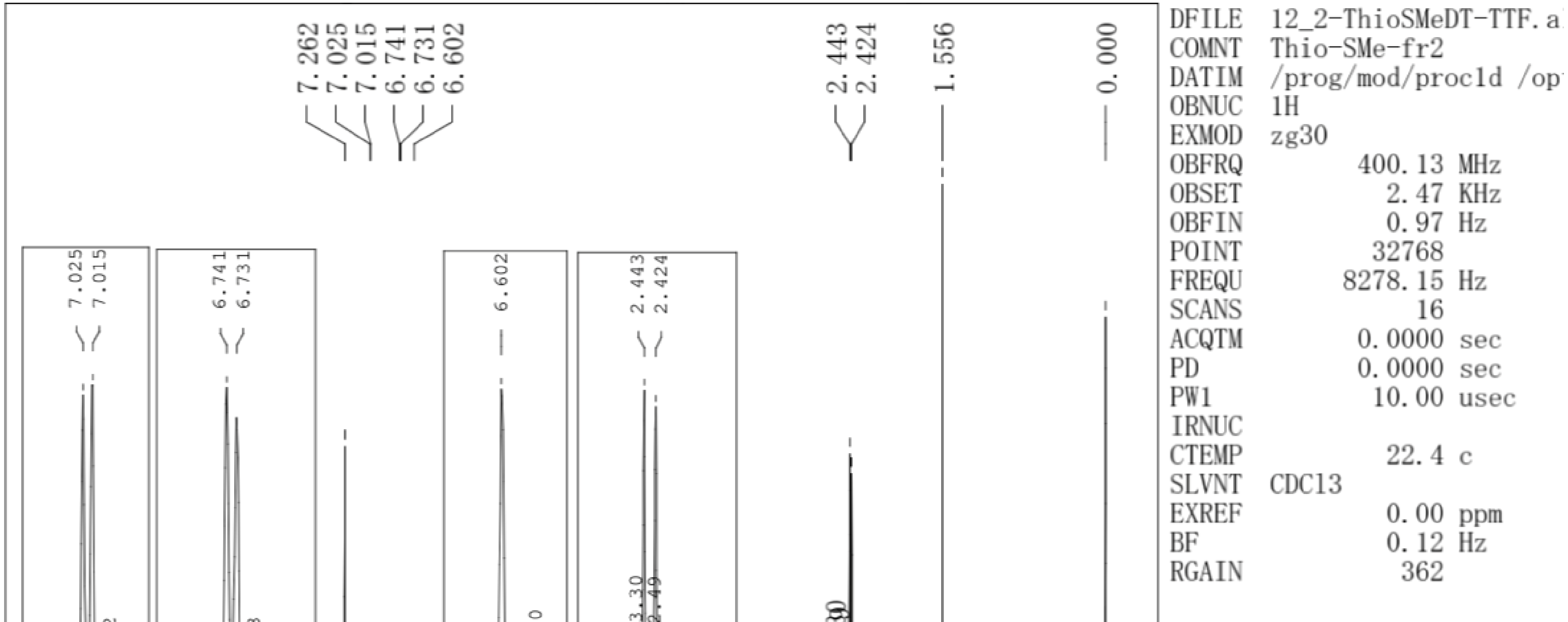

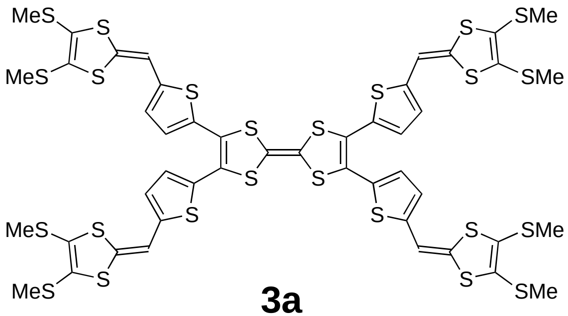

3a

^{1}H NMR of **4**

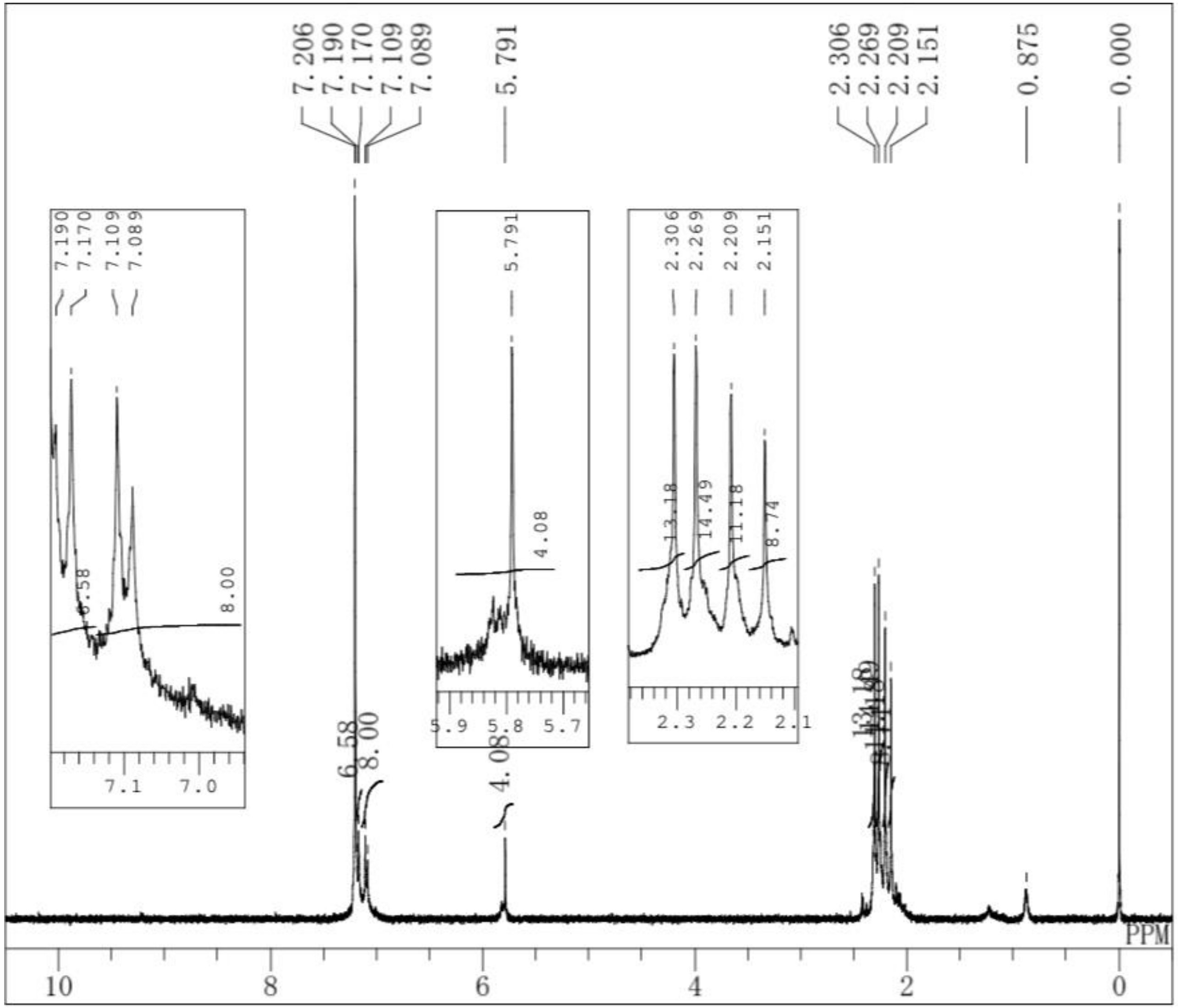
7.206
7.190
7.170
7.109
7.089
5.791
2.306
2.269
2.209
2.151
0.875
0.000
6.58
8.00
4.08
13.18
14.49
11.18
8.74
PPM
10
8
6
4
2
0
DFILE 4_4-PhSMeEBDT-TTF ar
COMNT 4-PhSMeEBDT-TTF aryl
DATIM /prog/mod/proc1d /op
OBNUC 1H
EXMOD zg30
OBFRQ 400.13 MHz
OBSET 2.47 KHz
OBFIN 0.97 Hz
POINT 32768
FREQU 8278.15 Hz
SCANS 16
ACQTM 0.0000 sec
PD 0.0000 sec
PW1 10.00 usec
IRNUC
CTEMP 22.7 c
SLVNT C6D6
EXREF 0.00 ppm
BF 0.00 Hz
RGAIN 362

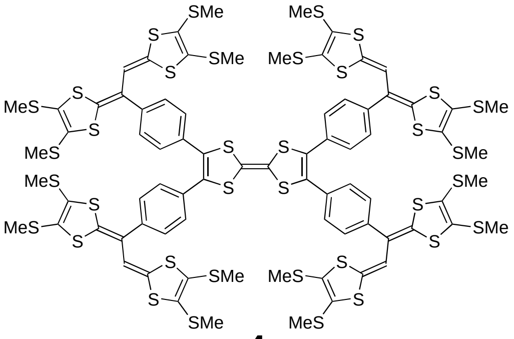
SMe
MeS
S
S

4

^{1}H NMR of **6a**

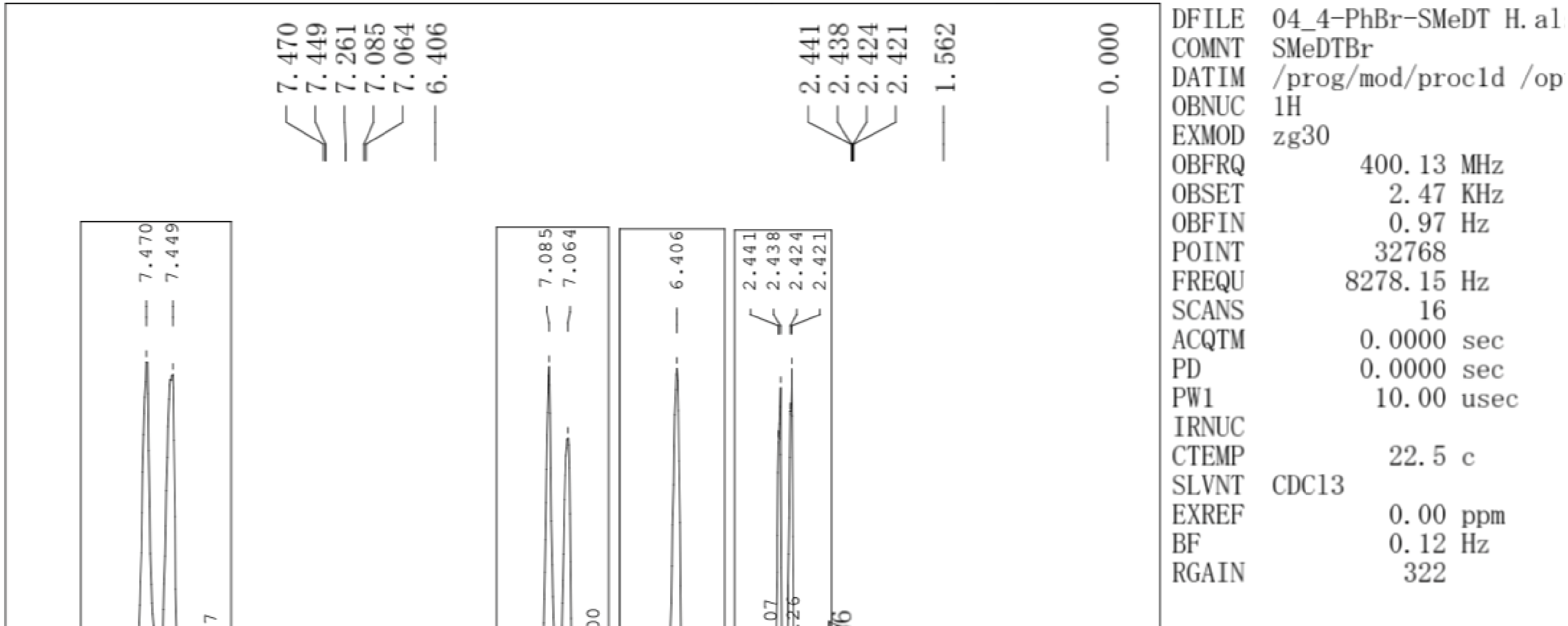

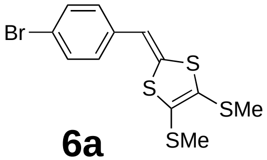

6a

^{13}C NMR of **6a**

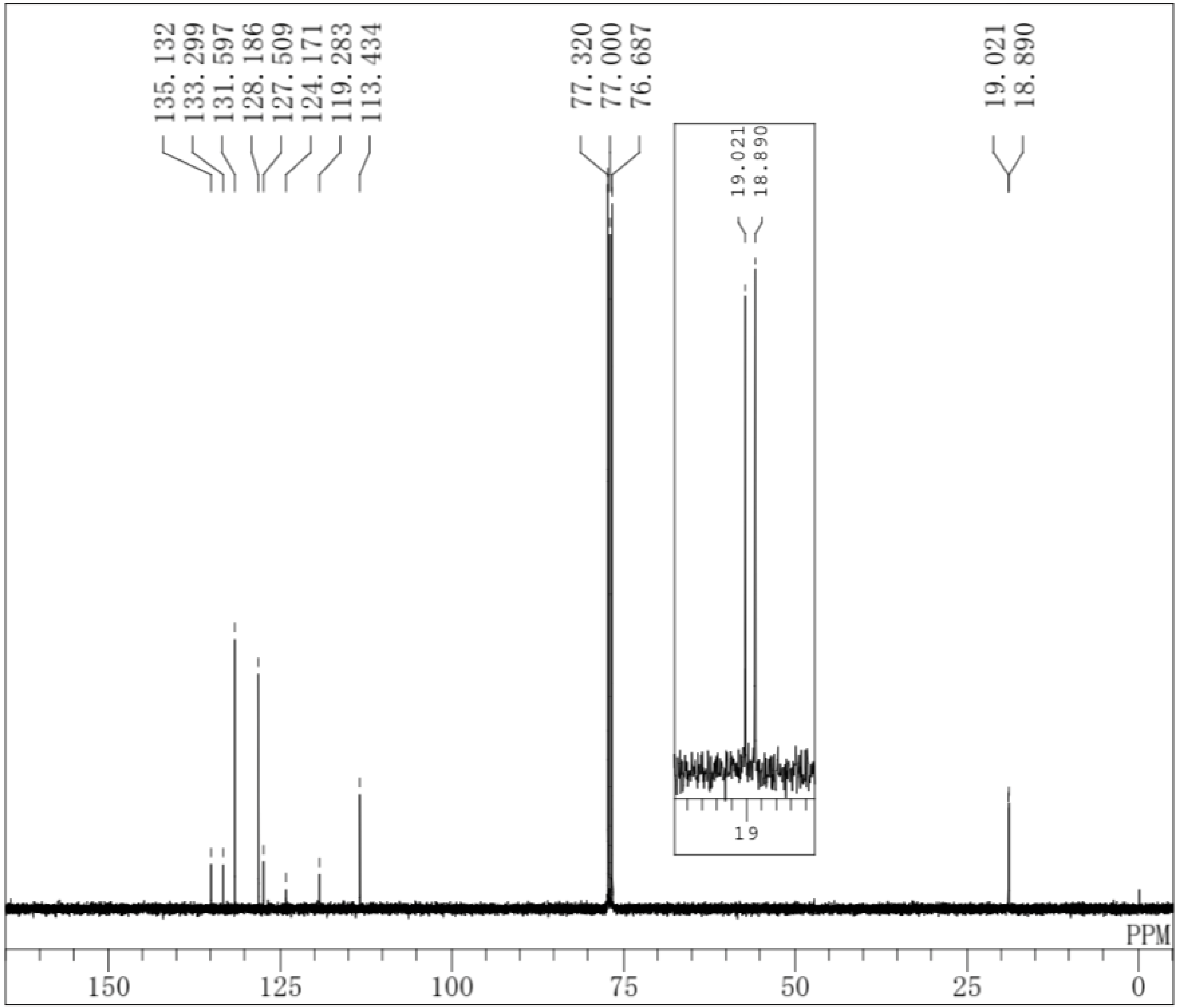

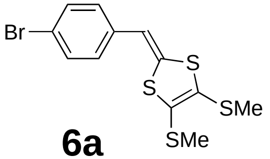

6a

^{1}H NMR of **6b**

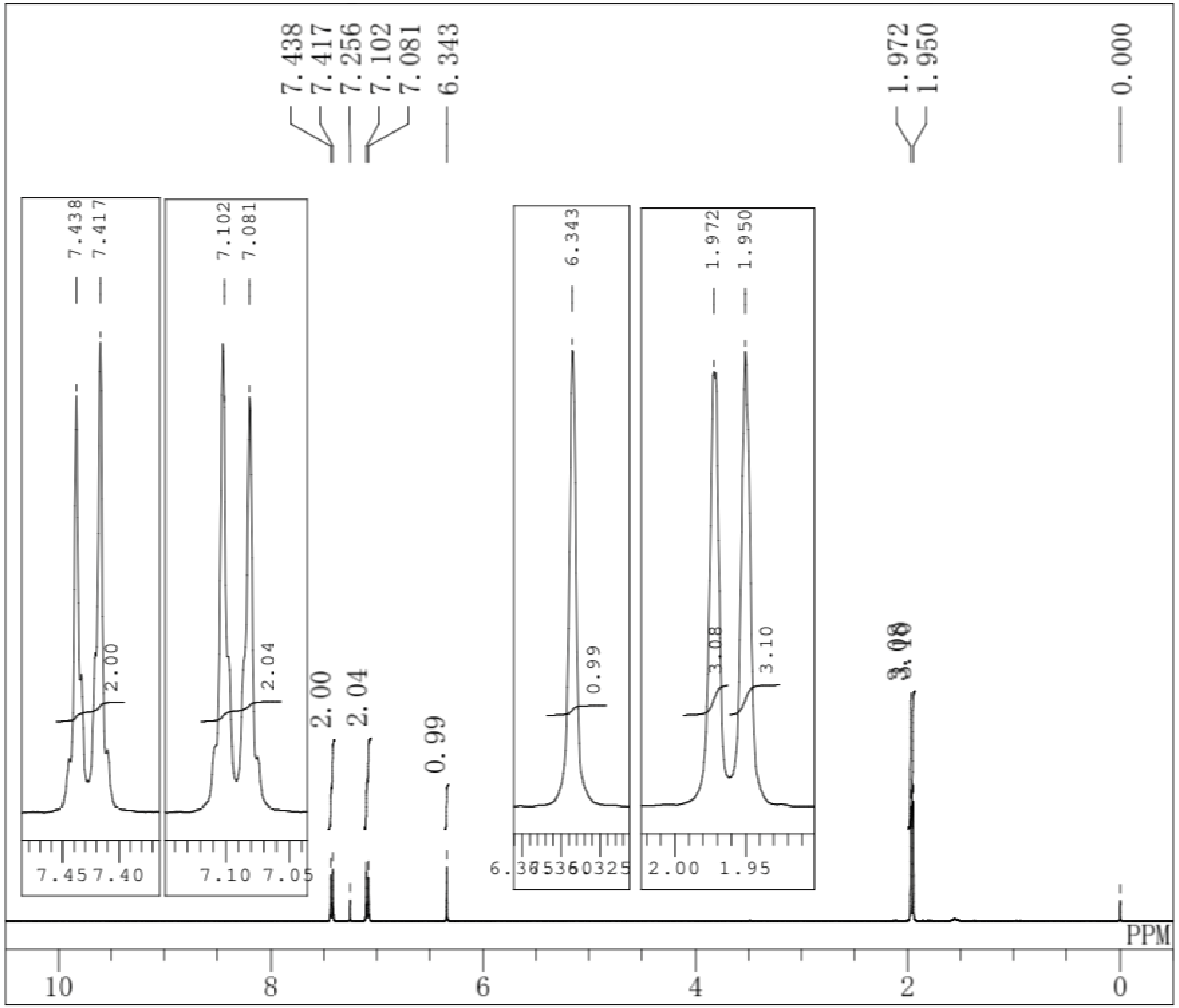

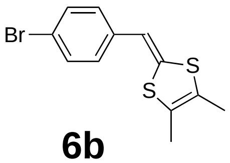

6b

^{13}C NMR of **6b**

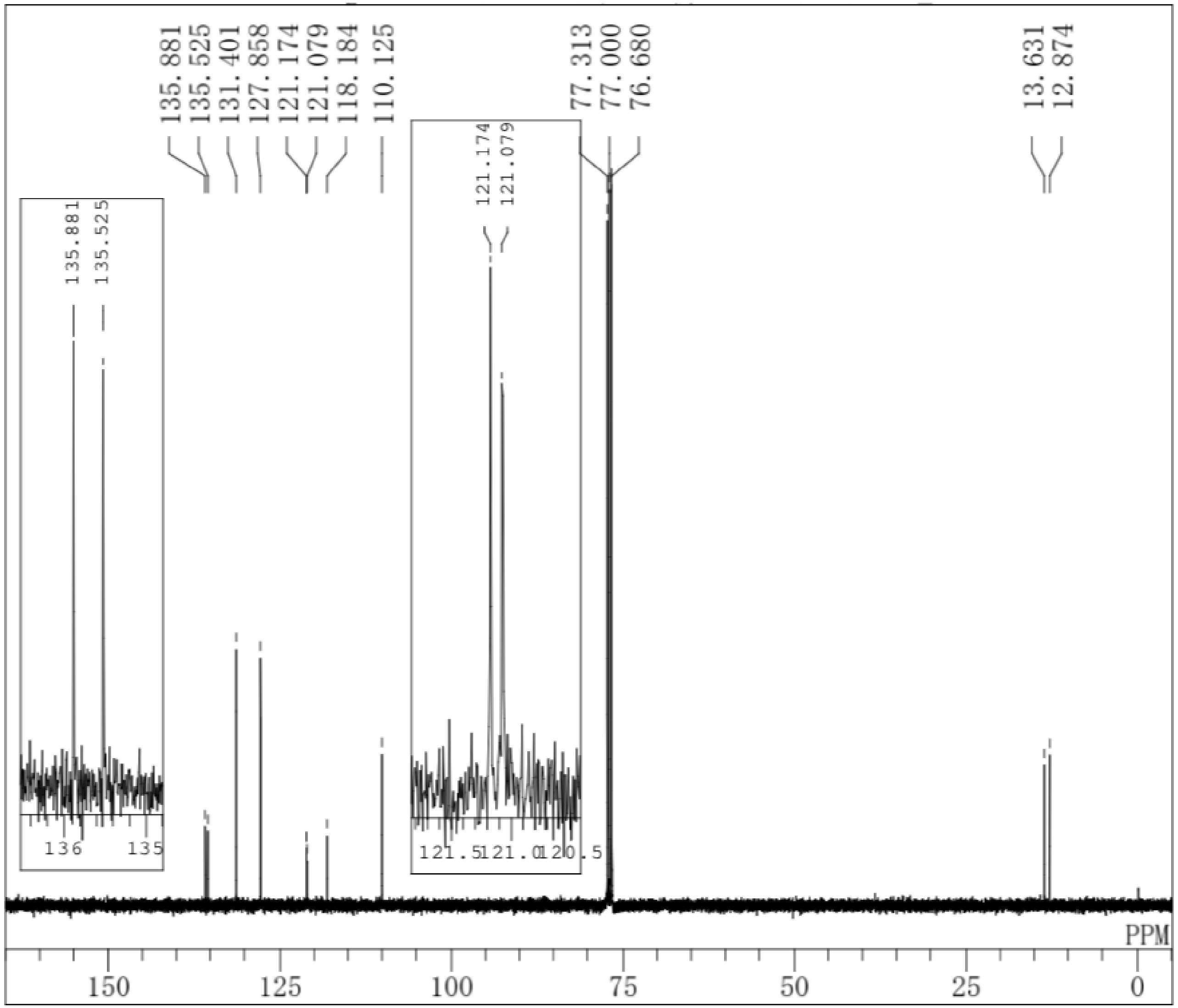

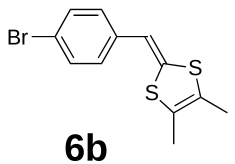

6b

^{1}H NMR of **6c**

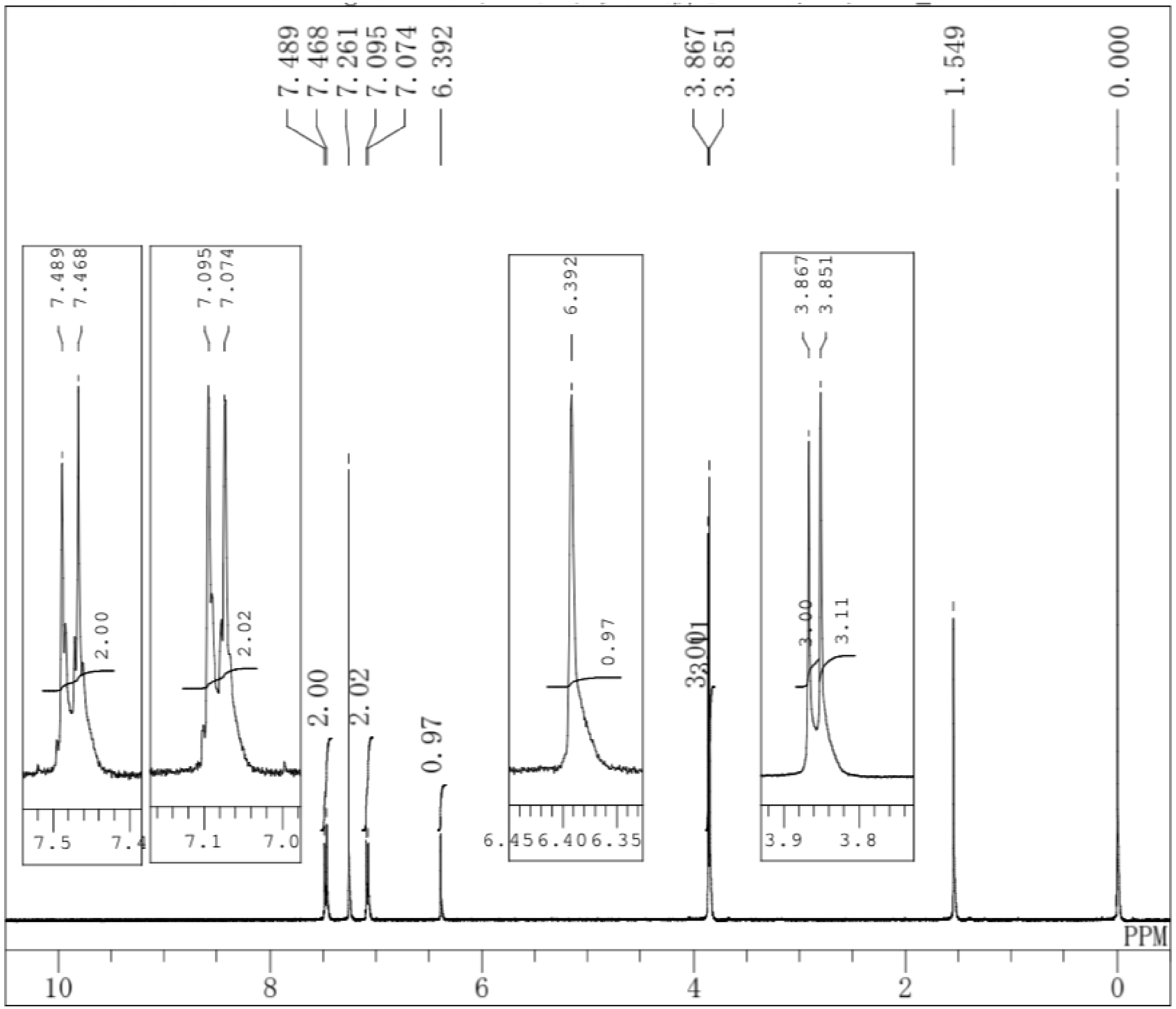

DFILE 6c_4-PhBr-CO2MeDT H.
COMNT BrBzDTCO2Me-1
DATIM /prog/mod/proc1d /op
OBNUC 1H
EXMOD zg30
OBFRQ 400.13 MHz
OBSET 2.47 KHz
OBFIN 0.97 Hz
POINT 32768
FREQU 8278.15 Hz
SCANS 16
ACQTM 0.0000 sec
PD 0.0000 sec
PW1 10.00 usec
IRNUC
CTEMP 23.3 c
SLVNT CDC13
EXREF 0.00 ppm
BF 0.00 Hz
RGAIN 456

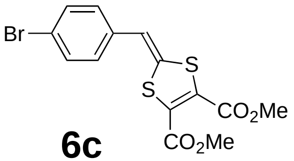

6c

^{13}C NMR of **6c**

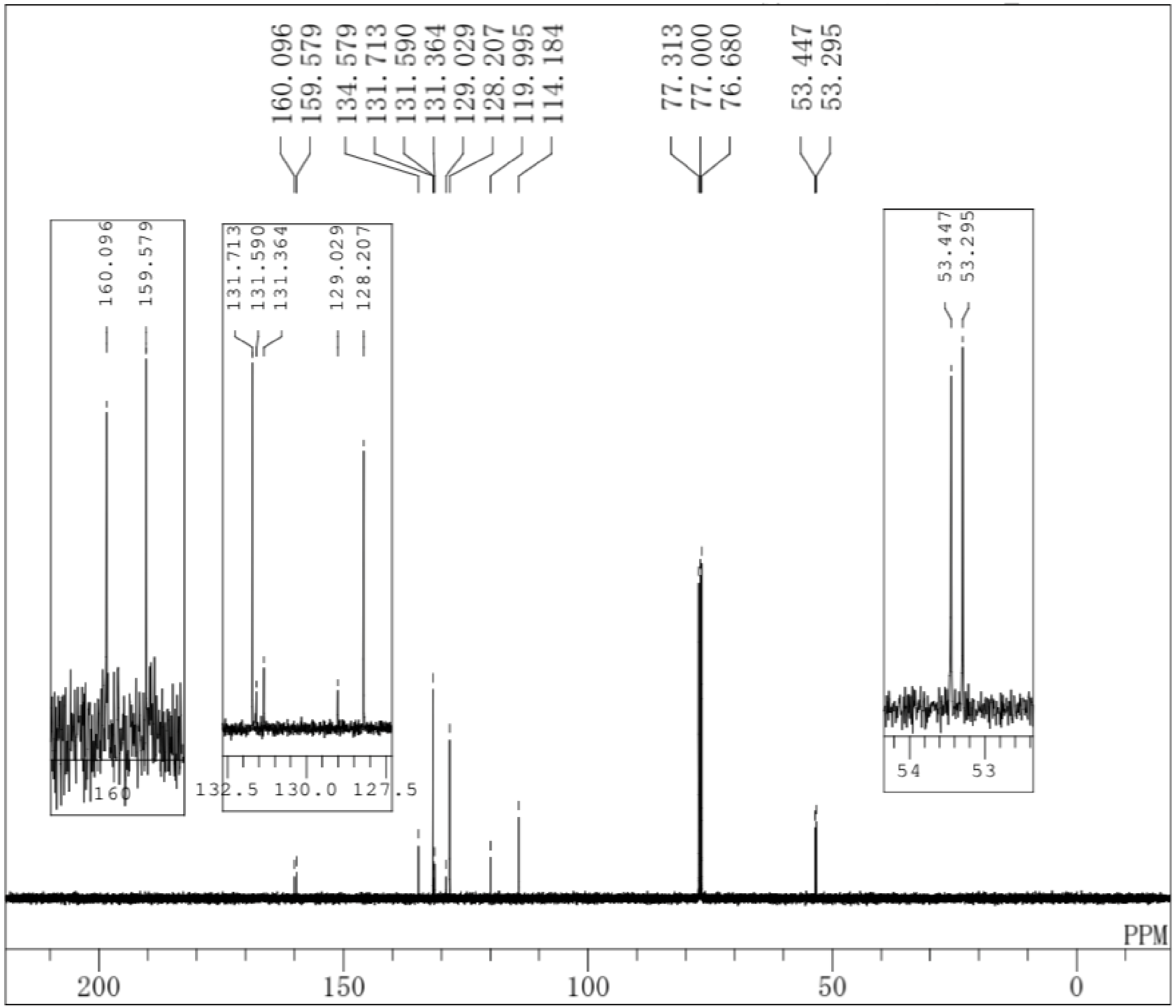

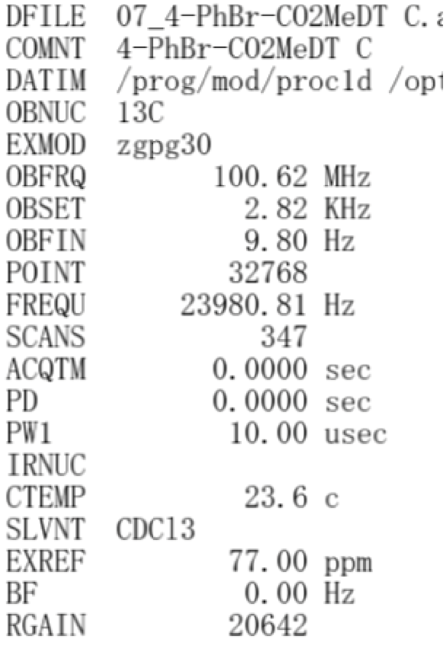

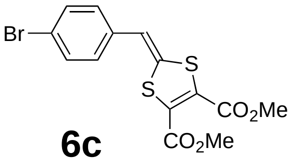

6c

^{1}H NMR of **6d**

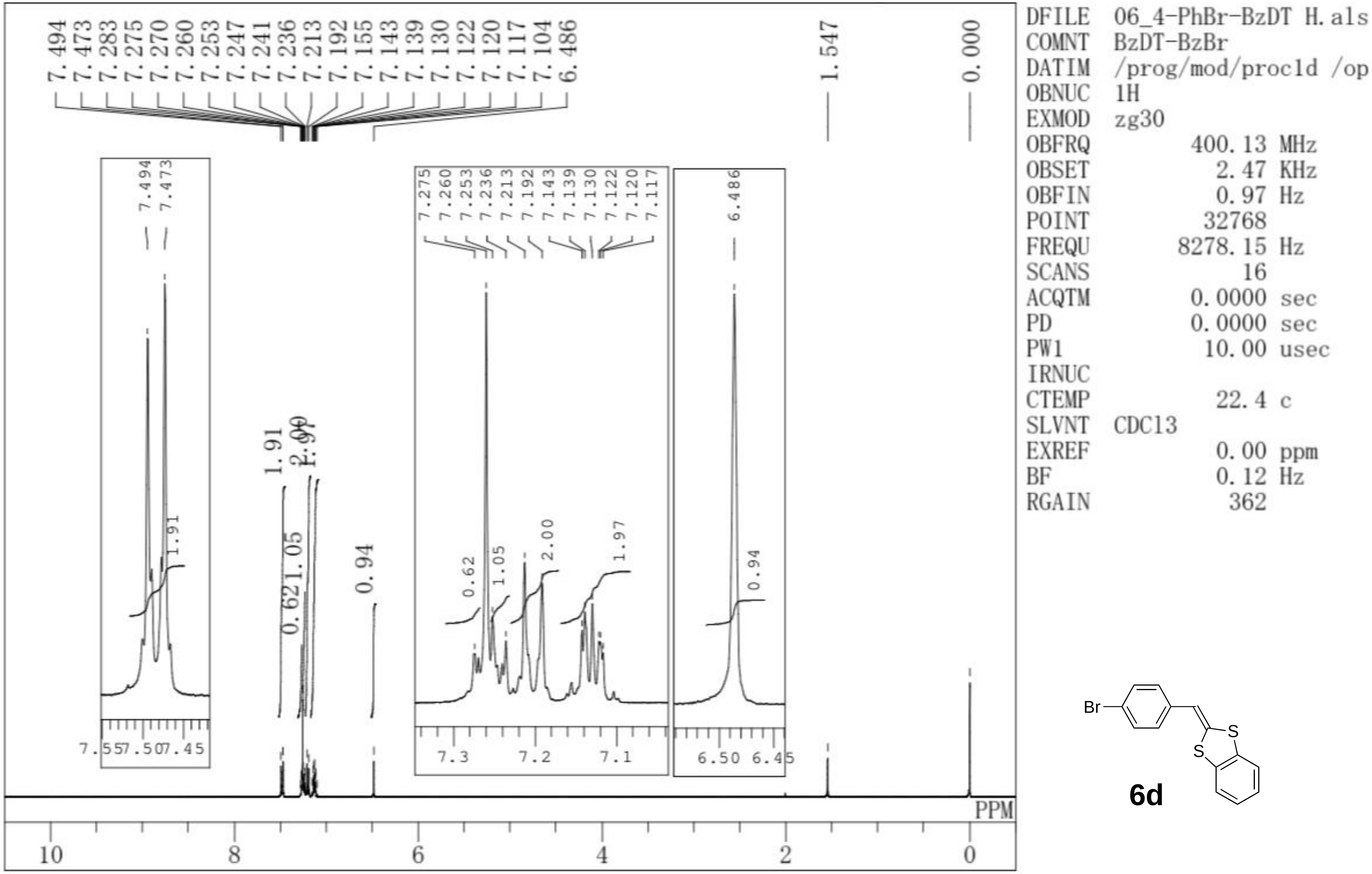

6d

^{13}C NMR of **6d**

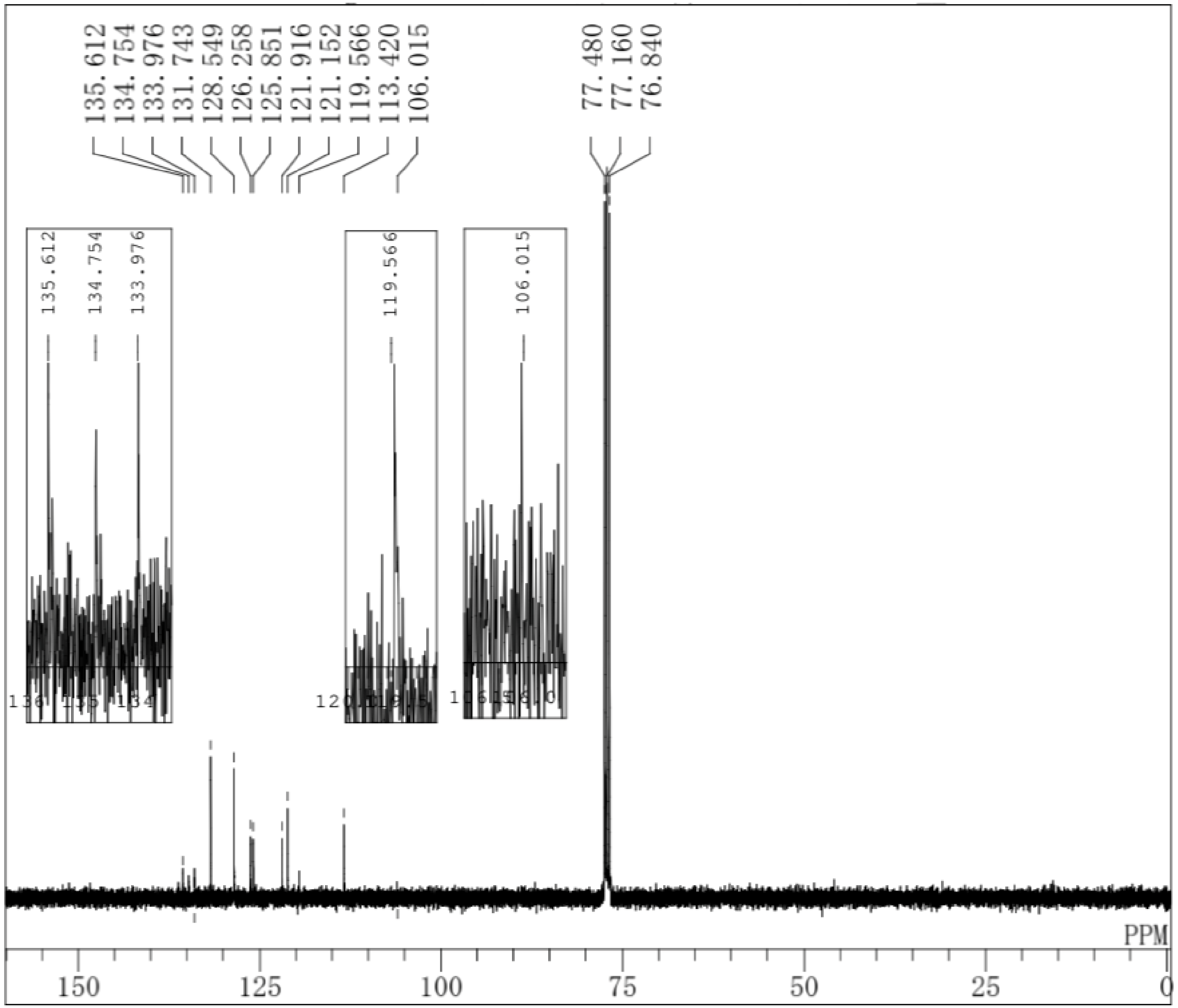

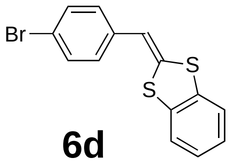

6d

^{1}H NMR of **7a**

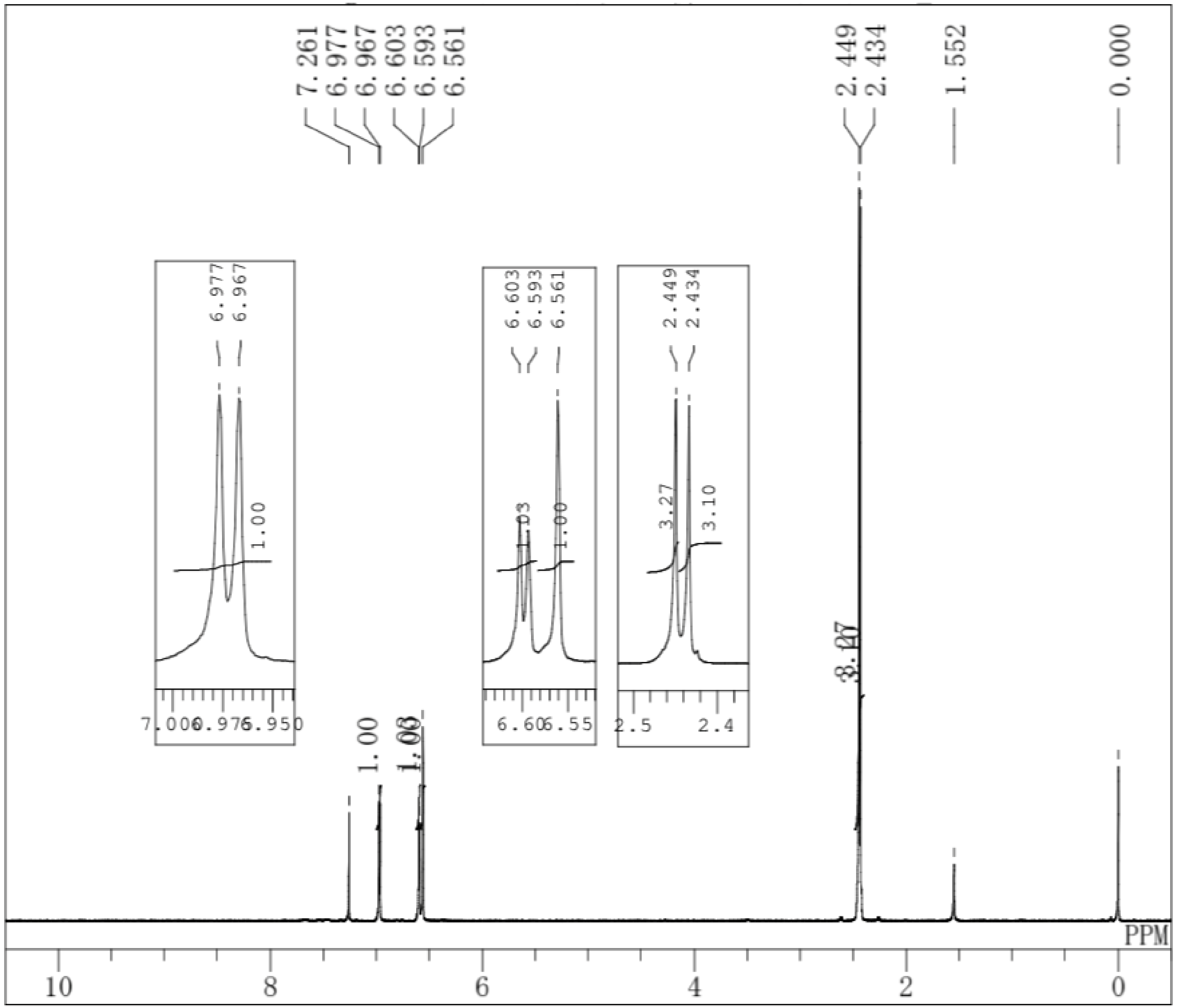

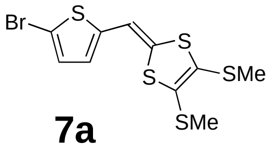

7a

^{13}C NMR of **7a**

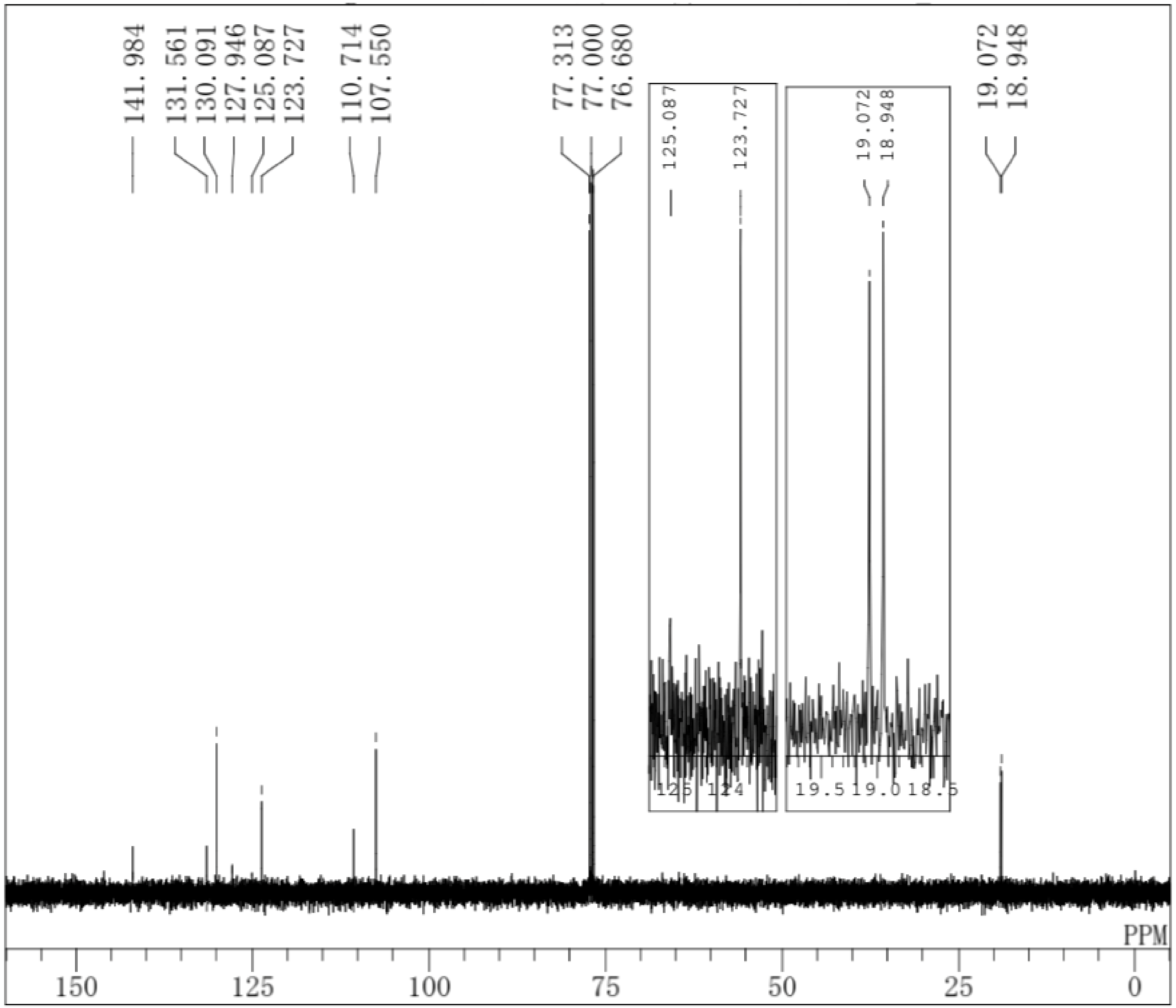

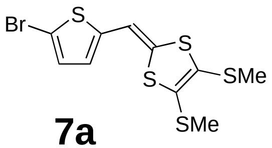

7a

^{1}H NMR of **7b**

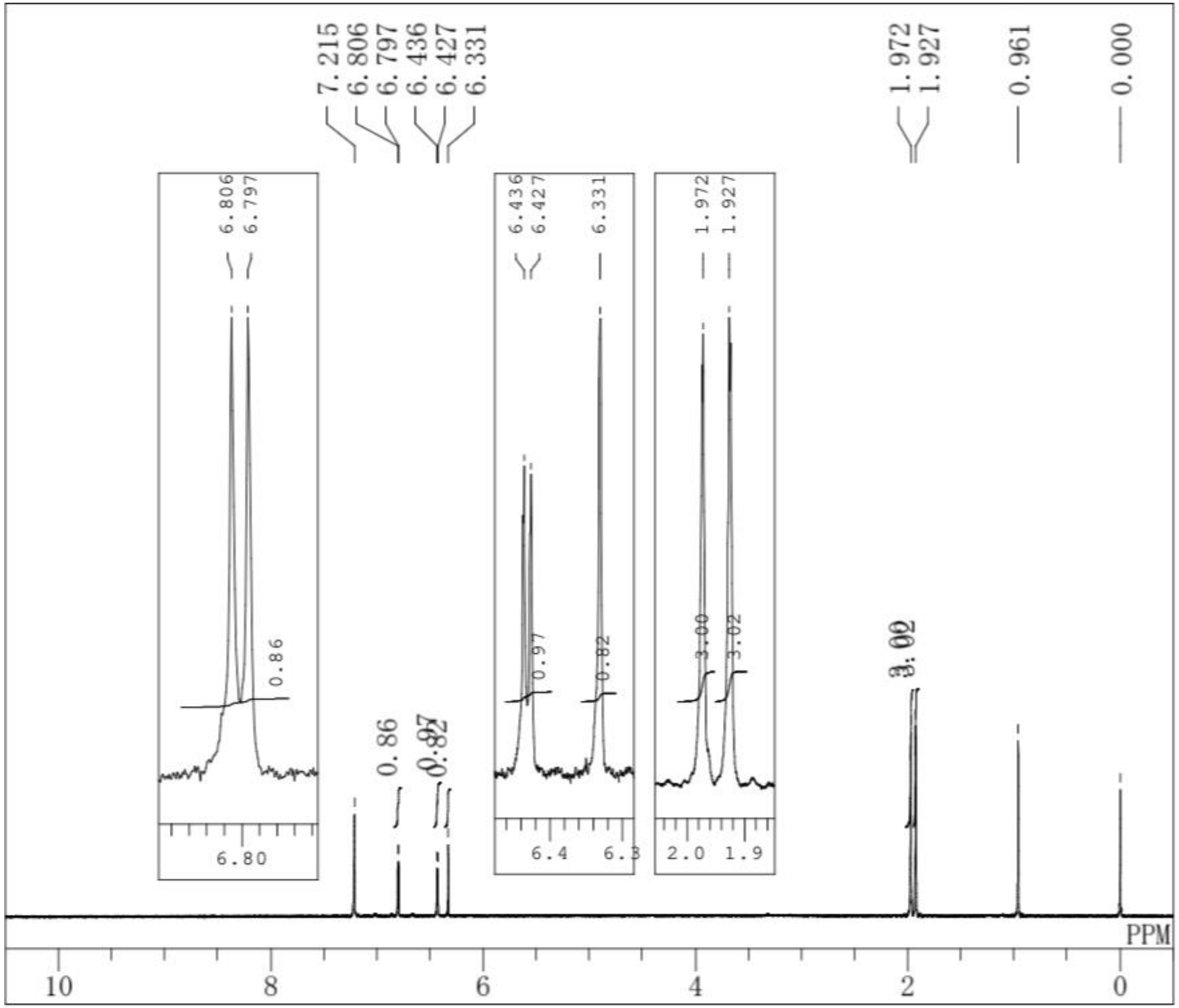

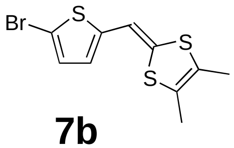

7b

^{13}C NMR of **7b**

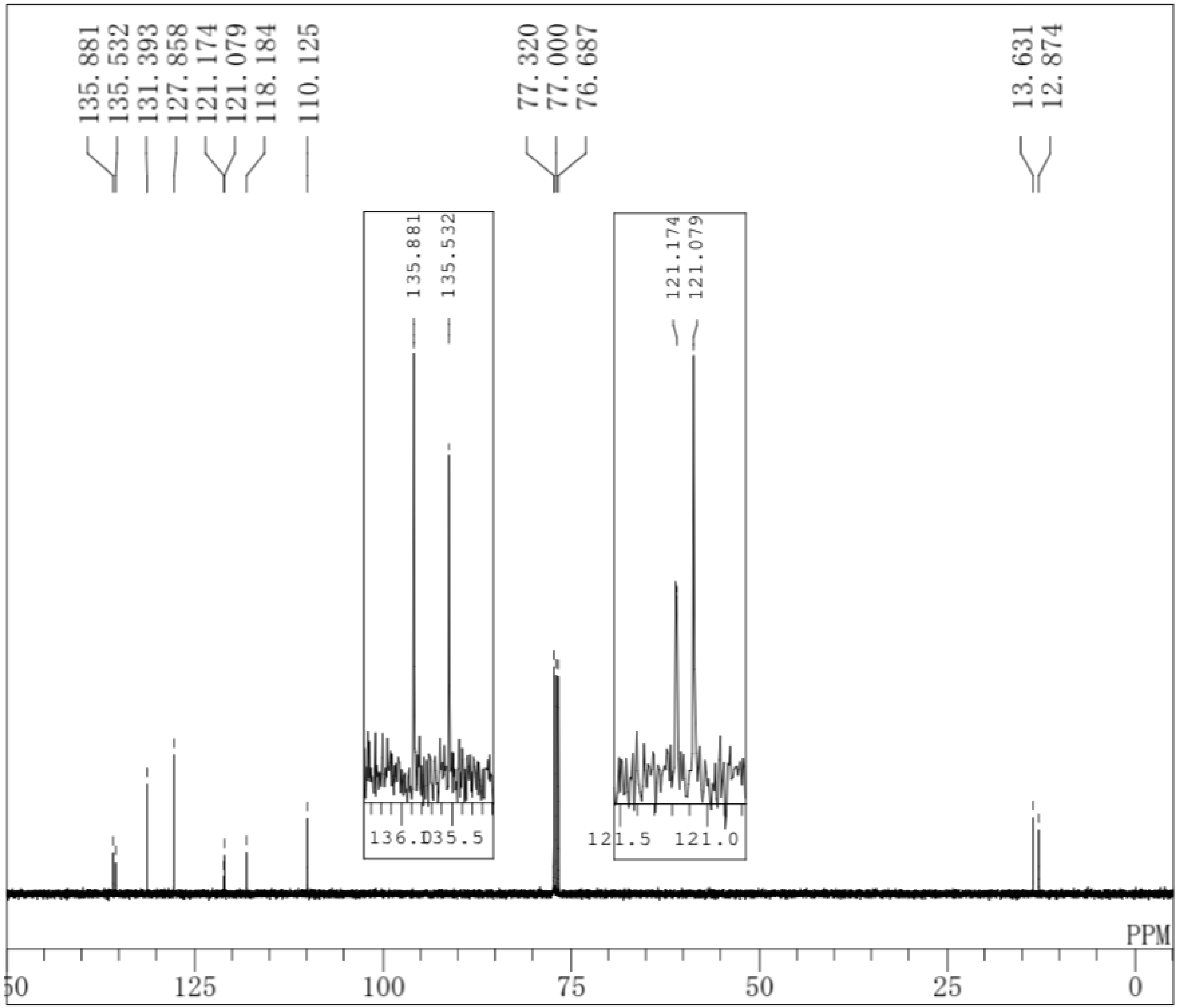

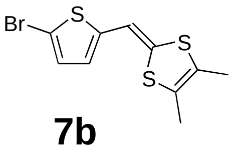

7b

^{1}H NMR of **7d**

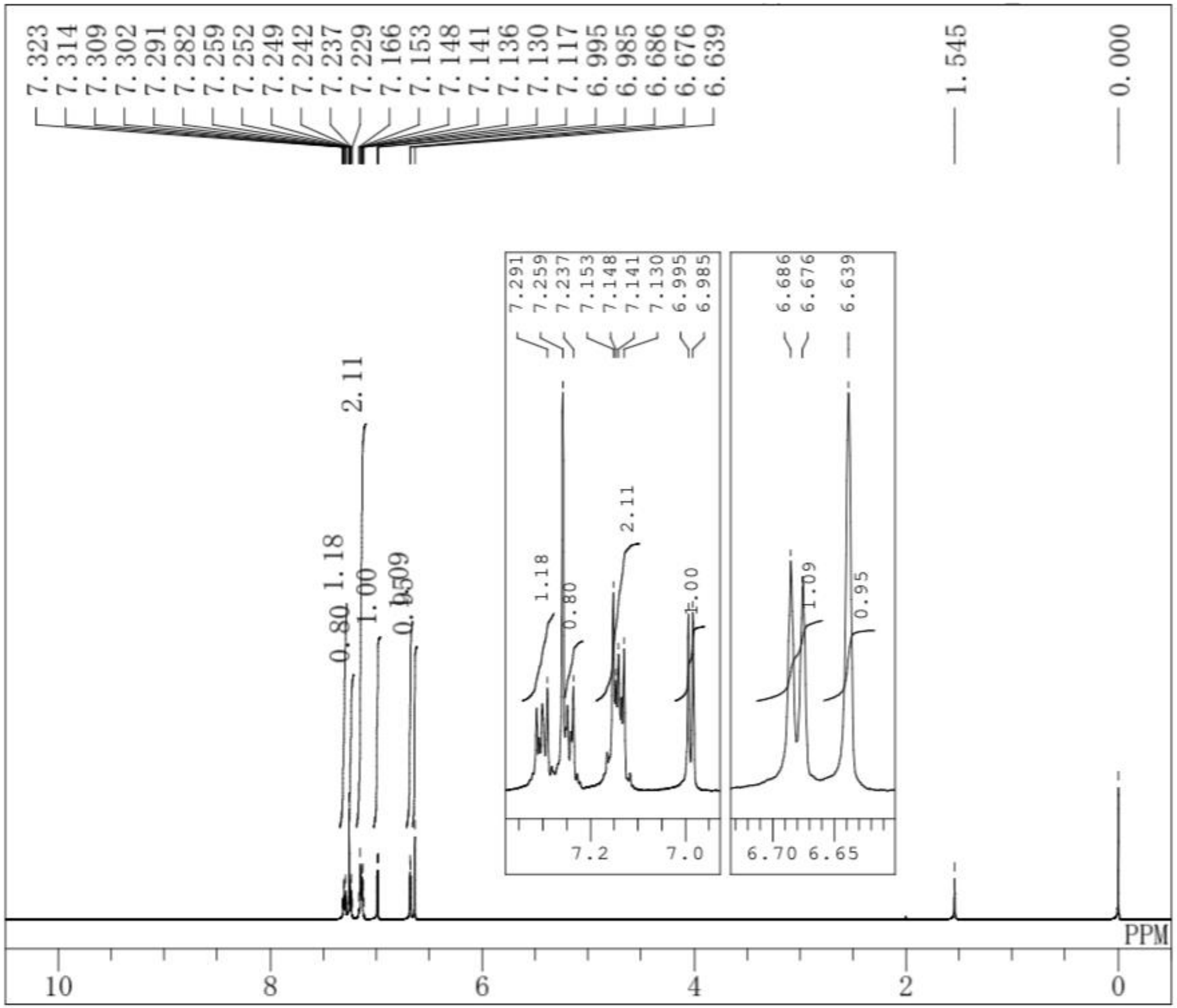

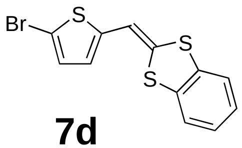

7d

^{13}C NMR of **7d**

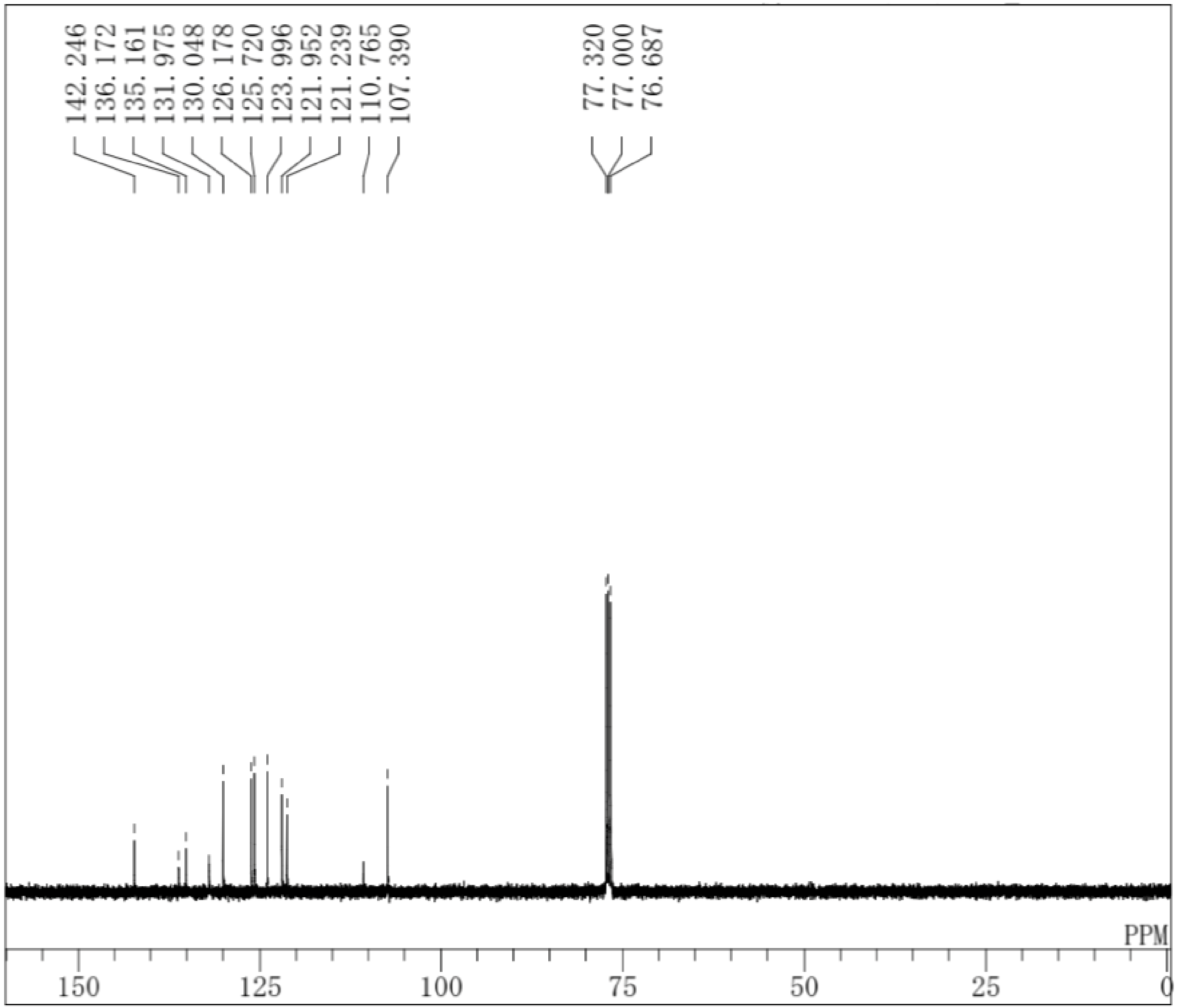

DFILE 03_2-ThioBr-BzDT C.a
COMNT 4-PhBr-BzDT
DATIM /prog/mod/proc1d /op
OBNUC 13C
EXMOD zgpg30
OBFRQ 100.62 MHz
OBSET 2.82 KHz
OBFIN 9.80 Hz
POINT 32768
FREQU 23980.81 Hz
SCANS 518
ACQTM 0.0000 sec
PD 0.0000 sec
PW1 10.00 usec
IRNUC
CTEMP 23.9 c
SLVNT CDC13
EXREF 77.00 ppm
BF 0.12 Hz
RGAIN 20642

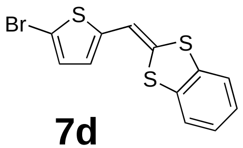

7d

^{1}H NMR of **9**

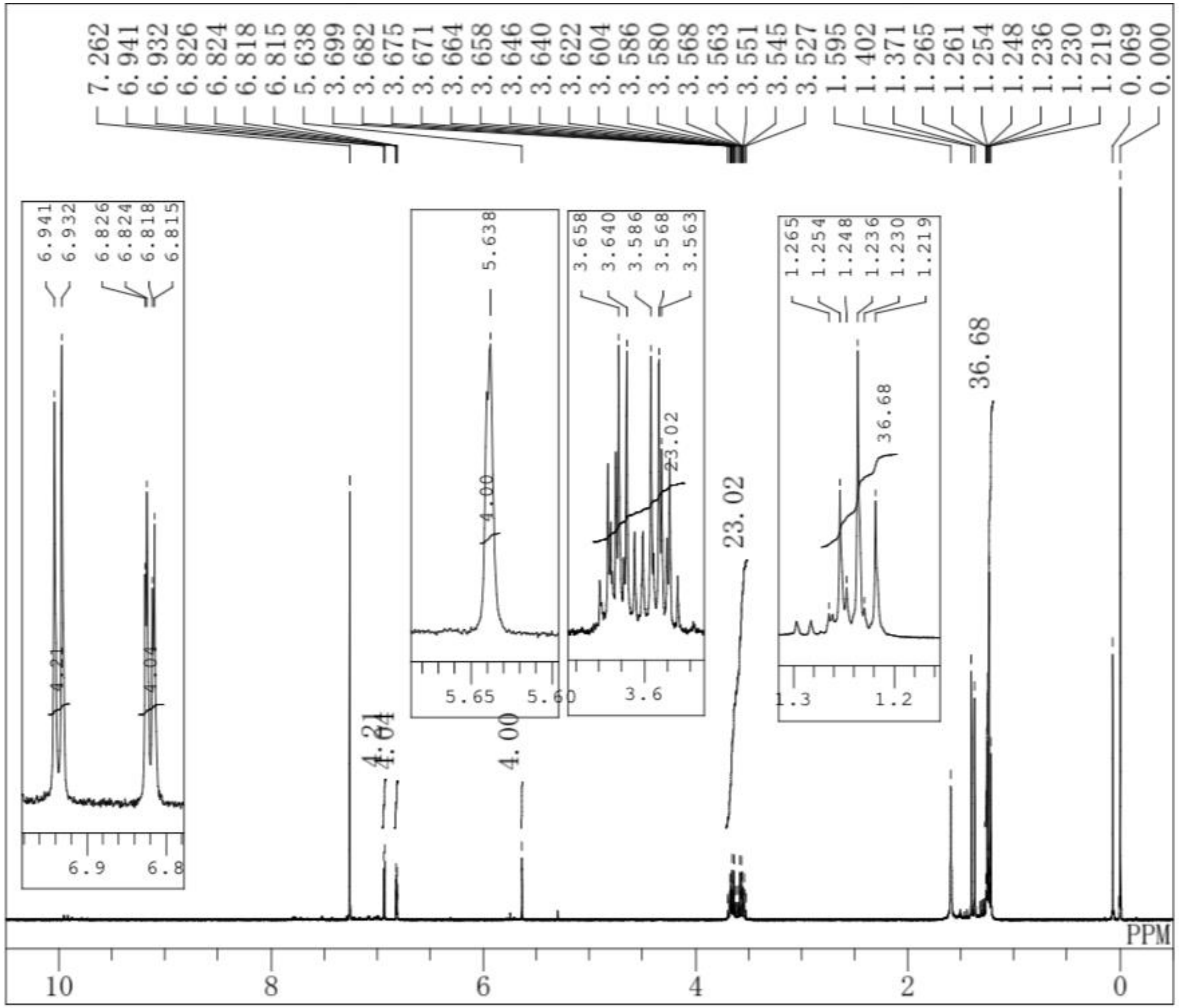

7.262
6.941
6.932
6.826
6.824
6.818
6.815
5.638
3.699
3.682
3.675
3.671
3.664
3.658
3.646
3.640
3.622
3.604
3.586
3.580
3.568
3.563
3.551
3.545
3.527
1.595
1.402
1.371
1.265
1.261
1.254
1.248
1.236
1.230
1.219
0.069
0.000
4.21
4.04
4.00
23.02
36.68
PPM
DFILE 9_2-ThioCHOEt2精製せ
COMNT TTF-4Ar-thio-CH(OEt)
DATIM /prog/mod/proc1d /op
OBNUC 1H
EXMOD zg30
OBFRQ 400.13 MHz
OBSET 2.47 KHz
OBFIN 0.97 Hz
POINT 32768
FREQU 8278.15 Hz
SCANS 16
ACQTM 0.0000 sec
PD 0.0000 sec
PW1 10.00 usec
IRNUC
CTEMP 22.2 c
SLVNT CDCl3
EXREF 0.00 ppm
BF 0.00 Hz
RGAIN 362

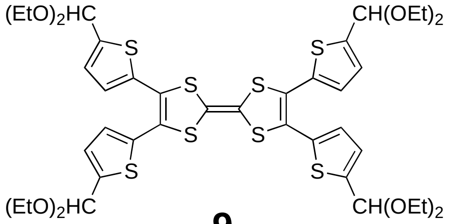

(EtO)2HC
CH(OEt)2
(EtO)2HC
CH(OEt)2

9

^{1}H NMR of **10**

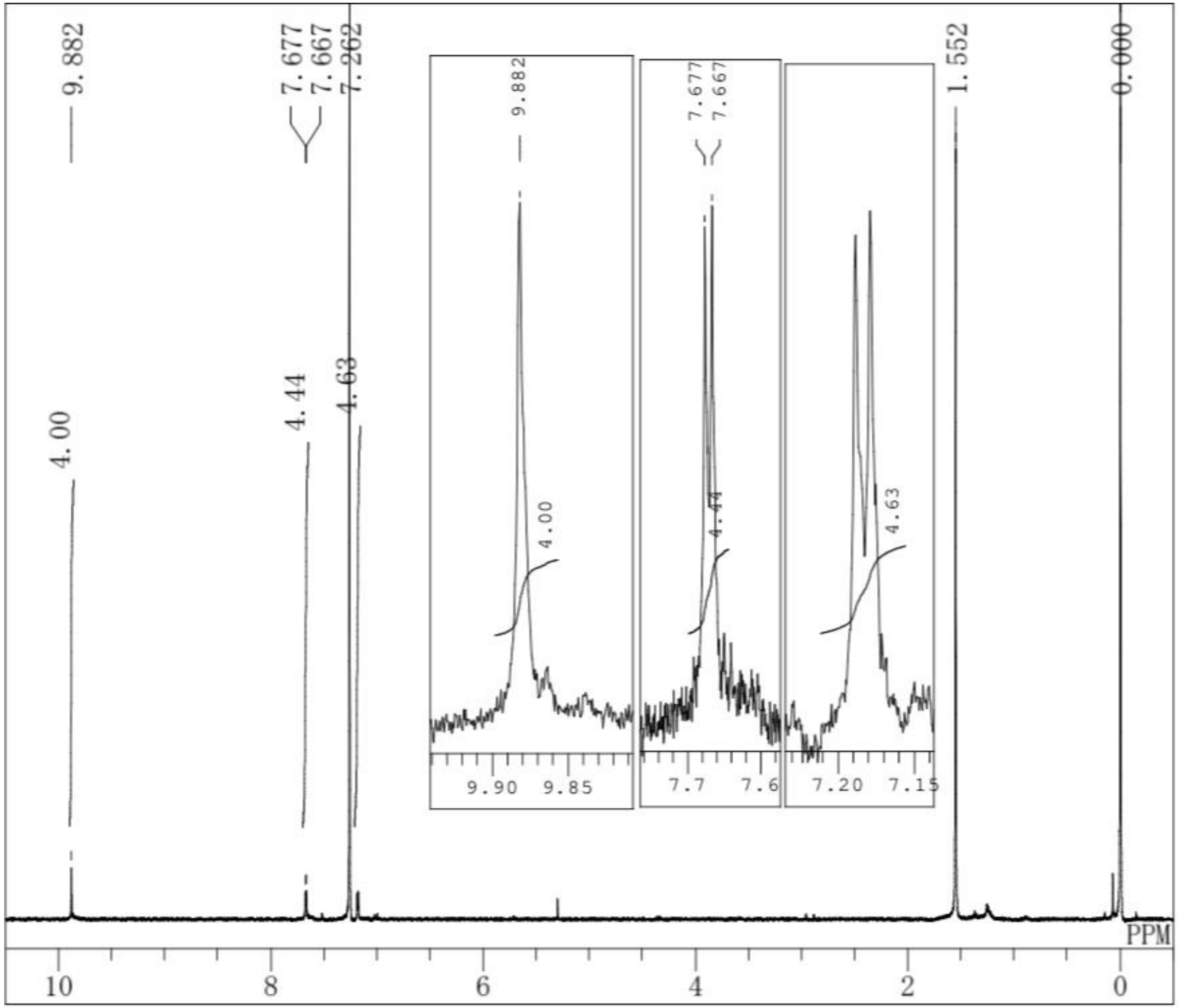

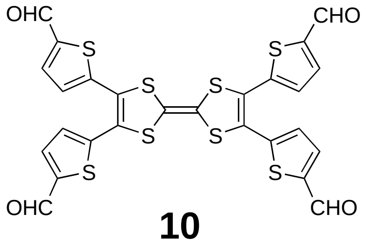

10

^{1}H NMR of **12**

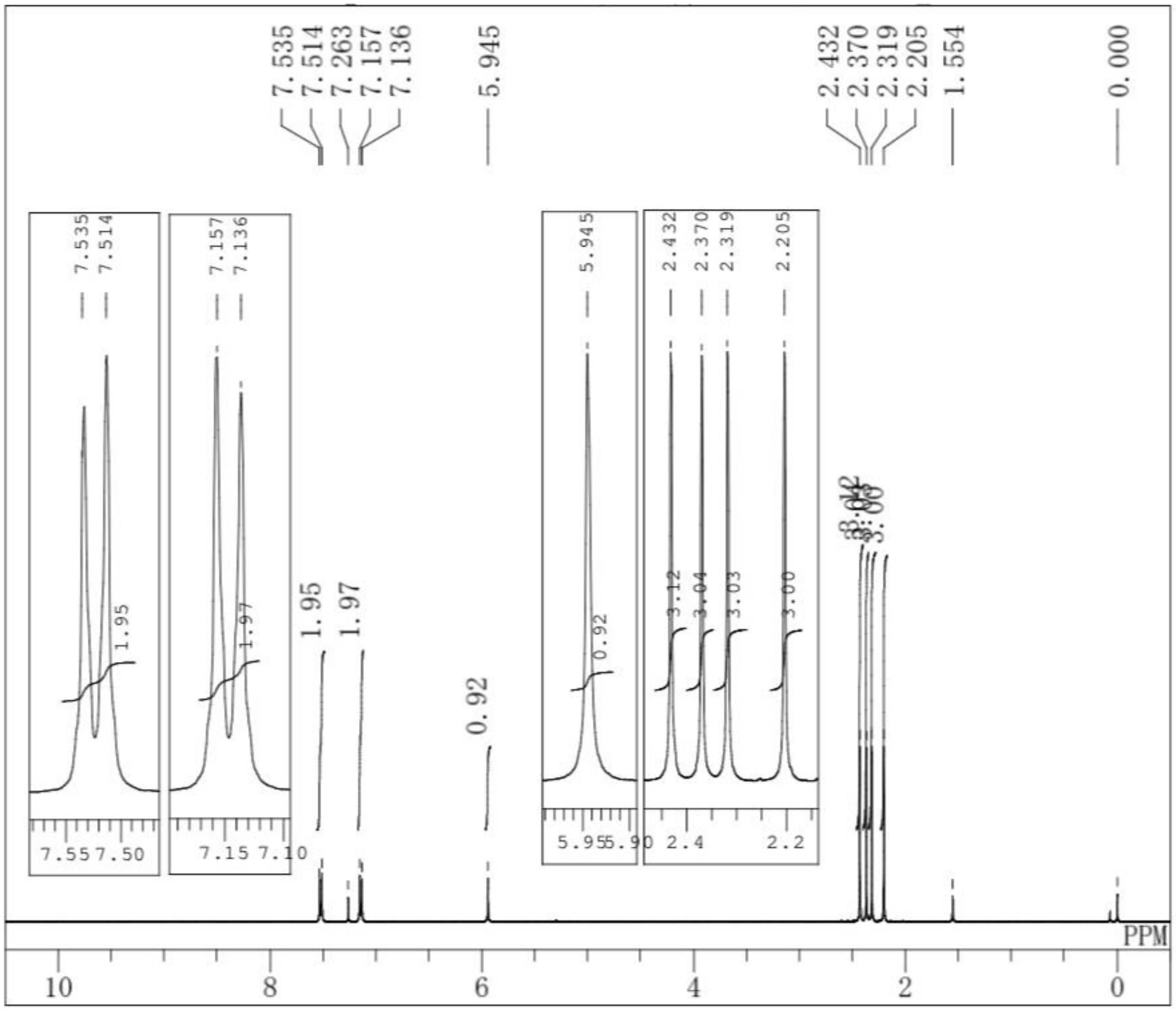

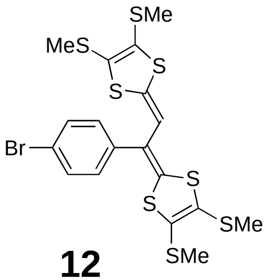

12

^{13}C NMR of **12**

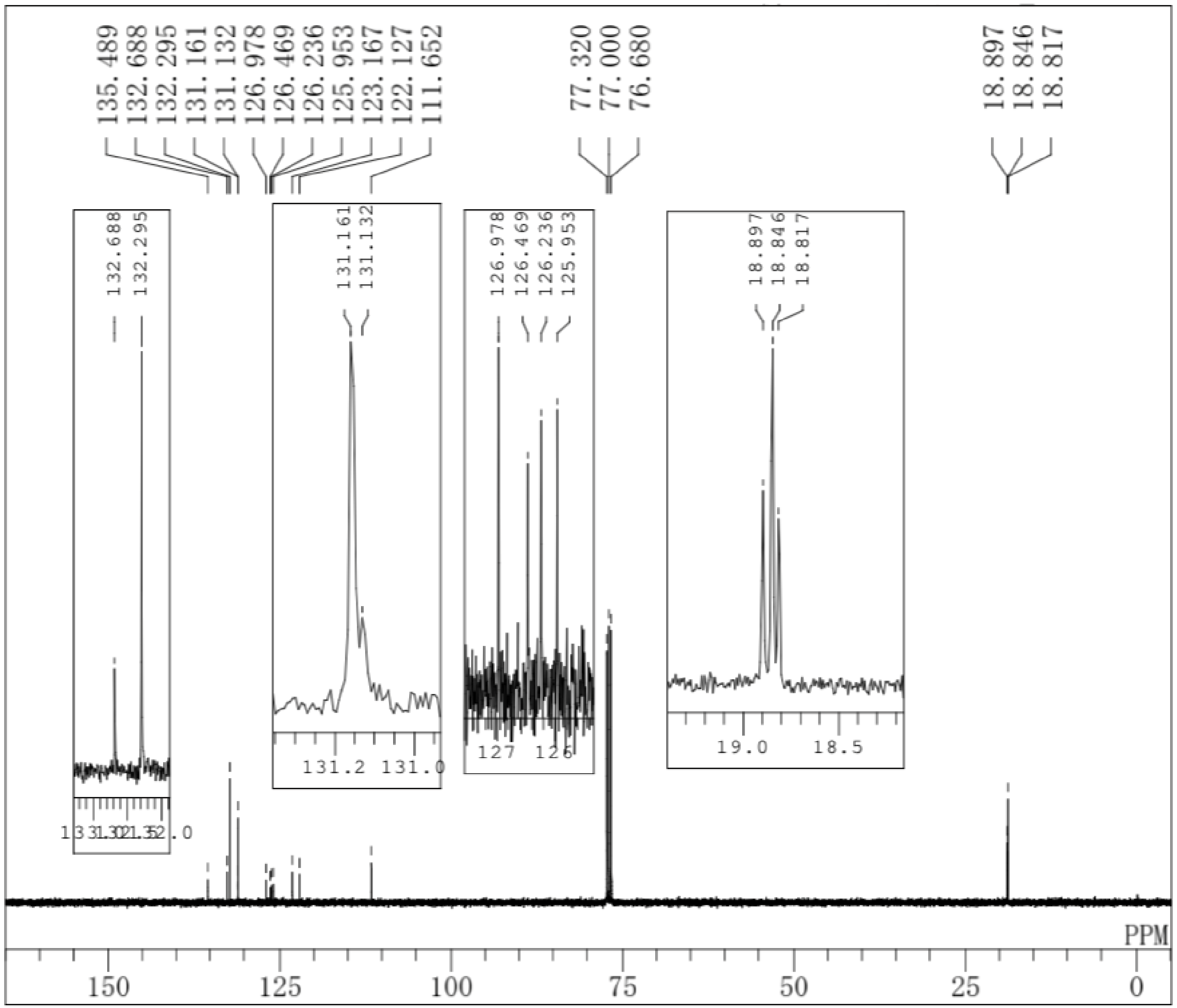

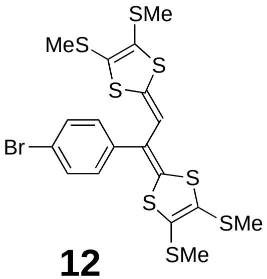

12

^{1}H NMR of **13**

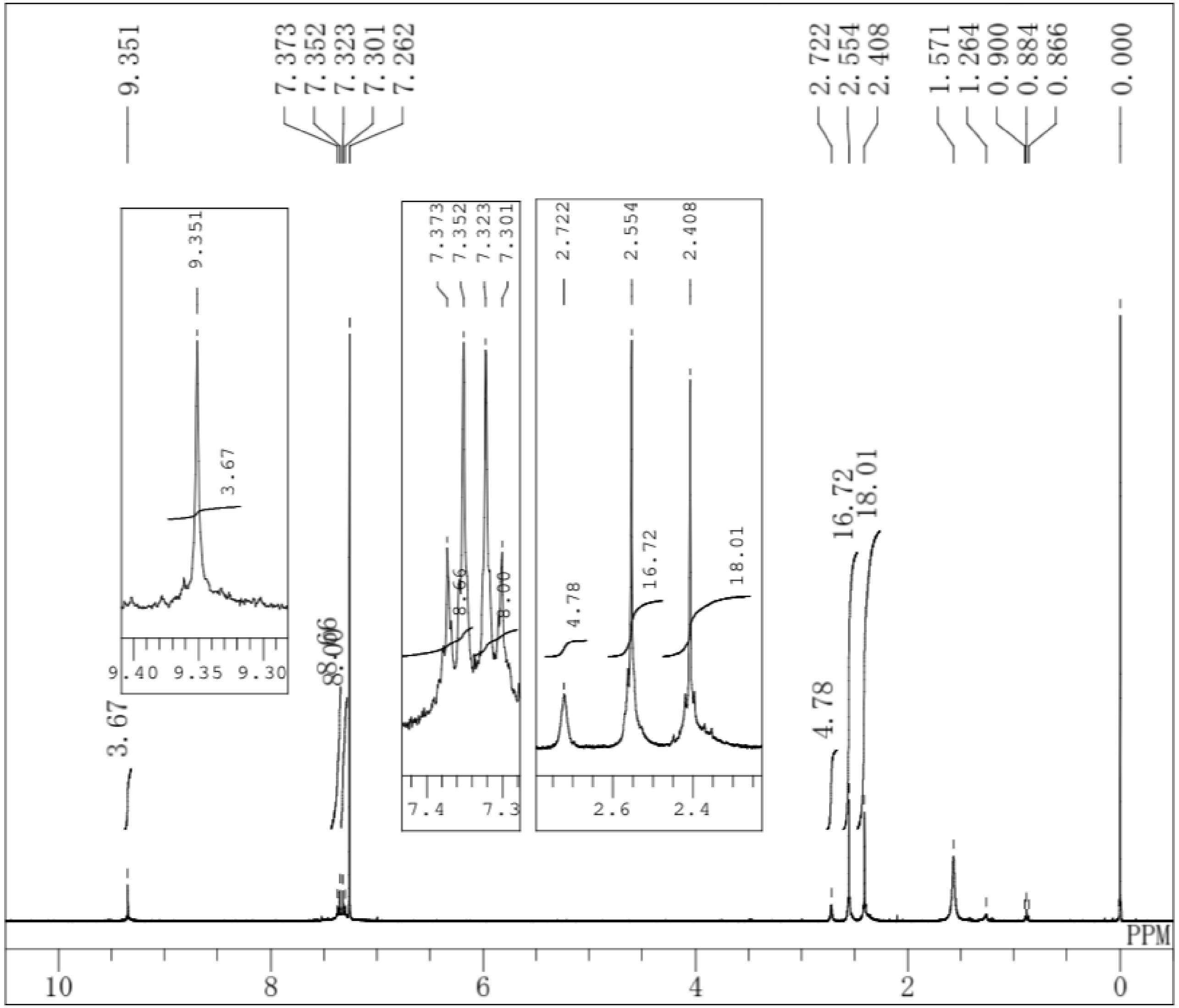

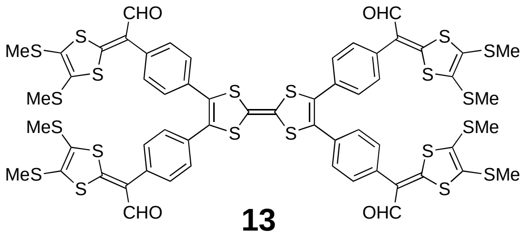

13

^{1}H NMR of **21**

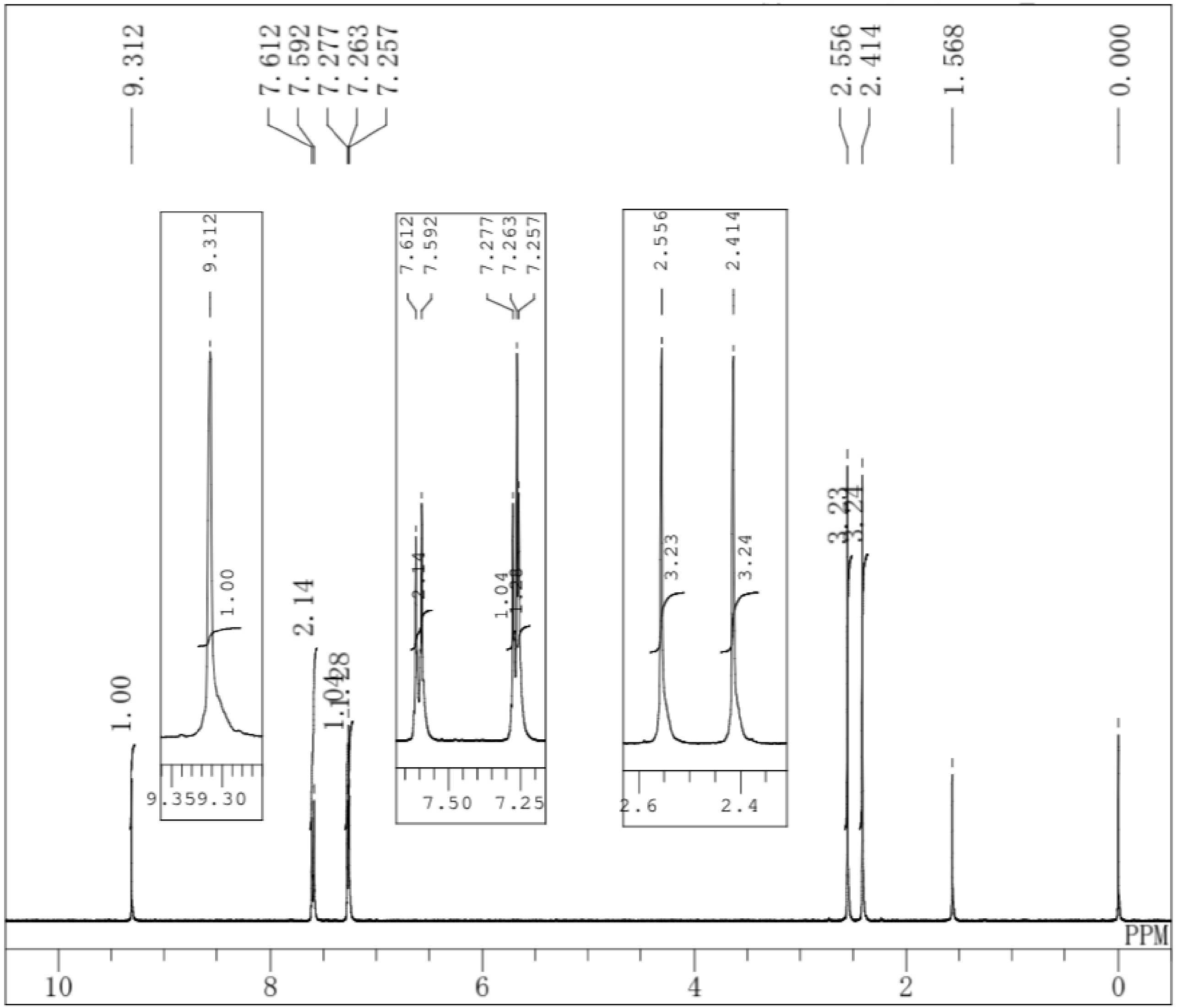

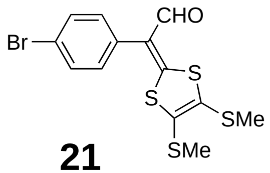

21

^{13}C NMR of **21**

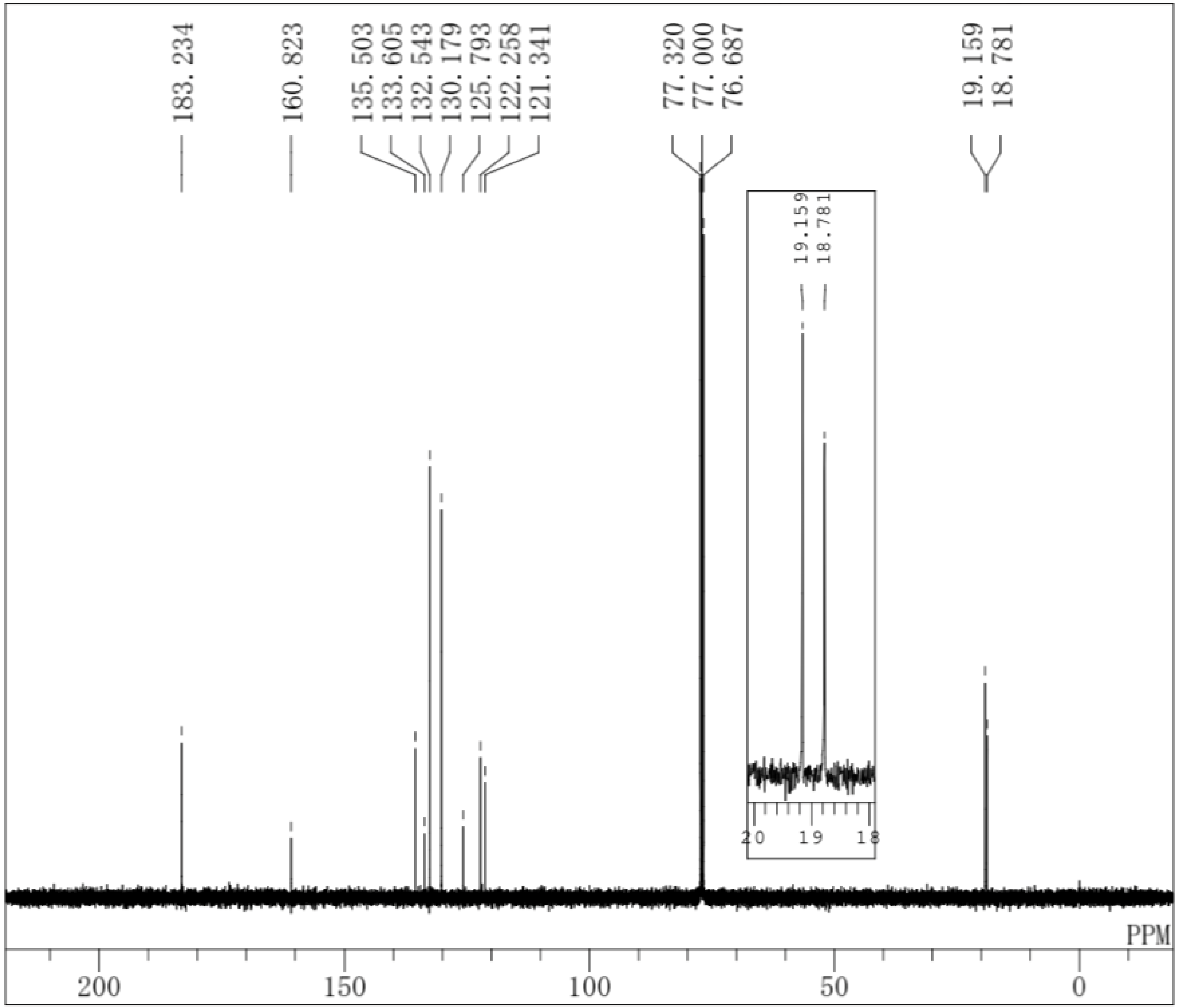

DFILE 08_4-PhBr-SMeDT-CHO
COMNT 4-PhBr-SMeDT-CHO
DATIM /prog/mod/proc1d /op
OBNUC 13C
EXMOD zgpg30
OBFRQ 100.62 MHz
OBSET 2.82 KHz
OBFIN 9.80 Hz
POINT 32768
FREQU 23980.81 Hz
SCANS 529
ACQTM 0.0000 sec
PD 0.0000 sec
PW1 10.00 usec
IRNUC
CTEMP 24.3 c
SLVNT CDCl3
EXREF 77.00 ppm
BF 0.12 Hz
RGAIN 18390

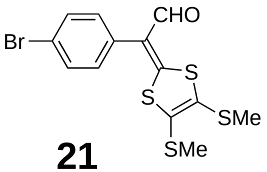

21
